# Supplementary material for: HPV16 Induces Formation of Virus-p62-PML Hybrid Bodies to Enable Infection
Source: Viruses. 2022 Jul 5;14(7):1478. doi: 10.3390/v14071478 (PMC9315800; doi:10.3390/v14071478)
Supplement: Supplementary file 1 [file viruses-14-01478-s001.zip › viruses-1774642-supplementary.pdf]

Supplementary Materials

HPV16 induces formation of virus-p62-PML hybrid bodies to enable infection

Linda Schweiger[1#], Laura A. Lelieveld-Fast[1#], Snježana Mikuličić[1#], Johannes Strunk[1], Kirsten Freitag[1], Stefan Tenzer[2], Albrecht M. Clement[3], and Luise Florin[1#]

1 Institute for Virology and Research Center for Immunotherapy (FZI), University Medical Center of the Johannes Gutenberg-University Mainz, Obere Zahlbacher Strasse 67, 55131, Mainz, Germany linda.schweiger@gmx.de (L.S.); laura.aline.fast@gmail.com (L.A.L.-F.); snjezana.mikulicic@gmail.com (S.M.); johannes.strunk@uni-mainz.de (J.S.:); kfreitag@uni-mainz.de (K.F.); lflorin@uni-mainz.de (L.F.)

2 Institute of Immunology, University Medical Centre, Johannes Gutenberg University of Mainz, Mainz, Germany; tenzer@uni-mainz.de (S.T.)

3 Institute of Pathobiochemistry, University Medical Center of the Johannes Gutenberg-University Mainz, Duesbergweg 6, 55128 Mainz, Germany; clement@uni-mainz.de (A.M.C.)

# L.S., L.A.F., and S.M. contributed equally to the manuscript

* Correspondence: lflorin@uni-mainz.de; Tel.: +49-(0)-6131-179083, LF

Supplementary Materials and Methods

Plasmids

Expression plasmid encoding LC3-GFP (pEGFP-C1/LC3-B full-length VK325) was a kind gift by Ivan Dikic (Institute of Biochemistry II, Goethe University Medical School, Frankfurt am Main, Germany).

Quantitative mass spectrometry

Protein digest preparation: Early endosomes were pelleted by ultracentrifugation (100.000 x g, 1 h, 4°C). Pelleted endosomes were solubilized in 25 mM ammonium bicarbonate containing 0.1 % RapiGest (Waters, Eschborn, Germany) (80°C, 15 min). Proteins were reduced by adding 5 mM DTT (45 min, 56 °C) and free cysteines alkylated with iodoacetamide (Sigma, Taufkirchen, Germany) (15 mM, 25°C, 1 h in dark). 0.2 µg porcine sequencing grade trypsin (Promega, Mannheim, Germany) were added and the samples were incubated overnight at 37°C. After digestion, RapiGest was hydrolyzed by adding 10 mM HCl (37°C, 10 min) and the resulting precipitate was removed by centrifugation (13.000 x g, 15 min, 4°C) and the supernatant was transferred into an autosampler vial for peptide analysis via LCMS.

UPLC configuration: Capillary liquid chromatography of tryptic peptides was performed with a Waters NanoAcquity UPLC system equipped with a 75 µm x 150 mm BEH C18 reversed phase column and a 2.6 µl PEEKSIL-sample loop (SGE, Darmstadt, Germany). The aqueous mobile phase (mobile phase A) was H2O (LC-MS Grade, Roth, Freiburg, Germany) with 0.1 % formic acid. The organic mobile phase (mobile phase B) was 0.1 % formic acid in acetonitrile (LC-MS grade, Roth). Samples (2.6 µl injection)

were loaded onto the column in direct injection mode with 3 % mobile phase B for 15 min at 400 nl/min, followed by an additional 10 min wash (3 % B) for 10 min at 300 nl/min. Peptides were eluted from the column with a gradient from 3-35 % mobile phase B over 90 min at 300 nl/min followed by a 20 min rinse of 80 % mobile phase B. The column was immediately re-equilibrated at initial conditions (3 % mobile phase B) for 20 min. [Glu1]fibrinopeptide was used as lockmass at 300 fmol/µl. Lockmass solution was delivered from the auxiliary pump of the NanoAcquity system at 400 nl/min to the reference sprayer of the NanoLockSprayTM source. Samples were analyzed in triplicate.

Mass spectrometer configuration: Mass spectrometry analysis of tryptic peptides was performed using a Waters Q-TOF Premier API system, operated in V-mode with typical resolving power of at least 10,000. All analyses were performed using positive mode ESI using a NanoLockSpray source. The lock mass channel was sampled every 30 s. The mass spectrometer was calibrated with a [Glu1]fibrinopeptide solution (300 fmol/µl) delivered through the reference sprayer of the NanoLockSpray source. Accurate mass LCMS data were collected in an alternating, low energy (MS) and elevated energy (MSE) mode of acquisition. The spectral acquisition time in each mode was 0.7 s with a 0.05-s interscan delay. In low energy MS mode, data were collected at constant collision energy of 3 eV. In MSE mode, collision energy was ramped from 16 to 36 eV during each 0.7 s data collection cycle. One cycle of MS and MSE data was acquired every 1.5 s. The radio frequency applied to the quadrupole mass analyzer was adjusted such that ions from m/z 300 to 1500 were efficiently transmitted, ensuring that any ions observed in the LC/MSE data less than m/z 300 were known to arise from dissociations in the collision cell.

Data processing and protein identification: The continuum LCMSE data were processed and searched using the IDENTITYE- Algorithm of ProteinLynx Global Server (PLGS) version 2.3. The resulting peptide and protein identifications were evaluated by the software using statistical models similar to those described by Skilling et al. [1]. Protein identifications were assigned by searching the UniProtKB/Swiss-Prot Protein Knowledgebase Release 52.3 for human proteins (12920 entries) supplemented with known possible contaminants and standard proteins (porcine trypsin, yeast enolase) using the precursor and fragmentation data afforded by the LCMS acquisition method as described before. The search parameter values for each precursor and associated fragment ions were set by the software using the measured mass error obtained from processing the raw continuum data. Peptide identifications were restricted to tryptic peptides with no more than one missed cleavage. Carbamidomethyl cysteine was set as fixed modification, and oxidized methionine, protein N-acetylation, and deamidation of asparagine and glutamine were searched as variable modifications. Database search was performed allowing a maximal mass deviation of 15 ppm for precursor ions and 30 ppm for fragment ions. For a valid protein identification, the following criteria had to be met: at least 2 peptides detected with together at least 7 fragments. All reported peptide identifications provided by

the IDENTITYE-algorithm are correct with >95% probability. The initial false positive rate for protein identification was set to 3 % based on search of a 5x randomized database, which was generated automatically using PLGS 2.3 by randomizing the primary amino acid sequence of each entry. Subsequently, by using a minimum replication rate of identification in two separate technical replicates as a filter, the false positive rate is further reduced to below 0.2%.

Western blot analysis of phosphorylated p62

For analysis of p62 phosphorylation, HeLa cells were seeded in a 12-well plate in DMEM medium with FCS (without antibiotics). On the next day, the cells were starved for 1 hour in DMEM without FCS prior to PsV addition. Afterwards, all tested samples were lysed at the same time in lysis buffer containing 5 mM Tris-HCl pH 7.4, 1 mM EGTA, 250 mM sucrose and 1% Triton X-100, and phosphatase inhibitor cocktail PhosSTOP (Roche, Penzberg, Germany). Samples underwent three freeze-thaw cycles (freezing at −80°C and thawing at 4°C), were transferred to the Eppis, centrifuged, and denatured at 95°C for 5 min in SDS sample buffer. Equal amounts of protein were loaded on an SDS–PAGE gel. The samples were electro-transferred onto a nitrocellulose membrane and blocked with 5% bovine serum albumin (BSA) in Tris-buffered saline supplemented with 0.01% Tween (TBST). After incubation with primary antibodies (in 5% BSA-TBST), proteins were detected using HRP-conjugated secondary antibodies. Detection was carried out using Western Lightning Plus ECL detection reagent (PerkinElmer, Waltham, MA). Signals were recorded with X-ray films for Western blot detection Super RX-N (Fujifilm, Duesseldorf, Germany).

Detection of L1-7 epitope by immunofluorescence

HeLa cells were grown on coverslips in a 12-well plate and transfected with siRNAs. 48 hours later cells were infected with HPV16 PsVs and incubated for 7 hours at 37 °C. Subsequently, the cells were fixed with methanol and processed for staining with mAb 33L1-7 (L1-7). This mAb recognizes a specific epitope located in the interior of the PsV capsid and is not accessible in intact virions [2]. The samples were analyzed by fluorescence microscopy using a Zeiss Axiovert 200M microscope and quantified by ImageJ software. Quantification of fluorescent pixels was performed with ImageJ software.

References

1. Skilling, J.; Denny, R.; Richardson, K.; Young, P.; McKenna, T.; Campuzano, I.; Ritchie, M. ProbSeq--a Fragmentation Model for Interpretation of Electrospray Tandem Mass Spectrometry Data. *Comparative and functional genomics* 2004, *5*, 61–68, doi:10.1002/cfg.370.

2. Spoden, G.; Freitag, K.; Husmann, M.; Boller, K.; Sapp, M.; Lambert, C.; Florin, L. Clathrin- and Caveolin-Independent Entry of Human Papillomavirus Type 16--Involvement of Tetraspanin-Enriched Microdomains (TEMs). *PLoS ONE* 2008, *3*, e3313, doi:10.1371/journal.pone.0003313.

Supplementary Figures

MS 1, HeLa

					MW Score			MW ppm			SD ppm					
Accession	Entry	Description	mW (Da)	pI (pH)	0h	4h	7h	0h	4h	7h	0h	4h	7h	NI → 4h	4h → 7h	NI → 7h
Q9H4L4	SENP3_HUMAN	Sentrin specific protease 3 EC 3 4 22 Sentrin SUMO specific protease SENP3 SUMO 1 specific pr	64968	8,5898		118	245		6020	6986			6848		1,16	
RANDOM5903	RANDOM5903	Random Sequence 5903	59244	9,1978		125	223		3358	4361		428			1,30	
P03101.2	VL1_HPV16	HPV16 Major capsid protein L1	59435	7,9459		722	1400		1774	3306		347	219		1,86	
P55283	CADH4_HUMAN	Cadherin 4 precursor Retinal cadherin R cadherin R CAD Homo sapiens Human	100217	4,4557		142	119		2438	2106		1522			0,86	
P63267	ACTH_HUMAN	Actin gamma enteric smooth muscle Smooth muscle gamma actin Gamma 2 actin Alpha actin 3 Hom	41849	5,1594			10776			2065			652			
P11488	GNAT1_HUMAN	Guanine nucleotide binding protein G t alpha 1 subunit Transducin alpha 1 chain Homo sapiens	40015	5,2696		1388	1443		689	1662		164	1219		2,41	
Q99808	S29A1_HUMAN	Equilibrative nucleoside transporter 1 Equilibrative nitrobenzylmercaptopurine riboside sensitive n	50186	8,2727		179	353		914	1157			294		1,27	
P60033	CD81_HUMAN	CD81 antigen 26 kDa cell surface protein TAPA 1 Target of the antiproliferative antibody 1 Tetr	25792	4,9124		1425	613		1269	900		164			0,71	
P20338	RAB4A_HUMAN	Ras related protein Rab 4A Homo sapiens Human	23856	5,7224		3261	1906		295	700		35			2,37	
P59190	RAB15_HUMAN	Ras related protein Rab 15 Homo sapiens Human	24375	5,3915		1184	2018		916	659		625	491		0,72	
Q6ZMR3	LDH6A_HUMAN	L lactate dehydrogenase A like 6A EC 1 1 1 27 Homo sapiens Human	36484	6,5543		272	152		3444	611			433		0,18	
Q9Y2Q5	MAPIP_HUMAN	Mitogen activated protein binding protein interacting protein Late endosomal lysosomal Mp1 interact	13498	5,1074		502	3364		513	608			229		1,18	
Q92930	RAB8B_HUMAN	Ras related protein Rab 8B Homo sapiens Human	23569	9,4318			3254			591			151			
P13929	ENOB_HUMAN	Beta enolase EC 4 2 1 11 2 phospho D glycerate hydro lyase Muscle specific enolase MSE Skel	46957	7,658		780	377		733	529		279			0,72	
P30483	1B45_HUMAN	HLA class I histocompatibility antigen B 45 alpha chain precursor MHC class I antigen B 45 Bw 45	40389	6,0086		2640	2123		189	450			410		2,39	
Q15771	RAB30_HUMAN	Ras related protein Rab 30 Homo sapiens Human	23043	4,7109		3380	2313		291	413		95	11		1,42	
Q86YS6	RAB43_HUMAN	Ras related protein Rab 43 Ras related protein Rab 41 Homo sapiens Human	23324	5,3031		3716	2234		306	408		39	199		1,33	
Q9Y2B0	MSAP_HUMAN	MIR interacting saposin like protein precursor Transmembrane protein 4 Putative secreted protein	20639	4,6177		2391	900		568	408		161	0		0,72	
P48666	K2C6C_HUMAN	Keratin type II cytoskeletal 6C Cytokeratin 6C CK 6C K6c keratin Homo sapiens Human	60163	8,0537		825	500		944	400			248		0,42	
O43657	TSN6_HUMAN	Tetraspanin 6 Tspan 6 Transmembrane 4 superfamily member 6 T245 protein Tetraspanin TM4 D A	27545	8,0574		395	405		470	396		101			0,84	
Q31612	1B73_HUMAN	HLA class I histocompatibility antigen B 73 alpha chain precursor MHC class I antigen B 73 Homo	40409	5,801		3911	3651		234	225		37			0,96	
O76013	K1H6_HUMAN	Keratin type I cuticular Ha6 Hair keratin type I Ha6 Homo sapiens Human	52214	4,7049	106	372	403	377	2305	4002				6,12	1,74	10,62
P18463	1B37_HUMAN	HLA class I histocompatibility antigen B 37 alpha chain precursor MHC class I antigen B 37 Homo	40431	5,7193	5746	7915	5906	108	588	1140		481	1720	5,44	1,94	10,54
P13646	K1C13_HUMAN	Keratin type I cytoskeletal 13 Cytokeratin 13 CK 13 Keratin 13 K13 Homo sapiens Human	49555	4,712	395	678	531	317	638	2773	35	229	3279	2,01	4,35	8,76
Q9BRK4	LZTS2_HUMAN	Leucine zipper putative tumor suppressor 2 Protein LAPSER1 Homo sapiens Human	72714	6,0859	104		99	436		3230	188			0,00		7,41
P84077	ARF1_HUMAN	ADP ribosylation factor 1 Homo sapiens Human	20683	6,3746	4305	985	951	193	450	1132		484	544	2,32	2,52	5,85
P63027	VAMP2_HUMAN	Vesicle associated membrane protein 2 VAMP 2 Synaptobrevin 2 Homo sapiens Human	12640	8,7006	2336		2047	138		772	38			0,00		5,61
Q05524	ENO1B_HUMAN	Alpha enolase lung specific EC 4 2 1 11 2 phospho D glycerate hydro lyase Non neural enolase	49446	5,705	264	480	303	234	1395	1296		572	159	5,97	0,93	5,54
P35749	MYH11_HUMAN	Myosin 11 Myosin heavy chain 11 Myosin heavy chain smooth muscle isoform SMMHC Homo sapiens	227197	5,2498	691	250	479	2538	10981	12373	1032	11708	5953	4,33	1,13	4,88
Q9H9B4	SFXN1_HUMAN	Sideroflexin 1 Tricarboxylate carrier protein TCC Homo sapiens Human	35596	9,3448	417		513	663		3125	30		763	0,00		4,71
P07900	HS90A_HUMAN	Heat shock protein HSP 90 alpha HSP 86 Renal carcinoma antigen NY REN 38 Homo sapiens Human	84606	4,747	1074	889	751	1457	3703	6592	548	423	3622	2,54	1,78	4,53
Q9Y5M8	SRPRB_HUMAN	Signal recognition particle receptor subunit beta SR beta Protein APMCF1 Homo sapiens Human	29683	9,3957	763	717	465	463	404	2079	65	48	2320	0,87	5,14	4,50
Q13501	SQSTM_HUMAN	p62/Sequestosome 1 Phosphotyrosine independent ligand for the Lck SH2 domain of 62 kDa Ubiquitin bind	47656	4,9312	1860	4152	5004	2091	5417	9147	72	301	1345	2,59	1,69	4,37
P23229	ITA6_HUMAN	Integrin alpha 6 precursor VLA 6 CD49f antigen Contains Integrin alpha 6 heavy chain Integrin	126539	6,3675	154	112	248	1179	2872	5131				2,44	1,79	4,35
P15153	RAC2_HUMAN	Ras related C3 botulinum toxin substrate 2 precursor p21 Rac2 Small G protein GX Homo sapien	21415	7,5566	1008	4783	3457	270	1326	1117	52	167	278	4,92	0,84	4,14
Q01546	K22O_HUMAN	Keratin type II cytoskeletal 2 oral Cytokeratin 2P K2P CK 2P Keratin 76 Homo sapiens Hum	65830	8,0982	260	299	443	301	642	1181		246	728	2,14	1,84	3,93
P41219	PERI_HUMAN	Peripherin Homo sapiens Human	53618	5,2088	154		363	254		988	86		513	0,00		3,89
P12035	K2C3_HUMAN	Keratin type II cytoskeletal 3 Cytokeratin 3 CK 3 Keratin 3 K3 65 kDa cytokeratin Homo	64464	6,0699	400	207	468	261	806	1011	9		340	3,09	1,25	3,87
P08727	K1C19_HUMAN	Keratin type I cytoskeletal 19 Cytokeratin 19 CK 19 Keratin 19 K19 Homo sapiens Human	44065	4,8591	2720	1632	2290	616	1005	2127	274	248	1890	1,63	2,12	3,45
Q9NSB2	KRT84_HUMAN	Keratin type II cuticular Hb4 Type II hair keratin Hb4 Keratin 84 K84 Homo sapiens Human	64854	7,6144	678	410	816	410	677	1382	32		249	1,65	2,04	3,37
P62820	RAB1A_HUMAN	Ras related protein Rab 1A YPT1 related protein Homo sapiens Human	22663	5,8482	10135		5671	512		1665	48			0,00		3,25
P12036	NFH_HUMAN	Neurofilament heavy polypeptide NF H Neurofilament triplet H protein 200 kDa neurofilament prot	112411	5,764	61	88	83	537	1039	1732		213	952	1,94	1,67	3,23
Q29960	1C16_HUMAN	HLA class I histocompatibility antigen Cw 16 alpha chain precursor MHC class I antigen Cw 16 Ho	40727	6,0786	4638		3114	528		1705	65			0,00		3,23
Q08043	ACTN3_HUMAN	Alpha actinin 3 Alpha actinin skeletal muscle isoform 3 F actin cross linking protein Homo sap	103229	5,2518	514	844	650	860	1589	2740	371	781	3300	1,85	1,72	3,19
P31947	1433S_HUMAN	14 3 3 protein sigma Stratifin Epithelial cell marker protein 1 Homo sapiens Human	27756	4,4822	652		524	117		371			42	0,00		3,16
P13637	AT1A3_HUMAN	Sodium potassium transporting ATPase alpha 3 chain EC 3 6 3 9 Sodium pump 3 Na K ATPase 3	111677	5,0544	3839	3738	2572	872	3656	2728	226	2526	1732	4,19	0,75	3,13
P02533	K1C14_HUMAN	Keratin type I cytoskeletal 14 Cytokeratin 14 CK 14 Keratin 14 K14 Homo sapiens Human	51589	4,8973	3404	2357	3319	462	848	1438	276	354	690	1,83	1,70	3,11
P04062	GLCM_HUMAN	Glucosylceramidase precursor EC 3 2 1 45 Beta glucocerebrosidase Acid beta glucosidase D gluc	59678	7,3423	291	598	425	1090	2201	3289	194	613	1840	2,02	1,49	3,02
P61020	RAB5B_HUMAN	Ras related protein Rab 5B Homo sapiens Human	23691	8,2381	3469	1320	178	277	643	828	124	259		2,32	1,29	2,99
Q7Z794	K2C1B_HUMAN	Keratin type II cytoskeletal 1b Keratin 77 Homo sapiens Human	61650	5,5001	822	115	450	494	983	1456	188		130	1,99	1,48	2,95
P21926	CD9_HUMAN	CD9 antigen p24 Leukocyte antigen MIC3 Motility related protein MRP 1 Tetraspanin 29 Tspa	25398	6,9016	209	605	974	317	485	930		50		1,53	1,92	2,94
O43504	XIP_HUMAN	Hepatitis B virus X interacting protein HBX interacting protein HBV X interacting protein Homo	9607	4,498	4432	4380	6950	213	447	622	31	53	80	2,10	1,39	2,92
Q14532	K1H2_HUMAN	Keratin type I cuticular Ha2 Hair keratin type I Ha2 Keratin 32 Homo sapiens Human	50286	4,6132	316	250	293	467	1791	1351			1065	3,84	0,75	2,89
P08107	HSP71_HUMAN	Heat shock 70 kDa protein 1 HSP70 1 HSP70 1 HSP70 2 Homo sapiens Human	69995	5,3154	1765	1856	2498	1096	1378	3106	409	631	457	1,26	2,25	2,83
Q13813	SPTA2_HUMAN	Spectrin alpha chain brain Spectrin non erythroid alpha chain Alpha II spectrin Fodrin alpha	284362	5,0519	259	119	165	6534	9903	18495	2547	462	734	1,52	1,87	2,83
P50993	AT1A2_HUMAN	Sodium potassium transporting ATPase alpha 2 chain precursor EC 3 6 3 9 Sodium pump 2 Na K	112193	5,3243	2896	2782	2173	1205	1054	3352	238	275	1922	0,88	3,18	2,78
Q13733	AT1A4_HUMAN	Sodium potassium transporting ATPase alpha 4 chain EC 3 6 3 9 Sodium pump 4 Na K ATPase 4	114093	6,1998	1186	582	592	1025	1619	2738	361	868	1703	1,58	1,69	2,67
O00560	SDCB1_HUMAN	Syntenin 1 Syndecan binding protein 1 Melanoma differentiation associated protein 9 MDA 9 Sca	32423	7,353	2054	1114	1066	922	1727	2447	335	1311	1173	1,87	1,42	2,65
P02769	ALBU_BOVIN	Serum albumin precursor Allergen Bos d 6 BSA	69248	5,7574	152	484	781	798	1342	2095	223	469	1086	1,68	1,56	2,63
P26232	CTNA2_HUMAN	Catenin alpha 2 Alpha catenin related protein Alpha N catenin Homo sapiens Human	105246	5,3741	319	309	252	903	1274	2326	365	394	1688	1,41	1,83	2,58
P32119	PRDX2_HUMAN	Peroxiredoxin 2 EC 1 11 1 15 Thioredoxin peroxidase 1 Thioredoxin dependent peroxide reductase	21878	5,5671	495	1498	2487	238	246	607			222	1,03	2,47	2,55
Q9Y4L1	OXRP_HUMAN	150 kDa oxygen regulated protein precursor Orp150 Hypoxia up regulated 1 Homo sapiens Human	111266	4,9713	713	258	192	4286	6919	10790	3162	2881		1,61	1,56	2,52
Q16352	AINX_HUMAN	Alpha internexin Alpha Inx 66 kDa neurofilament protein Neurofilament 66 NF 66 Homo sapien	55357	5,1687	84	45	175	401	683	1008			715	1,71	1,48	2,52
P62834	RAP1A_HUMAN	Ras related protein Rap 1A precursor GTP binding protein smg p21A Ras related protein Krev 1 C2	20973	6,5314	6000		7316	454		1138	175		436	0,00		2,51
Q6IAA8	CK059_HUMAN	UPF0404 protein C11orf59 Homo sapiens Human	17733	4,8312	5486	4176	3842	500	774	1242	97	67	326	1,55	1,60	2,48
P05787	K2C8_HUMAN	Keratin type II cytoskeletal 8 Cytokeratin 8 CK 8 Keratin 8 K8 Homo sapiens Human	53671	5,3399	13509	3232	9698	5395	3585	13298	372	273	838	0,66	3,71	2,46
P63104	1433Z_HUMAN	14 3 3 protein zeta delta Protein kinase C inhibitor protein 1 KCIP 1 Homo sapiens Human	27727	4,5273	3682	2957	3810	531	807	1306	55	124	122	1,52	1,62	2,46
Q04695	K1C17_HUMAN	Keratin type I cytoskeletal 17 Cytokeratin 17 CK 17 Keratin 17 K17 39 1 Homo sapiens H	48076	4,7767	10685	5467	11980	4525	2735	10811	529	293	1169	0,60	3,95	2,39
P08729	K2C7_HUMAN	Keratin type II cytoskeletal 7 Cytokeratin 7 CK 7 Keratin 7 K7 Sarcolectin Homo sapiens	51386	5,311	10226	3099	11018	4422	2565	10281	280	241	527	0,58	4,01	2,32
P08670	VIME_HUMAN	Vimentin Homo sapiens Human	53619	4,8629	2574	1070	3069	1931	1234	4465	316	298	2302	0,64	3,62	2,31
Q14254	FLOT2_HUMAN	Flotillin 2 Epidermal surface antigen ESA Homo sapiens Human	41659	5,0414	956	624	438	763	982	1764	121	193	642	1,29	1,80	2,31
O75923	DYSF_HUMAN	Dysferlin Dystrophy associated fer 1 like protein Fer 1 like protein 1 Homo sapiens Human	237142	5,3042	57	25	69	1711	3137	3898	425		1608	1,83	1,24	2,28
P38405	GNAL_HUMAN	Guanine nucleotide binding protein G olf subunit alpha Adenylate cyclase stimulating G alpha prote	44280	6,2172	784	1347	1352	362	1659	824	129	949	437	4,58	0,50	2,27
P49411	EFTU_HUMAN	Elongation factor Tu mitochondrial precursor EF Tu P43 Homo sapiens Human	49510	7,3718	558		215	907		2057	150		815	0,00		2,27
O14983	AT2A1_HUMAN	Sarcoplasmic endoplasmic reticulum calcium ATPase 1 EC 3 6 3 8 Calcium pump 1 SERCA1 SR Ca 2	110181	4,8796	474	259	218	1143	2215	2568	413	1343	1714	1,94	1,16	2,25
O75369	FLNB_HUMAN	Filamin B FLN B Beta filamin Actin binding like protein Thyroid autoantigen Truncated actin	278018	5,3729	166	113	138	4427	15361	9940	549	6213	4493	3,47	0,65	2,25
P54707	AT12A_HUMAN	Potassium transporting ATPase alpha chain 2 EC 3 6 3 10 Proton pump Non gastric H K ATPas	115825	6,0972	200	169	124	1896	1885	4250	736	1128	6194	0,99	2,25	2,24
P11279	LAMP1_HUMAN	Lysosome associated membrane glycoprotein 1 precursor LAMP 1 CD107a antigen Homo sapiens Huma	44744	9,2657	3441	4137	3751	4434	8719	9887	1136	836	1208	1,97	1,13	2,23
Q9NRW1	RAB6B_HUMAN	Ras related protein Rab 6B Homo sapiens Human	23446	5,2158	665	1358	385	338	1894	750				5,61	0,40	2,22
P13987	CD59_HUMAN	CD59 glycoprotein precursor Membrane attack complex inhibition factor MACIF MAC inhibitory prot	14167	6,0326	8482	8621	7731	973	2175	2119	122	304	317	2,23	0,97	2,18
Q7Z406	MYH14_HUMAN	Myosin 14 Myosin heavy chain 14 Myosin heavy chain nonmuscle IIc Nonmuscle myosin heavy chain	227861	5,6316	286	134	308	3747	4095	7957	2108	1510	3944	1,09	1,94	2,12
P18085	ARF4_HUMAN	ADP ribosylation factor 4 Homo sapiens Human	20497	7,0356	2966	1840	1323	587	669	1233	25	155	386	1,14	1,84	2,10
P10620	MGST1_HUMAN	Microsomal glutathione S transferase 1 EC 2 5 1 18 Microsomal GST 1 Microsomal GST I Homo sa	17587	9,6361	4297	4116	2742	1202	1969	2506	70	275		1,64	1,27	2,08
Q9H0U4	RAB1B_HUMAN	Ras related protein Rab 1B Homo sapiens Human	22157	5,3941	9852	8179	6685	737	1033	1533	209	313	156	1,40	1,48	2,08
P00338	LDHA_HUMAN	L lactate dehydrogenase A chain EC 1 1 1 27 LDH A LDH muscle subunit LDH M Proliferation in	36665	8,3652	3212	2919	5321	860	1067	1778	131	133	263	1,24	1,67	2,07
P62158	CALM_HUMAN	Calmodulin CaM Homo sapiens Human	16826	3,8824	12915	6021	12018	992	1019	2031	85	165	89	1,03	1,99	2,05
P08779	K1C16_HUMAN	Keratin type I cytoskeletal 16 Cytokeratin 16 CK 16 Keratin 16 K16 Homo sapiens Human	51236	4,7902	1914	1515	1572	349	522	714		71	194	1,50	1,37	2,05
P13747	HLAE_HUMAN	HLA class I histocompatibility antigen alpha chain E precursor MHC class I antigen E Homo sapie	40131	5,534	4198	3302	1911	804	1032	1637	606	227		1,28	1,59	2,04
Q13636	RAB31_HUMAN	Ras related protein Rab 31 Rab 22B Homo sapiens Human	21554	6,6667	287	267	306	223	324	454				1,45	1,40	2,03
P17661	DESM_HUMAN	Desmin Homo sapiens Human	53503	5,0295	238	369	568	251	367	511			164	1,46	1,39	2,03
P30462	1B14_HUMAN	HLA class I histocompatibility antigen B 14 alpha chain precursor MHC class I antigen B 14 Homo	40332	5,4573	8414		4703	781		1575	407		1966	0,00		2,02
Q9BSJ8	FA62A_HUMAN	Protein FAM62A Membrane bound C2 domain containing protein Homo sapiens Human	122780	5,4573	339	442	226	1821	2662	3646	190	392	1562	1,46	1,37	2,00
Q9BVK6	TMED9_HUMAN	Transmembrane emp24 domain containing protein 9 precursor Glycoprotein 25L2 Homo sapiens Human	25088	6,7819	3228	3479	2558	824	946	1649	82	118		1,15	1,74	2,00
Q96AX2	RAB37_HUMAN	Ras related protein Rab 37 Homo sapiens Human	24799	5,9365	1295	1900	1271	395	611	786		323	652	1,55	1,29	1,99
P13797	PLST_HUMAN	Plastin 3 T plastin Homo sapiens Human	70391	5,3943	908	493	479	1075	1602	2137	57	499	326	1,49	1,33	1,99
P13647	K2C5_HUMAN	Keratin type II cytoskeletal 5 Cytokeratin 5 CK 5 Keratin 5 K5 58 kDa cytokeratin Homo	62339	7,7926	412	503	425	420	1023	822	161	123	263	2,44	0,80	1,96
Q14126	DSG2_HUMAN	Desmoglein 2 precursor HDGC Homo sapiens Human	122308	4,9796	967	643	199	3313	3016	6479	422	373		0,91	2,15	1,96
P19013	K2C4_HUMAN	Keratin type II cytoskeletal 4 Cytokeratin 4 CK 4 Keratin 4 K4 Homo sapiens Human	57249	6,2153	668	416	404	660	713	1286		211	711	1,08	1,80	1,95
Q96AC1	PKHC1_HUMAN	Pleckstrin homology domain containing family C member 1 Kindlin 2 Mitogen inducible gene 2 protei	77810	6,2466	380	346	217	1060	1749	2060	49	121		1,65	1,18	1,94
P12235	ADT1_HUMAN	ADP ATP translocase 1 Adenine nucleotide translocator 1 ANT 1 ADP ATP carrier protein 1 Solut	33043	10,0719	1785	847	776	274	521	532	80		216	1,90	1,02	1,94
P62988	UBIQ_HUMAN	Ubiquitin Homo sapiens Human	8559	7,6652	38148	20763	32251	2582	2745	4980	446	381	1008	1,06	1,81	1,93
P19012	K1C15_HUMAN	Keratin type I cytoskeletal 15 Cytokeratin 15 CK 15 Keratin 15 K15 Homo sapiens Human	49137	4,513	1921	1029	1851	593	903	1132	288	219	405	1,52	1,25	1,91
P61981	1433G_HUMAN	14 3 3 protein gamma Protein kinase C inhibitor protein 1 KCIP 1 Homo sapiens Human	28284	4,606	1423	2037	1187	267	436	510	102	146	109	1,63	1,17	1,91
P29966	MARCS_HUMAN	Myristoylated alanine rich C kinase substrate MARCKS Protein kinase C substrate 80 kDa protein	31535	4,257	1003	1153	1090	562	903	1072	79	154	197	1,61	1,19	1,91
P62258	1433E_HUMAN	14 3 3 protein epsilon 14 3 3E Homo sapiens Human	29155	4,4355	2479	3174	3027	603	695	1145	38	89	239	1,15	1,65	1,90
Q99714	HCD2_HUMAN	3 hydroxyacyl CoA dehydrogenase type 2 EC 1 1 1 35 3 hydroxyacyl CoA dehydrogenase type II Type	26906	7,8378	296		583	262		497			27	0,00		1,90
Q15293	RCN1_HUMAN	Reticulocalbin 1 precursor Homo sapiens Human	38866	4,6729	1521	1390	1192	992	1338	1878	81	231	127	1,35	1,40	1,89
P36873	PP1G_HUMAN	Serine threonine protein phosphatase PP1 gamma catalytic subunit EC 3 1 3 16 PP 1G Protein phos	36959	6,1005	348	264	409	601	756	1131			85	1,26	1,50	1,88
Q9NZM1	MYOF_HUMAN	Myoferlin Fer 1 like protein 3 Homo sapiens Human	234558	5,7638	1667	901	699	10074	11684	18725	1378	1185	3955	1,16	1,60	1,86

P35900	K1C20_HUMAN	Keratin type I cytoskeletal 20 Cytokeratin 20 CK 20 Keratin 20 K20 Protein IT Homo sapi	48456	5,3912	944	766	940	652	794	1212	325	783	1406	1,22	1,53	1,86
Q9UBR2	CATZ_HUMAN	Cathepsin Z precursor EC 3 4 22 Cathepsin X Cathepsin P Homo sapiens Human	33846	6,7363	649	334	954	582	744	1081	89	116	223	1,28	1,45	1,86
Q99456	K1C12_HUMAN	Keratin type I cytoskeletal 12 Cytokeratin 12 CK 12 Keratin 12 K12 Homo sapiens Human	53478	4,5073	218	89	162	453	555	840	122		148	1,23	1,51	1,85
P54709	AT1B3_HUMAN	Sodium potassium transporting ATPase subunit beta 3 Sodium potassium dependent ATPase beta 3 subuni	31492	8,4351	3589	1929	914	1155	1718	2133	193	462	389	1,49	1,24	1,85
P34931	HS70L_HUMAN	Heat shock 70 kDa protein 1L Heat shock 70 kDa protein 1 like Heat shock 70 kDa protein 1 Hom H	70331	5,6453	2518	1848	1633	834	1356	1527	101		466	1,63	1,13	1,83
P19022	CADH2_HUMAN	Cadherin 2 precursor Neural cadherin N cadherin CD325 antigen CDw325 Homo sapiens Human	99747	4,442	661	401	701	3303	4079	6041	296	1085	1685	1,24	1,48	1,83
Q93084	AT2A3_HUMAN	Sarcoplasmic endoplasmic reticulum calcium ATPase 3 EC 3 6 3 8 Calcium pump 3 SERCA3 SR Ca 2	113904	5,2626	26	229	59	925	2096	1688	186	1142	38	2,26	0,81	1,82
P61421	VA0D_HUMAN	Vacuolar ATP synthase subunit d EC 3 6 3 14 V ATPase d subunit Vacuolar proton pump subunit d	40303	4,7043	603	1494	738	834	1352	1502	201	304	349	1,62	1,11	1,80
P04792	HSPB1_HUMAN	Heat shock protein beta 1 HspB1 Heat shock 27 kDa protein HSP 27 Stress responsive protein 27	22768	5,9588	758	2498	1055	289	582	516		93	26	2,02	0,89	1,79
P31946	1433B_HUMAN	14 3 3 protein beta alpha Protein kinase C inhibitor protein 1 KCIP 1 Protein 1054 Homo sapi	28064	4,5665	1324	1683	910	254	355	454	103	53	76	1,40	1,28	1,79
P31327	CPSM_HUMAN	Carbamoyl phosphate synthase ammonia mitochondrial precursor EC 6 3 4 16 Carbamoyl phosphate s	164834	6,2785	626		275	3225		5748	307		196	0,00		1,78
P12814	ACTN1_HUMAN	Alpha actinin 1 Alpha actinin cytoskeletal isoform Non muscle alpha actinin 1 F actin cross lin	102992	5,0927	1961	1549	1418	1344	1477	2387	164	335	895	1,10	1,62	1,78
P51149	RAB7A_HUMAN	Ras related protein Rab 7a Homo sapiens Human	23474	6,574	11834	17585	16446	1979	3587	3510	397	238	204	1,81	0,98	1,77
P11142	HSP7C_HUMAN	Heat shock cognate 71 kDa protein Heat shock 70 kDa protein 8 Homo sapiens Human	70854	5,1998	4644	4527	3948	3866	4785	6736	495	655	527	1,24	1,41	1,74
P05783	K1C18_HUMAN	Keratin type I cytoskeletal 18 Cytokeratin 18 CK 18 Keratin 18 K18 Homo sapiens Human	48028	5,1674	3572	305	1886	1588	500	2755	157	179	373	0,31	5,51	1,73
P62937	PPIA_HUMAN	Peptidyl prolyl cis trans isomerase A EC 5 2 1 8 PPIase A Rotamase A Cyclophilin A Cyclospo	18000	7,8525	2351	1538	1617	769	935	1330	103		250	1,22	1,42	1,73
P07237	PDIA1_HUMAN	Protein disulfide isomerase precursor EC 5 3 4 1 PDI Prolyl 4 hydroxylase subunit beta Cellul	57080	4,5643	10179	6615	8203	4392	5568	7548	650	504	825	1,27	1,36	1,72
Q9HAV0	GBB4_HUMAN	Guanine nucleotide binding protein subunit beta 4 Transducin beta chain 4 Homo sapiens Human	37543	5,527	2136	1543	727	318	325	540	39	22	215	1,02	1,66	1,69
P61769	B2MG_HUMAN	Beta 2 microglobulin precursor Contains Beta 2 microglobulin variant pI 5 3 Homo sapiens Human	13705	6,0716	12780	12583	11733	1005	1671	1671	192	415	498	1,66	1,00	1,66
P21281	VATB2_HUMAN	Vacuolar ATP synthase subunit B brain isoform EC 3 6 3 14 V ATPase B2 subunit Vacuolar proton	56464	5,4487	456	636	426	1217	2243	2023	164	551		1,84	0,90	1,66
Q12797	ASPH_HUMAN	Aspartyl asparaginyl beta hydroxylase EC 1 14 11 16 Aspartate beta hydroxylase ASP beta hydroxy	85809	4,7338	603	806	711	2546	2959	4225	265	607	8	1,16	1,43	1,66
Q9Y228	T3JAM_HUMAN	TRAF3 interacting JNK activating modulator TRAF3 interacting protein 3 Homo sapiens Human	63586	8,5594	124	94	138	557	1166	923				2,09	0,79	1,66
Q14108	SCRB2_HUMAN	Lysosome membrane protein 2 Lysosome membrane protein II LIMP II Scavenger receptor class B mem	54255	4,8171	1253	1400	1515	1434	2645	2375	464	266		1,85	0,90	1,66
Q9UBI6	GBG12_HUMAN	Guanine nucleotide binding protein G I G S G O gamma 12 subunit precursor Homo sapiens Human	8001	9,4759	19447	20608	22152	582	852	955	83	168	112	1,46	1,12	1,64
Q15836	VAMP3_HUMAN	Vesicle associated membrane protein 3 VAMP 3 Synaptobrevin 3 Cellubrevin CEB Homo sapiens	11301	9,4081	4898		2115	394		645	53		54	0,00		1,64
Q9UJZ1	STML2_HUMAN	Stomatin like protein 2 SLP 2 EPB72 like 2 Homo sapiens Human	38510	7,2642	625		512	623		1015	96		118	0,00		1,63
P27348	1433T_HUMAN	14 3 3 protein theta 14 3 3 protein tau 14 3 3 protein T cell HS1 protein Homo sapiens Huma	27746	4,4848	1242	1246	1152	257	270	414	163	84	110	1,05	1,54	1,61
P14618	KPYM_HUMAN	Pyruvate kinase isozymes M1 M2 EC 2 7 1 40 Pyruvate kinase muscle isozyme Pyruvate kinase 2 3	57900	7,7532	4152	1735	2709	3119	3749	5031	180	266	320	1,20	1,34	1,61
P07195	LDHB_HUMAN	L lactate dehydrogenase B chain EC 1 1 1 27 LDH B LDH heart subunit LDH H Renal carcinoma a	36615	5,6393	3023	2824	4754	1026	818	1652	78	119	105	0,80	2,02	1,61
P46776	RL27A_HUMAN	60S ribosomal protein L27a Homo sapiens Human	16551	11,4214	1611	1357	1032	468	446	754	89	35		0,95	1,69	1,61
Q14315	FLNC_HUMAN	Filamin C Gamma filamin Filamin 2 Protein FLNc Actin binding like protein ABP L ABP 280 l	290775	5,5997	62	55	65	5291	5788	8491	469	1151	2356	1,09	1,47	1,60
P38606	VATA_HUMAN	Vacuolar ATP synthase catalytic subunit A EC 3 6 3 14 V ATPase subunit A Vacuolar proton pump a	68260	5,1846	1108	1248	836	1260	1768	2016	304	445	515	1,40	1,14	1,60
P07602	SAP_HUMAN	Proactivator polypeptide precursor Contains Saposin A Protein A Saposin B Val Saposin B Sphin	58073	4,8853	5123	5557	6629	6960	8795	11102	959	1273	709	1,26	1,26	1,60
P30447	1A23_HUMAN	HLA class I histocompatibility antigen A 23 alpha chain precursor MHC class I antigen A 23 A 9	40707	5,8793	6569	5632	5261	93	129	148	12			1,39	1,15	1,59
P48047	ATPO_HUMAN	ATP synthase O subunit mitochondrial precursor EC 3 6 3 14 Oligomycin sensitivity conferral prot	23262	10,3627	1547		458	519		815	113		211	0,00		1,57
P35609	ACTN2_HUMAN	Alpha actinin 2 Alpha actinin skeletal muscle isoform 2 F actin cross linking protein Homo sap	103788	5,1522	589	676	907	1532	1939	2400	897	1097	1980	1,27	1,24	1,57
P07686	HEXB_HUMAN	Beta hexosaminidase beta chain precursor EC 3 2 1 52 N acetyl beta glucosaminidase Beta N acety	63071	6,2966	453		380	1077		1670	95			0,00		1,55
Q02818	NUCB1_HUMAN	Nucleobindin 1 precursor CALNUC Homo sapiens Human	53846	4,9794	1061	520	864	1101	1244	1701	175	276	194	1,13	1,37	1,54
P18859	ATP5J_HUMAN	ATP synthase coupling factor 6 mitochondrial precursor EC 3 6 3 14 ATPase subunit F6 Homo sap	12579	9,9822	1517	616	1366	260	146	400				0,56	2,74	1,54
O43854	EDIL3_HUMAN	EGF like repeat and discoidin I like domain containing protein 3 precursor EGF like repeats and dis	53729	6,9921	1021	895	613	960	1488	1466	109	229	422	1,55	0,99	1,53
P11717	MPRI_HUMAN	Cation independent mannose 6 phosphate receptor precursor CI Man 6 P receptor CI MPR M6PR Ins	274097	5,5177	220	206	149	6197	11263	9427	812	4103	4781	1,82	0,84	1,52
Q14525	K1HB_HUMAN	Keratin type I cuticular Ha3 II Hair keratin type I Ha3 II Homo sapiens Human	46184	4,6091	624	106	182	558	620	847				1,11	1,37	1,52
P07099	HYEP_HUMAN	Epoxide hydrolase 1 EC 3 3 2 9 Microsomal epoxide hydrolase Epoxide hydratase Homo sapiens	52914	6,8355	1567	1489	486	898	1560	1342	175	161	124	1,74	0,86	1,49
O43707	ACTN4_HUMAN	Alpha actinin 4 Non muscle alpha actinin 4 F actin cross linking protein Homo sapiens Human	104788	5,1204	5048	3438	2733	5406	7282	8065	393	350	1280	1,35	1,11	1,49
Q06830	PRDX1_HUMAN	Peroxiredoxin 1 EC 1 11 1 15 Thioredoxin peroxidase 2 Thioredoxin dependent peroxide reductase	22096	8,2425	4726	2938	3847	1148	1206	1713	224	89	376	1,05	1,42	1,49
P12236	ADT3_HUMAN	ADP ATP translocase 3 Adenine nucleotide translocator 2 ANT 3 ADP ATP carrier protein 3 Solut	32845	10,0611	3172	1268	2440	744	416	1108	132	113	244	0,56	2,66	1,49
P11021	GRP78_HUMAN	78 kDa glucose regulated protein precursor GRP 78 Heat shock 70 kDa protein 5 Immunoglobulin he	72288	4,8748	16774	14412	13265	14346	22193	21324	809	761	1333	1,55	0,96	1,49
O15427	MOT4_HUMAN	Monocarboxylate transporter 4 MCT 4 MCT 3 Solute carrier family 16 member 3 Homo sapiens Hu	49436	7,8856	1600	1161	526	2316	3107	3435	383	393	340	1,34	1,11	1,48
Q9Y277	VDAC3_HUMAN	Voltage dependent anion selective channel protein 3 VDAC 3 hVDAC3 Outer mitochondrial membrane	30639	8,8301	1549	1290	1169	773	809	1145	135	188	165	1,05	1,41	1,48
P61106	RAB14_HUMAN	Ras related protein Rab 14 Homo sapiens Human	23881	5,7979	5511	6050	5903	1183	2234	1752	98	300	138	1,89	0,78	1,48
P30499	1C01_HUMAN	HLA class I histocompatibility antigen Cw 1 alpha chain precursor MHC class I antigen Cw 1 Homo	40939	5,3665	3587	5432	5641	1772	2649	2619		524	1752	1,49	0,99	1,48
P63096	GNAI1_HUMAN	Guanine nucleotide binding protein G i alpha 1 subunit Adenylate cyclase inhibiting G alpha prote	40335	5,59	1727	1978	1511	526	612	774	56	165	84	1,17	1,26	1,47
P32969	RL9_HUMAN	60S ribosomal protein L9 Homo sapiens Human	21849	10,3702	1509		1181	423		623	94		34	0,00		1,47
P35232	PHB_HUMAN	Prohibitin Homo sapiens Human	29785	5,4293	3716	934	2717	885	496	1297	70	166	297	0,56	2,61	1,47
P61006	RAB8A_HUMAN	Ras related protein Rab 8A Oncogene c mel Homo sapiens Human	23653	9,4318	5526	5846	2797	469	798	683	120	166		1,70	0,86	1,46
Q9BQE3	TBA6_HUMAN	Tubulin alpha 6 chain Alpha tubulin 6 Homo sapiens Human	49863	4,7856	1214	1583	1676	1311	2159	1900			1055	1,65	0,88	1,45
P07197	NFM_HUMAN	Neurofilament medium polypeptide NF M Neurofilament triplet M protein 160 kDa neurofilament pro	102386	4,7007	86	27	136	1821	877	2620	1509		2205	0,48	2,99	1,44
Q15907	RB11B_HUMAN	Ras related protein Rab 11B GTP binding protein YPT3 Homo sapiens Human	24473	5,5464	4228	2406	826	876	1910	1256	111	1255	56	2,18	0,66	1,43
Q8TCJ2	STT3B_HUMAN	Dolichyl diphosphooligosaccharide protein glycosyltransferase subunit STT3B EC 2 4 1 119 Oligosa	93613	9,0547	173	316	116	1172	3332	1679	1			2,84	0,50	1,43
P15328	FOLR1_HUMAN	Folate receptor alpha precursor FR alpha Folate receptor 1 Folate receptor adult Adult folat	29799	7,8569	7692	4228	4903	4570	5698	6544	566	1456	875	1,25	1,15	1,43
P27105	STOM_HUMAN	Erythrocyte band 7 integral membrane protein Stomatin Protein 7 2b Homo sapiens Human	31710	7,9876	20325	15865	10662	4159	7514	5944	403	609	286	1,81	0,79	1,43
P51153	RAB13_HUMAN	Ras related protein Rab 13 Cell growth inhibiting gene 4 protein Homo sapiens Human	22759	9,5698	1881	1997	1131	600	703	857	277	241	414	1,17	1,22	1,43
P30511	HLAF_HUMAN	HLA class I histocompatibility antigen alpha chain F precursor HLA F antigen Leukocyte antigen F	40543	5,2372	946	1832	505	649	654	926		243		1,01	1,41	1,43
P16615	AT2A2_HUMAN	Sarcoplasmic endoplasmic reticulum calcium ATPase 2 EC 3 6 3 8 Calcium pump 2 SERCA2 SR Ca 2	114682	5,0515	1301	913	503	4017	6199	5720	374	1013	1037	1,54	0,92	1,42
P35222	CTNB1_HUMAN	Catenin beta 1 Beta catenin Homo sapiens Human	85442	5,4476	1573	808	671	1930	2447	2726	213	550	874	1,27	1,11	1,41
P07737	PROF1_HUMAN	Profilin 1 Profilin I Homo sapiens Human	15044	8,4591	2476	2303	1987	780	872	1098	145	282	431	1,12	1,26	1,41
Q15758	AAAT_HUMAN	Neutral amino acid transporter B 0 ATB 0 Solute carrier family 1 member 5 Sodium dependent ne	56562	5,1749	6651	5699	5284	6396	7774	8999	456	839	706	1,22	1,16	1,41
P13645	K1C10_HUMAN	Keratin type I cytoskeletal 10 Cytokeratin 10 CK 10 Keratin 10 K10 Homo sapiens Human	59482	4,9506	1236	3282	754	1060	3830	1487	186	266	276	3,61	0,39	1,40
P08174	DAF_HUMAN	Complement decay accelerating factor precursor CD55 antigen Homo sapiens Human	41373	7,4581	1348	2182	983	2351	4159	3284	178	636	809	1,77	0,79	1,40
Q07021	C1QBP_HUMAN	Complement component 1 Q subcomponent binding protein mitochondrial precursor Glycoprotein gC1qBP	31342	4,5458	2313		2296	944		1310	33		263	0,00		1,39
Q9HC07	TM165_HUMAN	Transmembrane protein 165 Transmembrane protein TPARL Transmembrane protein PT27 Homo sapiens	34883	6,6196	819	527	720	832	914	1154	123	146		1,10	1,26	1,39
P50897	PPT1_HUMAN	Palmitoyl protein thioesterase 1 precursor EC 3 1 2 22 PPT 1 Palmitoyl protein hydrolase 1 H	34171	6,065	1268	1135	664	1379	2056	1905	331	306		1,49	0,93	1,38
P02786	TFR1_HUMAN	Transferrin receptor protein 1 TfR1 TR TfR Trfr CD71 antigen T9 p90 Homo sapiens Hu	84818	6,1633	5493	4535	3085	8564	12459	11727	368	1005	1028	1,45	0,94	1,37
P05141	ADT2_HUMAN	ADP ATP translocase 2 Adenine nucleotide translocator 2 ANT 2 ADP ATP carrier protein 2 Solut	32874	10,0433	4667	2019	4010	1873	1042	2562	162	126	413	0,56	2,46	1,37
Q07065	CKAP4_HUMAN	Cytoskeleton associated protein 4 63 kDa membrane protein p63 Homo sapiens Human	65982	5,5415	1054	946	525	1534	2219	2096	101	186	287	1,45	0,94	1,37
O75955	FLOT1_HUMAN	Flotillin 1 Homo sapiens Human	47325	7,2968	1279	812	301	925	1179	1260	39	163	213	1,28	1,07	1,36
Q15582	BGH3_HUMAN	Transforming growth factor beta induced protein ig h3 precursor Beta ig h3 Kerato epithelin RGD	74634	7,5214	837	535	286	1633	1920	2223	203	296	619	1,18	1,16	1,36
P62873	GBB1_HUMAN	Guanine nucleotide binding protein G I G S G T subunit beta 1 Transducin beta chain 1 Homo sa	37353	5,5351	4457	4062	2360	1324	2229	1789	99	346	280	1,68	0,80	1,35
Q99805	TM9S2_HUMAN	Transmembrane 9 superfamily protein member 2 precursor p76 Homo sapiens Human	75725	7,1512	445	694	521	2336	1718	3151	125	704	672	0,74	1,83	1,35
P62879	GBB2_HUMAN	Guanine nucleotide binding protein G I G S G T subunit beta 2 Transducin beta chain 2 G protei	37307	5,5355	4830	3910	2529	2084	2831	2802	83	305	251	1,36	0,99	1,34
P21796	VDAC1_HUMAN	Voltage dependent anion selective channel protein 1 VDAC 1 hVDAC1 Outer mitochondrial membrane	30753	8,8669	10903	5973	4373	3321	3405	4458	40	286	464	1,03	1,31	1,34
P00533	EGFR_HUMAN	Epidermal growth factor receptor precursor EC 2 7 10 1 Receptor tyrosine protein kinase ErbB 1	134190	6,2333	565	392	331	2878	3994	3860	212	305	776	1,39	0,97	1,34
P08238	HS90B_HUMAN	Heat shock protein HSP 90 beta HSP 84 HSP 90 Homo sapiens Human	83212	4,7726	2979	2080	2077	4381	5188	5811	1370	1675	2467	1,18	1,12	1,33
P04844	RIB2_HUMAN	Dolichyl diphosphooligosaccharide protein glycosyltransferase 63 kDa subunit precursor EC 2 4 1 11	69241	5,332	2594	2460	1844	2492	3615	3300	458	252	351	1,45	0,91	1,32
P60174	TPIS_HUMAN	Triosephosphate isomerase EC 5 3 1 1 TIM Triose phosphate isomerase Homo sapiens Human	26652	6,5034	1643	500	1273	457	424	603	67	54	133	0,93	1,42	1,32
P05387	RLA2_HUMAN	60S acidic ribosomal protein P2 Renal carcinoma antigen NY REN 44 Homo sapiens Human	11657	4,1781	18426	6187	9540	746	640	979	41	89	70	0,86	1,53	1,31
O00592	PODXL_HUMAN	Podocalyxin like protein 1 precursor Homo sapiens Human	55561	5,2286	2512	2076	2046	3327	4221	4355	334	286	267	1,27	1,03	1,31
P13473	LAMP2_HUMAN	Lysosome associated membrane glycoprotein 2 precursor LAMP 2 CD107b antigen Homo sapiens Huma	44932	5,2308	2620	3026	1746	3390	5384	4424	269	877	883	1,59	0,82	1,31
P29777	GNAO2_HUMAN	Guanine nucleotide binding protein G o subunit alpha 2 Homo sapiens Human	40061	5,5148	1228	1782	1618	593	445	764			619	0,75	1,72	1,29
P07864	LDHC_HUMAN	L lactate dehydrogenase C chain EC 1 1 1 27 LDH C LDH testis subunit LDH X Homo sapiens H	36288	7,2303	170	64	237	296	805	381			8	2,72	0,47	1,29
P52907	CAZA1_HUMAN	F actin capping protein subunit alpha 1 CapZ alpha 1 Homo sapiens Human	32902	5,3258	614	166	160	397	653	508				1,65	0,78	1,28
P30101	PDIA3_HUMAN	Protein disulfide isomerase A3 precursor EC 5 3 4 1 Disulfide isomerase ER 60 ERp60 58 kDa mi	56746	5,9299	9508	10363	8192	5862	7437	7507	614	283	798	1,27	1,01	1,28
P00387	NCB5R_HUMAN	NADH cytochrome b5 reductase EC 1 6 2 2 B5R Diaphorase 1 Cytochrome b5 reductase 3 Contains	34212	7,3865	943	1525	946	1205	1516	1542	67	137	324	1,26	1,02	1,28
P17066	HSP76_HUMAN	Heat shock 70 kDa protein 6 Heat shock 70 kDa protein B Homo sapiens Human	70984	5,7312	684	959	998	2496	2412	3190	2692	2723	3859	0,97	1,32	1,28
P10909	CLUS_HUMAN	Clusterin precursor Complement associated protein SP 40 40 Complement cytolysis inhibitor CLI	52461	5,8378	978	1354	602	1190	1254	1513	157	119	5	1,05	1,21	1,27
Q00325	MPCP_HUMAN	Phosphate carrier protein mitochondrial precursor PTP Solute carrier family 25 member 3 Homo	40068	9,6387	943	148	467	1131	1207	1436	239			1,07	1,19	1,27
P26038	MOES_HUMAN	Moesin Membrane organizing extension spike protein Homo sapiens Human	67777	6,0095	4052	2959	1796	5018	6367	6371	596	550	819	1,27	1,00	1,27
O15260	SURF4_HUMAN	Surfeit locus protein 4 Homo sapiens Human	30373	7,8243	2182	2241	2298	891	902	1130	27			1,01	1,25	1,27
P61586	RHOA_HUMAN	Transforming protein RhoA precursor H12 Homo sapiens Human	21754	5,7296	8279	5874	7352	809	1032	1022	87	365	213	1,28	0,99	1,26
Q14697	GANAB_HUMAN	Neutral alpha glucosidase AB precursor EC 3 2 1 84 Glucosidase II subunit alpha Homo sapiens	106806	5,6876	1194	780	544	5617	7355	7090	695	1466	487	1,31	0,96	1,26
P17987	TCPA_HUMAN	T complex protein 1 subunit alpha TCP 1 alpha CCT alpha Homo sapiens Human	60305	5,7127	1178	336	291	1390	1254	1751	149	279		0,90	1,40	1,26
P48643	TCPE_HUMAN	T complex protein 1 subunit epsilon TCP 1 epsilon CCT epsilon Homo sapiens Human	59632	5,3036	1474	236	396	1483	1454	1868	226	250	802	0,98	1,28	1,26
Q8NBS9	TXND5_HUMAN	Thioredoxin domain containing protein 5 precursor Thioredoxin like protein p46 Endoplasmic reticu	47598	5,562	1537	1219	1458	1277	1211	1596	116	275		0,95	1,32	1,25
P35580	MYH10_HUMAN	Myosin 10 Myosin heavy chain 10 Myosin heavy chain nonmuscle IIb Nonmuscle myosin heavy chain	228796	5,2711	715	201	281	4281	3986	5338	1738	1389	1763	0,93	1,34	1,25
P35908	K22E_HUMAN	Keratin type II cytoskeletal 2 epidermal Cytokeratin 2e K2e CK 2e Keratin 2 Homo sapiens	65825	8,053	1246	1902	426	1631	2889	2032	796	193	670	1,77	0,70	1,25
Q99623	PHB2_HUMAN	Prohibitin 2 B cell receptor associated protein BAP37 Repressor of estrogen receptor activity D	33275	10,1891	544		523	613		763	56		64	0,00		1,24
P61224	RAP1B_HUMAN	Ras related protein Rap 1b precursor GTP binding protein smg p21B Homo sapiens Human	20811	5,4648	12030	10800	7400	1295	2454	1598	134	284	364	1,90	0,65	1,23
P60709	ACTB_HUMAN	Actin cytoplasmic 1 Beta actin Homo sapiens Human	41709	5,142	29898	29801	17045	79186	90574	97688		2654	8707	1,14	1,08	1,23
P21333	FLNA_HUMAN	Filamin A Alpha filamin Filamin 1 Endothelial actin binding protein Actin binding protein 280	280561	5,6364	2528	1551	1273	16553	20413	20347	1301	2148	2974	1,23	1,00	1,23
P15311	EZRI_HUMAN	Ezrin p81 Cytovillin Villin 2 Homo sapiens Human	69369	5,8713	1951	2309	1195	2412	2834	2964	337	219	770	1,17	1,05	1,23
P27797	CALR_HUMAN	Calreticulin precursor CRP55 Calregulin HACBP ERp60 grp60 Homo sapiens Human	48111	4,0941	8600	6755	7578	8894	11281	10925	673	1828	1517	1,27	0,97	1,23
P60660	MYL6_HUMAN	Myosin light polypeptide 6 Smooth muscle and nonmuscle myosin light chain alkali 6 Myosin light c	16919	4,3596	8261	5241	3507	1336	1119	1635	113	208	336	0,84	1,46	1,22

P25705	ATPA_HUMAN	ATP synthase subunit alpha mitochondrial precursor EC 3 6 3 14 Homo sapiens Human	59713	9,4308	8345	1450	5553	6106	3095	7467	338	1139	587	0,51	2,41	1,22
P20648	ATP4A_HUMAN	Potassium transporting ATPase alpha chain 1 EC 3 6 3 10 Proton pump Gastric H K ATPase su	114017	5,451	510	382	278	1692	3028	2066	939	2582	2415	1,79	0,68	1,22
Q9UBM7	DHCR7_HUMAN	7 dehydrocholesterol reductase EC 1 3 1 21 7 DHC reductase Sterol Delta 7 reductase Putative	54453	8,7156	149	221	359	897	1001	1094		497		1,12	1,09	1,22
Q8WTV0	SCRB1_HUMAN	Scavenger receptor class B member 1 SRB1 SR BI CD36 antigen like 1 CD36 and LIMPII analogous	60837	8,1691	275	186	247	1587	2370	1929	209			1,49	0,81	1,22
P62745	RHOB_HUMAN	Rho related GTP binding protein RhoB precursor H6 Homo sapiens Human	22109	4,9136	1923	2787	2395	275	413	333	46	103	89	1,50	0,81	1,21
P11166	GTR1_HUMAN	Solute carrier family 2 facilitated glucose transporter member 1 Glucose transporter type 1 eryth	54048	8,8607	1885	1353	1648	5242	6114	6345	280	532	637	1,17	1,04	1,21
Q10471	GALT2_HUMAN	Polypeptide N acetylgalactosaminyltransferase 2 EC 2 4 1 41 Protein UDP acetylgalactosaminyltrans	64691	8,3212	3931	1782	1679	3819	3432	4619	414	141	497	0,90	1,35	1,21
P30048	PRDX3_HUMAN	Thioredoxin dependent peroxide reductase mitochondrial precursor EC 1 11 1 15 Peroxiredoxin 3	27675	7,693	1053		478	337		406	13			0,00		1,20
P14314	GLU2B_HUMAN	Glucosidase 2 subunit beta precursor Glucosidase II subunit beta Protein kinase C substrate 60 1	59387	4,1307	2950	1954	1828	3584	3508	4292	184	596	952	0,98	1,22	1,20
P06576	ATPB_HUMAN	ATP synthase subunit beta mitochondrial precursor EC 3 6 3 14 Homo sapiens Human	56524	5,0955	9015	1906	5020	5614	2440	6665	448	307	693	0,43	2,73	1,19
Q9NVJ2	ARL8B_HUMAN	ADP ribosylation factor like protein 8B ADP ribosylation factor like protein 10C Novel small G pr	21525	8,6039	2208	1101	353	617	662	731	243	200		1,07	1,10	1,19
Q04917	1433F_HUMAN	14 3 3 protein eta Protein AS1 Homo sapiens Human	28201	4,5602	1056	546	298	236	250	279	47	101		1,06	1,11	1,18
P08648	ITA5_HUMAN	Integrin alpha 5 precursor Fibronectin receptor subunit alpha Integrin alpha F VLA 5 CD49e an	114464	5,3917	943	291	330	4839	5009	5705	581	1098		1,04	1,14	1,18
P20020	AT2B1_HUMAN	Plasma membrane calcium transporting ATPase 1 EC 3 6 3 8 PMCA1 Plasma membrane calcium pump iso	138667	5,6429	515	425	336	3470	1813	4080	1179		2579	0,52	2,25	1,18
Q08380	LG3BP_HUMAN	Galectin 3 binding protein precursor Lectin galactoside binding soluble 3 binding protein Mac 2 b	65289	4,9438	363	629	642	1628	2201	1891	486	994	229	1,35	0,86	1,16
Q96AG4	LRC59_HUMAN	Leucine rich repeat containing protein 59 Homo sapiens Human	34908	10,0276	3153	3894	1033	1547	2517	1796	333	176	356	1,63	0,71	1,16
P51148	RAB5C_HUMAN	Ras related protein Rab 5C RAB5L L1880 Homo sapiens Human	23467	8,5794	5037	3283	2156	837	1156	968	93	394	759	1,38	0,84	1,16
P23284	PPIB_HUMAN	Peptidyl prolyl cis trans isomerase B precursor EC 5 2 1 8 PPIase Rotamase Cyclophilin B S	22728	9,7617	9607	10073	5605	2588	3362	2988	247	326	923	1,30	0,89	1,15
P62424	RL7A_HUMAN	60S ribosomal protein L7a Surfeit locus protein 3 PLA X polypeptide Homo sapiens Human	29977	11,057	1019	494	499	1074	1088	1239	21	320		1,01	1,14	1,15
O75396	SC22B_HUMAN	Vesicle trafficking protein SEC22b SEC22 vesicle trafficking protein homolog B SEC22 vesicle traf	24724	8,7349	4562	4394	3931	749	827	860	324	337	475	1,10	1,04	1,15
P35579	MYH9_HUMAN	Myosin 9 Myosin heavy chain 9 Myosin heavy chain nonmuscle IIa Nonmuscle myosin heavy chain II	226390	5,3355	5611	1273	1767	16283	10167	18583	744	762	5931	0,62	1,83	1,14
P05023	AT1A1_HUMAN	Sodium potassium transporting ATPase alpha 1 chain precursor EC 3 6 3 9 Sodium pump 1 Na K	112824	5,1682	5947	5020	3997	14475	16977	16496	950	841	1607	1,17	0,97	1,14
Q09666	AHNK_HUMAN	Neuroblast differentiation associated protein AHNAK Desmoyokin Fragments Homo sapiens Human	312292	6,2635	1991	793	919	34054	31100	38759	4634	3641	3948	0,91	1,25	1,14
P19087	GNAT2_HUMAN	Guanine nucleotide binding protein G t alpha 2 subunit Transducin alpha 2 chain Homo sapiens	40150	4,9254	796	621	1130	829	722	939	377	13	110	0,87	1,30	1,13
P61916	NPC2_HUMAN	Epididymal secretory protein E1 precursor Niemann Pick disease type C2 protein hE1 Homo sapien	16559	7,6346	1489	1315	1393	508	327	576	82	73		0,64	1,76	1,13
P14923	PLAK_HUMAN	Junction plakoglobin Desmoplakin 3 Desmoplakin III Homo sapiens Human	81577	5,9187	2588	2071	1893	3319	4475	3760	368	759	944	1,35	0,84	1,13
P61026	RAB10_HUMAN	Ras related protein Rab 10 Homo sapiens Human	22526	8,5735	6192	7919	4500	1142	1661	1292	151	297	465	1,45	0,78	1,13
Q15084	PDIA6_HUMAN	Protein disulfide isomerase A6 precursor EC 5 3 4 1 Protein disulfide isomerase P5 Thioredoxin	48091	4,7615	4739	5736	4173	3041	3615	3433	339	339	316	1,19	0,95	1,13
Q13162	PRDX4_HUMAN	Peroxiredoxin 4 EC 1 11 1 15 Prx IV Thioredoxin peroxidase AO372 Thioredoxin dependent peroxi	30520	5,8337	1712	1099	1081	606	724	680	43	132	100	1,20	0,94	1,12
P08195	4F2_HUMAN	4F2 cell surface antigen heavy chain 4F2hc Lymphocyte activation antigen 4F2 large subunit 4F2	57908	5,0268	30828	24188	22617	29299	30042	32874	4741	2036	2638	1,03	1,09	1,12
P30050	RL12_HUMAN	60S ribosomal protein L12 Homo sapiens Human	17807	9,8965	3982	938	1573	462	486	519	36		79	1,05	1,07	1,12
P80723	BASP_HUMAN	Brain acid soluble protein 1 BASP1 protein Neuronal axonal membrane protein NAP 22 22 kDa neuro	22680	4,4231	29701	32891	35334	6049	7366	6753	953	877	1187	1,22	0,92	1,12
O43852	CALU_HUMAN	Calumenin precursor Crocalbin IEF SSP 9302 Homo sapiens Human	37083	4,2772	9552	2457	2283	2249	2031	2485	108	310	283	0,90	1,22	1,10
P63010	AP2B1_HUMAN	AP 2 complex subunit beta 1 Adapter related protein complex 2 beta 1 subunit Beta adaptin Plasm	104486	5,0544	217		156	1221		1348	91			0,00		1,10
P03989	1B27_HUMAN	HLA class I histocompatibility antigen B 27 alpha chain precursor MHC class I antigen B 27 Homo	40403	5,4439	4125		1393	957		1044	1092		612	0,00		1,09
Q9NP58	ABCB6_HUMAN	Mitochondrial ATP binding cassette sub family B member 6 Mitochondrial ABC transporter 3 Mt ABC t	93826	8,4981	313	310	168	2029	2133	2214	1081	198		1,05	1,04	1,09
P50990	TCPQ_HUMAN	T complex protein 1 subunit theta TCP 1 theta CCT theta Renal carcinoma antigen NY REN 15 Ho	59582	5,2714	2381	1134	827	2138	1966	2330	344	380	1284	0,92	1,19	1,09
P63000	RAC1_HUMAN	Ras related C3 botulinum toxin substrate 1 precursor p21 Rac1 Ras like protein TC25 Cell migrat	21436	8,5715	1547	3553	3229	951	1392	1029	112	42	678	1,46	0,74	1,08
P46940	IQGA1_HUMAN	Ras GTPase activating like protein IQGAP1 p195 Homo sapiens Human	189132	6,0403	1837	1214	1020	8527	9988	9229	609	926	1479	1,17	0,92	1,08
Q13740	CD166_HUMAN	CD166 antigen precursor Activated leukocyte cell adhesion molecule ALCAM Homo sapiens Human	65061	5,8473	1178	812	746	2316	2671	2503	356	291	390	1,15	0,94	1,08
O00159	MYO1C_HUMAN	Myosin Ic Myosin I beta MMI beta MMIb Homo sapiens Human	117965	9,7381	1036	793	532	3335	4705	3599	333	664	573	1,41	0,76	1,08
P04843	RIB1_HUMAN	Dolichyl diphosphooligosaccharide protein glycosyltransferase 67 kDa subunit precursor EC 2 4 1 11	68526	5,9266	2436	2216	1404	2816	4368	3020	222	1435	1003	1,55	0,69	1,07
Q6IQ22	RAB12_HUMAN	Ras related protein Rab 12 Homo sapiens Human	27231	8,5098	1096	1481	1434	282	283	300				1,01	1,06	1,07
P05026	AT1B1_HUMAN	Sodium potassium transporting ATPase subunit beta 1 Sodium potassium dependent ATPase beta 1 subuni	35038	8,6472	1276	878	1091	1935	2191	2041	224	454	325	1,13	0,93	1,05
P08754	GNAI3_HUMAN	Guanine nucleotide binding protein G k subunit alpha G i alpha 3 Homo sapiens Human	40506	5,3582	3934	4594	3931	2041	2859	2145	377	362	212	1,40	0,75	1,05
P35221	CTNA1_HUMAN	Catenin alpha 1 Cadherin associated protein Alpha E catenin NY REN 13 antigen Homo sapiens	100008	5,8751	3781	1824	1005	4150	4257	4339	397	619	236	1,03	1,02	1,05
P19105	MLRM_HUMAN	Myosin regulatory light chain 2 nonsarcomeric Myosin RLC Homo sapiens Human	19781	4,4518	4033	2085	1945	767	627	798	99	2	93	0,82	1,27	1,04
P61604	CH10_HUMAN	10 kDa heat shock protein mitochondrial Hsp10 10 kDa chaperonin CPN10 Early pregnancy factor	10924	9,4693	11605	3783	8126	1239	343	1283	160	60	155	0,28	3,74	1,04
P10809	CH60_HUMAN	60 kDa heat shock protein mitochondrial precursor Hsp60 60 kDa chaperonin CPN60 Heat shock p	61016	5,5503	21588	3831	14649	9501	4078	9833	915	325	513	0,43	2,41	1,03
P05388	RLA0_HUMAN	60S acidic ribosomal protein P0 L10E Homo sapiens Human	34251	5,6049	3535	1845	1333	1166	1053	1203	62	124	157	0,90	1,14	1,03
P54652	HSP72_HUMAN	Heat shock related 70 kDa protein 2 Heat shock 70 kDa protein 2 Homo sapiens Human	69977	5,4073	3179	3245	2641	1082	1744	1112	801	199	33	1,61	0,64	1,03
P06756	ITAV_HUMAN	Integrin alpha V precursor Vitronectin receptor subunit alpha CD51 antigen Contains Integrin a	115964	5,3102	409	651	426	3172	4221	3225	625	384	234	1,33	0,76	1,02
P16435	NCPR_HUMAN	NADPH cytochrome P450 reductase EC 1 6 2 4 CPR P450R Homo sapiens Human	76641	5,241	232	209	331	1362	1417	1383	643	212	196	1,04	0,98	1,02
O95573	ACSL3_HUMAN	Long chain fatty acid CoA ligase 3 EC 6 2 1 3 Long chain acyl CoA synthetase 3 LACS 3 Homo	80368	8,3719	403	311	293	1902	1724	1925		221		0,91	1,12	1,01
Q6YHK3	CD109_HUMAN	CD109 antigen precursor p180 150 KDa TGF beta 1 binding protein r150 Platelet specific Gov an	161586	5,4761	254		198	3386		3427	58		69	0,00		1,01
Q01650	LAT1_HUMAN	Large neutral amino acids transporter small subunit 1 L type amino acid transporter 1 4F2 light c	54974	7,6304	10987	7660	8089	11756	11885	11893	2022	1176	2782	1,01	1,00	1,01
P14625	ENPL_HUMAN	Endoplasmin precursor Heat shock protein 90 kDa beta member 1 94 kDa glucose regulated protein	92411	4,5643	8238	6456	4189	11377	14759	11488	534	523	1218	1,30	0,78	1,01
P43121	MUC18_HUMAN	Cell surface glycoprotein MUC18 precursor Melanoma associated antigen MUC18 Melanoma cell adhesio	71562	5,4534	1384	702	690	2934	2526	2961	608	222	912	0,86	1,17	1,01
P40926	MDHM_HUMAN	Malate dehydrogenase mitochondrial precursor EC 1 1 1 37 Homo sapiens Human	35508	8,8202	2234	159	257	1090	970	1095	135		105	0,89	1,13	1,00
P01891	1A68_HUMAN	HLA class I histocompatibility antigen A 68 alpha chain precursor MHC class I antigen A 68 Aw 68	40883	6,2267	10313	11278	8028	3503	4044	3494	457	577	1293	1,15	0,86	1,00
P06733	ENOA_HUMAN	Alpha enolase EC 4 2 1 11 2 phospho D glycerate hydro lyase Non neural enolase NNE Enolase	47139	7,1713	1949	1589	1095	1793	1685	1788	280	410	416	0,94	1,06	1,00
P30040	ERP29_HUMAN	Endoplasmic reticulum protein ERp29 precursor ERp31 ERp28 Homo sapiens Human	28975	7,2823	1599		503	634		624	198		138	0,00		0,98
Q03135	CAV1_HUMAN	Caveolin 1 Homo sapiens Human	20458	5,5891	11219	6345	5587	1318	1154	1294	229	184	127	0,88	1,12	0,98
P45880	VDAC2_HUMAN	Voltage dependent anion selective channel protein 2 VDAC 2 hVDAC2 Outer mitochondrial membrane	38068	6,3333	2086	1009	1351	1728	1320	1684	247	418	468	0,76	1,28	0,97
P15291	B4GT1_HUMAN	Beta 1 4 galactosyltransferase 1 EC 2 4 1 Beta 1 4 GalTase 1 Beta4Gal T1 b4Gal T1 UDP gal	43892	8,7054	1170	521	1010	1240	899	1196	76	133	62	0,73	1,33	0,96
Q6EMK4	VASN_HUMAN	Vasorin precursor Protein Slit like 2 Homo sapiens Human	71667	7,0876	265	358	245	1118	1986	1075	420	181		1,78	0,54	0,96
P27824	CALX_HUMAN	Calnexin precursor Major histocompatibility complex class I antigen binding protein p88 p90 IP9	67525	4,2675	6295	4080	2240	12323	12366	11662	889	1017	1182	1,00	0,94	0,95
P01893	HLAH_HUMAN	HLA class I histocompatibility antigen alpha chain H precursor HLA AR HLA 12 4 Homo sapiens	40824	5,8671	5741	6109	4928	2122	1654	2007	288	990	527	0,78	1,21	0,95
P68363	TBAK_HUMAN	Tubulin alpha ubiquitous chain Alpha tubulin ubiquitous Tubulin K alpha 1 Homo sapiens Human	50119	4,7622	832	2553	2072	2007	1676	1896	127	1023	856	0,84	1,13	0,94
P23528	COF1_HUMAN	Cofilin 1 Cofilin non muscle isoform 18 kDa phosphoprotein p18 Homo sapiens Human	18490	8,2196	10240	5758	5665	1556	1431	1461	423	126	85	0,92	1,02	0,94
Q07020	RL18_HUMAN	60S ribosomal protein L18 Homo sapiens Human	21621	12,1298	3983	1941	1343	794	948	742	94	70	191	1,19	0,78	0,94
P07355	ANXA2_HUMAN	Annexin A2 Annexin II Lipocortin II Calpactin I heavy chain Chromobindin 8 p36 Protein I	38579	7,784	28581	25318	16360	11865	11393	10973	1120	577	627	0,96	0,96	0,92
P62888	RL30_HUMAN	60S ribosomal protein L30 Homo sapiens Human	12775	9,9531	1400	1053	553	305	473	280		26	138	1,55	0,59	0,92
P35241	RADI_HUMAN	Radixin Homo sapiens Human	68521	5,9687	774	660	286	848	1078	779	296	459	126	1,27	0,72	0,92
P50454	SERPH_HUMAN	Serpin H1 precursor Collagen binding protein Colligin 47 kDa heat shock protein Rheumatoid ar	46411	9,0436	4255	4242	1039	2465	3412	2251	253	525	679	1,38	0,66	0,91
P04899	GNAI2_HUMAN	Guanine nucleotide binding protein G i alpha 2 subunit Adenylate cyclase inhibiting G alpha prote	40425	5,1899	5488	5586	4692	2380	3109	2168	293	267	515	1,31	0,70	0,91
P00761	TRYP_PIG	Trypsin precursor	24393	6,9139	13948	7113	6278	14146	12579	12877	4253	4669	3726	0,89	1,02	0,91
P20339	RAB5A_HUMAN	Ras related protein Rab 5A Homo sapiens Human	23643	8,2504	2507	3173	766	637	456	577	178	162	423	0,72	1,27	0,91
P78371	TCPB_HUMAN	T complex protein 1 subunit beta TCP 1 beta CCT beta Homo sapiens Human	57452	5,9903	3094	837	673	2462	1681	2169	97	178	203	0,68	1,29	0,88
P07339	CATD_HUMAN	Cathepsin D precursor EC 3 4 23 5 Contains Cathepsin D light chain Cathepsin D heavy chain H	44523	6,0903	2737	635	683	2352	1855	2068	177	210	499	0,79	1,11	0,88
P40227	TCPZ_HUMAN	T complex protein 1 subunit zeta TCP 1 zeta CCT zeta CCT zeta 1 Tcp20 HTR3 Acute morphine	57987	6,2238	1164	584	448	1581	1185	1385	223	236	46	0,75	1,17	0,88
Q14247	SRC8_HUMAN	Src substrate cortactin Amplaxin Oncogene EMS1 Homo sapiens Human	61598	5,0759	499	157	132	937	1364	820	102	577		1,46	0,60	0,88
Q99832	TCPH_HUMAN	T complex protein 1 subunit eta TCP 1 eta CCT eta HIV 1 Nef interacting protein Homo sapiens	59328	7,519	1407	522	731	1635	1605	1411	122	915	97	0,98	0,88	0,86
P32004	L1CAM_HUMAN	Neural cell adhesion molecule L1 precursor N CAM L1 CD171 antigen Homo sapiens Human	139915	5,7935	1860	1112	789	8450	9166	7287	288	703	671	1,08	0,79	0,86
P53985	MOT1_HUMAN	Monocarboxylate transporter 1 MCT 1 Solute carrier family 16 member 1 Homo sapiens Human	53922	8,7162	2080	1835	770	1998	2244	1715	275	426	348	1,12	0,76	0,86
P38646	GRP75_HUMAN	Stress 70 protein mitochondrial precursor 75 kDa glucose regulated protein GRP 75 Peptide bind	73634	5,7795	1130	382	669	2805	1331	2363	196	256	585	0,47	1,78	0,84
P49368	TCPG_HUMAN	T complex protein 1 subunit gamma TCP 1 gamma CCT gamma hTRiC5 Homo sapiens Human	60495	6,0584	2369	1266	482	1858	1334	1564	263	246	179	0,72	1,17	0,84
P67936	TPM4_HUMAN	Tropomyosin alpha 4 chain Tropomyosin 4 TM30p1 Homo sapiens Human	28504	4,4705	347	568	252	390	1189	322	248			3,05	0,27	0,82
Q95604	1C17_HUMAN	HLA class I histocompatibility antigen Cw 17 alpha chain precursor MHC class I antigen Cw 17 Ho	41212	6,3448	2569	4411	3550	1022	2115	837		136	155	2,07	0,40	0,82
P05556	ITB1_HUMAN	Integrin beta 1 precursor Fibronectin receptor subunit beta Integrin VLA 4 subunit beta CD29 an	88406	5,1361	3220	3181	1987	8573	10139	6946	753	1351	460	1,18	0,69	0,81
P50991	TCPD_HUMAN	T complex protein 1 subunit delta TCP 1 delta CCT delta Stimulator of TAR RNA binding Homo s	57887	7,7825	2986	700	709	2352	1676	1888	188	186	145	0,71	1,13	0,80
P68104	EF1A1_HUMAN	Elongation factor 1 alpha 1 EF 1 alpha 1 Elongation factor 1 A 1 eEF1A 1 Elongation factor Tu	50109	9,3417	1475	914	605	2654	2694	2120	600	402	339	1,01	0,79	0,80
P07196	NFL_HUMAN	Neurofilament light polypeptide NF L Neurofilament triplet L protein 68 kDa neurofilament prote	61479	4,4323	99		218	1102		870	1064		115	0,00		0,79
P04406	G3P_HUMAN	Glyceraldehyde 3 phosphate dehydrogenase EC 1 2 1 12 GAPDH Homo sapiens Human	36030	8,6966	12969	3540	3890	10768	6504	8375	451	800	2166	0,60	1,29	0,78
O00767	ACOD_HUMAN	Acyl CoA desaturase EC 1 14 19 1 Stearoyl CoA desaturase Fatty acid desaturase Delta 9 desat	41496	9,206	551		213	894		693	68			0,00		0,78
P84095	RHOG_HUMAN	Rho related GTP binding protein RhoG precursor Homo sapiens Human	21294	8,0839	335	3419	2394	867	759	654		239	311	0,88	0,86	0,75
P23634	AT2B4_HUMAN	Plasma membrane calcium transporting ATPase 4 EC 3 6 3 8 PMCA4 Plasma membrane calcium pump iso	137832	6,1584	409	298	74	1451	9206	1087	619			6,35	0,12	0,75
P63092	GNAS2_HUMAN	Guanine nucleotide binding protein G s subunit alpha isoforms short Adenylate cyclase stimulating	45635	5,4659	1348	1736	1063	1315	1467	916		723	148	1,12	0,62	0,70
P14136	GFAP_HUMAN	Glial fibrillary acidic protein astrocyte GFAP Homo sapiens Human	49849	5,2546	432		437	741		510			348	0,00		0,69
P61019	RAB2A_HUMAN	Ras related protein Rab 2A Homo sapiens Human	23530	6,08	3037	6144	823	757	1153	507	33	194		1,52	0,44	0,67
Q16720	AT2B3_HUMAN	Plasma membrane calcium transporting ATPase 3 EC 3 6 3 8 PMCA3 Plasma membrane calcium pump iso	134112	5,2954	247	236	162	2796	1035	1867	1122		600	0,37	1,80	0,67
Q9NQC3	RTN4_HUMAN	Reticulon 4 Neurite outgrowth inhibitor Nogo protein Foocen Neuroendocrine specific protein	129851	4,2241	893	224	582	6815	2601	4524	894	590	1243	0,38	1,74	0,66
P16070	CD44_HUMAN	CD44 antigen precursor Phagocytic glycoprotein I PGP 1 HUTCH I Extracellular matrix receptor	81503	4,9845	3074	1449	834	10009	8331	6297	736	1654	811	0,83	0,76	0,63
P39656	OST48_HUMAN	Dolichyl diphosphooligosaccharide protein glycosyltransferase 48 kDa subunit precursor EC 2 4 1 11	48779	5,304	851	969	354	1304	1838	803	448	363	91	1,41	0,44	0,62
P35613	BASI_HUMAN	Basigin precursor Leukocyte activation antigen M6 Collagenase stimulatory factor Extracellular	42174	5,2813	11563	5566	2793	8496	5948	5148	309	378	582	0,70	0,87	0,61
P48960	CD97_HUMAN	CD97 antigen precursor Leukocyte antigen CD97 Homo sapiens Human	91809	6,4658	812	327	246	3821	2131	2309	1432	584		0,56	1,08	0,60
P09104	ENOG_HUMAN	Gamma enolase EC 4 2 1 11 2 phospho D glycerate hydro lyase Neural enolase Neuron specific en	47239	4,7177	761	759	641	919	765	552		65		0,83	0,72	0,60
P02794	FRIH_HUMAN	Ferritin heavy chain EC 1 16 3 1 Ferritin H subunit Proliferation inducing gene 15 protein H	21212	5,187	1961	252	349	1238	1421	651	255			1,15	0,46	0,53
P04259	K2C6B_HUMAN	Keratin type II cytoskeletal 6B Cytokeratin 6B CK 6B K6b keratin Homo sapiens Human	59962	8,0537	906	554	331	447	1121	233		236		2,51	0,21	0,52
P04222	1C03_HUMAN	HLA class I histocompatibility antigen Cw 3 alpha chain precursor MHC class I antigen Cw 3 Homo	40835	5,9418	4499	3925	3082	706	320	191	697			0,45	0,60	0,27
P04264	K2C1_HUMAN	Keratin type II cytoskeletal 1 Cytokeratin 1 CK 1 Keratin 1 K1 67 kDa cytokeratin Hair a	65977	8,2736	5229	5646	592	5661	6765	1075	537	618	130	1,20	0,16	0,19
P35527	K1C9_HUMAN	Keratin type I cytoskeletal 9 Cytokeratin 9 CK 9 Keratin 9 K9 Homo sapiens Human	62091	5,0138	3661	1152	40	3704	1981	471	862	471		0,53	0,24	0,13

Q96DA2	RB39B_HUMAN	Ras related protein Rab 39B Homo sapiens Human	24606	7,8463	2155	3272	629	4561	298	323	1200			0,07	1,08	0,07
P27482	CALL3_HUMAN	Calmodulin like protein 3 Calmodulin related protein NB 1 CaM like protein CLP Homo sapiens	16879	4,0964	1061	500		124	125		77			1,00	0,00	0,00
P24844	MLRN_HUMAN	Myosin regulatory light chain 2 smooth muscle isoform Myosin RLC Myosin regulatory light chain 9	19814	4,591	1403			129			60			0,00		0,00
P10316	1A69_HUMAN	HLA class I histocompatibility antigen A 69 alpha chain MHC class I antigen A 69 Aw 69 A 28	40951	6,4497	7681	6911		138	192		3			1,39	0,00	0,00
P30486	1B48_HUMAN	HLA class I histocompatibility antigen B 48 alpha chain precursor MHC class I antigen B 48 Bw 48	40337	5,5919	5086	4942		206	216		20			1,05	0,00	0,00
P31949	S10AB_HUMAN	Protein S100 A11 S100 calcium binding protein A11 Protein S100C Calgizzarin MLN 70 Homo sa	11732	7,0206	2483	325		240	124		75			0,52	0,00	0,00
P48741	HSP77_HUMAN	Heat shock 70 kDa protein 7 Heat shock 70 kDa protein B Fragment Homo sapiens Human	26889	7,3592	1345	856		242	187		26			0,77	0,00	0,00
O43169	CYB5B_HUMAN	Cytochrome b5 type B precursor Cytochrome b5 outer mitochondrial membrane isoform Homo sapiens	16321	4,6871	1860	241		260	311		31			1,20	0,00	0,00
P09493	TPM1_HUMAN	Tropomyosin 1 alpha chain Alpha tropomyosin Homo sapiens Human	32688	4,489	401	216		265	369		81			1,39	0,00	0,00
O00161	SNP23_HUMAN	Synaptosomal associated protein 23 SNAP 23 Vesicle membrane fusion protein SNAP 23 Homo sapien	23339	4,6963	881	454		284	427		48	276		1,50	0,00	0,00
Q15286	RAB35_HUMAN	Ras related protein Rab 35 Rab 1C GTP binding protein RAY Homo sapiens Human	23010	8,3928	1811	2796		289	911			57		3,15	0,00	0,00
Q02543	RL18A_HUMAN	60S ribosomal protein L18a Homo sapiens Human	20748	11,1048	1545	315		301	347		48			1,15	0,00	0,00
P62330	ARF6_HUMAN	ADP ribosylation factor 6 Homo sapiens Human	20069	9,2919	899	848		330	460		47			1,39	0,00	0,00
O60762	DPM1_HUMAN	Dolichol phosphate mannosyltransferase EC 2 4 1 83 Dolichol phosphate mannose synthase Dolichyl	29615	9,8582	108	725		331	460			120		1,39	0,00	0,00
P13073	COX41_HUMAN	Cytochrome c oxidase subunit 4 isoform 1 mitochondrial precursor EC 1 9 3 1 Cytochrome c oxidase	19564	9,9142	865			341			126			0,00		0,00
P61803	DAD1_HUMAN	Dolichyl diphosphooligosaccharide protein glycosyltransferase subunit DAD1 EC 2 4 1 119 Oligosac	12488	6,917	2794	1214		351	489		121			1,39	0,00	0,00
P68371	TBB2C_HUMAN	Tubulin beta 2C chain Tubulin beta 2 chain Homo sapiens Human	49799	4,602	863	389		359	927		107			2,58	0,00	0,00
P16520	GBB3_HUMAN	Guanine nucleotide binding protein G I G S G T subunit beta 3 Transducin beta chain 3 Homo sa	37196	5,2665	366	283		394	329		146			0,83	0,00	0,00
P10301	RRAS_HUMAN	Ras related protein R Ras precursor p23 Homo sapiens Human	23465	6,5081	824	564		409	340			99		0,83	0,00	0,00
Q9H3N1	TXND1_HUMAN	Thioredoxin domain containing protein 1 precursor Transmembrane Trx related protein Thioredoxin r	31770	4,7208	893	496		443	634		21	167		1,43	0,00	0,00
P08758	ANXA5_HUMAN	Annexin A5 Annexin V Lipocortin V Endonexin II Calphobindin I CBP I Placental anticoagula	35914	4,732	637			482			35			0,00		0,00
P43307	SSRA_HUMAN	Translocon associated protein subunit alpha precursor TRAP alpha Signal sequence receptor subunit	32215	4,1724	676	303		498	728		18			1,46	0,00	0,00
P61158	ARP3_HUMAN	Actin like protein 3 Actin related protein 3 Homo sapiens Human	47341	5,5104	786	139		508	513		80			1,01	0,00	0,00
Q02878	RL6_HUMAN	60S ribosomal protein L6 TAX responsive enhancer element binding protein 107 TAXREB107 Neoplasm	32707	11,024	700			511			306			0,00		0,00
P26373	RL13_HUMAN	60S ribosomal protein L13 Breast basic conserved protein 1 Homo sapiens Human	24246	12,0586	1767			517			66			0,00		0,00
O15173	PGRC2_HUMAN	Membrane associated progesterone receptor component 2 Progesterone membrane binding protein Stero	23803	4,5414	1344	281		526	517		44	97		0,98	0,00	0,00
P29992	GNA11_HUMAN	Guanine nucleotide binding protein subunit alpha 11 G alpha 11 Guanine nucleotide binding protein	42096	5,3677	503	261		532	581		163	67		1,09	0,00	0,00
Q96BM9	ARL8A_HUMAN	ADP ribosylation factor like protein 8A ADP ribosylation factor like protein 10B Novel small G pr	21402	7,829	1061	625		561	240		86	55		0,43	0,00	0,00
Q15738	NSDHL_HUMAN	Sterol 4 alpha carboxylate 3 dehydrogenase decarboxylating EC 1 1 1 170 H105e3 protein Homo s	41873	8,0825	490	224		561	717		65	12		1,28	0,00	0,00
O14828	SCAM3_HUMAN	Secretory carrier associated membrane protein 3 Secretory carrier membrane protein 3 Homo sapien	38294	7,5685	504	742		623	1131		42	245		1,82	0,00	0,00
P11233	RALA_HUMAN	Ras related protein Ral A precursor Homo sapiens Human	23551	6,9847	2270	1882		657	694		148	156		1,06	0,00	0,00
P18124	RL7_HUMAN	60S ribosomal protein L7 Homo sapiens Human	29207	11,0649	1053			660			164			0,00		0,00
P51571	SSRD_HUMAN	Translocon associated protein subunit delta precursor TRAP delta Signal sequence receptor subunit	18986	5,722	1485	1163		669	945		153	241		1,41	0,00	0,00
Q9P0L0	VAPA_HUMAN	Vesicle associated membrane protein associated protein A VAMP associated protein A VAMP A VAP A	27875	8,9002	491	715		685	779		115			1,14	0,00	0,00
Q8TCT9	HM13_HUMAN	Minor histocompatibility antigen H13 EC 3 4 99 Signal peptide peptidase Presenilin like prote	41461	5,9846	1345	225		687	540		120	53		0,79	0,00	0,00
P21291	CSRP1_HUMAN	Cysteine and glycine rich protein 1 Cysteine rich protein 1 CRP1 CRP Homo sapiens Human	20553	8,5529	1332			706			187			0,00		0,00
P37802	TAGL2_HUMAN	Transgelin 2 SM22 alpha homolog Homo sapiens Human	22377	8,4485	3287	1033		737	427		31	182		0,58	0,00	0,00
P51572	BAP31_HUMAN	B cell receptor associated protein 31 BCR associated protein Bap31 p28 Bap31 Protein CDM 6C6	27974	8,9064	449			744			288			0,00		0,00
Q01628	IFM3_HUMAN	Interferon induced transmembrane protein 3 Interferon inducible protein 1 8U Homo sapiens Human	14622	6,5874	4881	1148		763	646		90	60		0,85	0,00	0,00
P09543	CN37_HUMAN	2 3 cyclic nucleotide 3 phosphodiesterase EC 3 1 4 37 CNP CNPase Homo sapiens Human	47548	9,3611	378	625		763	1090		118	205		1,43	0,00	0,00
Q9C075	K1C23_HUMAN	Keratin type I cytoskeletal 23 Cytokeratin 23 CK 23 Keratin 23 K23 Homo sapiens Human	48101	6,0815	511			785			454			0,00		0,00
P20340	RAB6A_HUMAN	Ras related protein Rab 6A Rab 6 Homo sapiens Human	23577	5,2256	1976	1553		805	733		288	159		0,91	0,00	0,00
Q9NVI7	ATD3A_HUMAN	ATPase family AAA domain containing protein 3A Homo sapiens Human	71324	9,286	265			807			187			0,00		0,00
P39023	RL3_HUMAN	60S ribosomal protein L3 HIV 1 TAR RNA binding protein B TARBP B Homo sapiens Human	46079	10,6181	1075	151		845	547		75			0,65	0,00	0,00
P15586	GNS_HUMAN	N acetylglucosamine 6 sulfatase precursor EC 3 1 6 14 G6S Glucosamine 6 sulfatase Homo sapie	62042	8,257	287	250		894	793		165	142		0,89	0,00	0,00
Q6NZI2	PTRF_HUMAN	Polymerase I and transcript release factor Homo sapiens Human	43449	5,339	471	182		954	3590		289			3,76	0,00	0,00
P04075	ALDOA_HUMAN	Fructose bisphosphate aldolase A EC 4 1 2 13 Muscle type aldolase Lung cancer antigen NY LU 1	39395	8,0645	1155	252		997	1077		177			1,08	0,00	0,00
P36578	RL4_HUMAN	60S ribosomal protein L4 L1 Homo sapiens Human	47667	11,495	1100	350		1003	1402		487			1,40	0,00	0,00
P04626	ERBB2_HUMAN	Receptor tyrosine protein kinase erbB 2 precursor EC 2 7 10 1 p185erbB2 C erbB 2 NEU proto on	137821	5,5023	50	30		1083	3215		336			2,97	0,00	0,00
P04083	ANXA1_HUMAN	Annexin A1 Annexin I Lipocortin I Calpactin II Chromobindin 9 p35 Phospholipase A2 inhibi	38689	6,6359	3155	411		1146	636		125			0,55	0,00	0,00
O95782	AP2A1_HUMAN	AP 2 complex subunit alpha 1 Adapter related protein complex 2 alpha 1 subunit Alpha adaptin A	107486	6,6502	175			1200			190			0,00		0,00
P42892	ECE1_HUMAN	Endothelin converting enzyme 1 EC 3 4 24 71 ECE 1 Homo sapiens Human	87108	5,5091	392	339		1313	2752		261	580		2,10	0,00	0,00
P07437	TBB5_HUMAN	Tubulin beta chain Tubulin beta 5 chain Homo sapiens Human	49638	4,5903	1381	158		1342	884		22			0,66	0,00	0,00
Q8IV08	PLD3_HUMAN	Phospholipase D3 EC 3 1 4 4 PLD 3 Choline phosphatase 3 Phosphatidylcholine hydrolyzing phosp	54670	6,0071	498	400		1358	1790		251	283		1,32	0,00	0,00
Q13885	TBB2A_HUMAN	Tubulin beta 2A chain Homo sapiens Human	49874	4,5903	1604	177		1406	945		215			0,67	0,00	0,00
P13667	PDIA4_HUMAN	Protein disulfide isomerase A4 precursor EC 5 3 4 1 Protein ERp 72 ERp72 Homo sapiens Human	72887	4,7677	499	479		1535	1681		338	241		1,09	0,00	0,00
Q15303	ERBB4_HUMAN	Receptor tyrosine protein kinase erbB 4 precursor EC 2 7 10 1 p180erbB4 Tyrosine kinase type ce	146712	5,9447	50			1908			1360			0,00		0,00
P08962	CD63_HUMAN	CD63 antigen Melanoma associated antigen ME491 Ocular melanoma associated antigen OMA81H Gran	25619	7,7239	2966	2294		1957	1439		397	113		0,74	0,00	0,00
O43795	MYO1B_HUMAN	Myosin Ib Myosin I alpha MMI alpha MMIa MYH 1c Homo sapiens Human	131901	9,6751	177	234		1981	4193		101	2679		2,12	0,00	0,00
Q8NFJ5	RAI3_HUMAN	Retinoic acid induced protein 3 G protein coupled receptor family C group 5 member A Retinoic aci	40224	8,144	1389	1705		2007	1928		183	90		0,96	0,00	0,00
P26006	ITA3_HUMAN	Integrin alpha 3 precursor Galactoprotein B3 GAPB3 VLA 3 alpha chain FRP 2 CD49c antigen	118622	6,596	419	310		2059	4519		696	1805		2,19	0,00	0,00
O60716	CTND1_HUMAN	Catenin delta 1 p120 catenin p120 ctn Cadherin associated Src substrate CAS p120 cas H	108103	5,7969	1018	334		2639	2534		33	299		0,96	0,00	0,00
Q9Y490	TLN1_HUMAN	Talin 1 Homo sapiens Human	269596	5,6871	248	121		4828	6524		854	1532		1,35	0,00	0,00
Q12774	ARHG5_HUMAN	Rho guanine nucleotide exchange factor 5 Guanine nucleotide regulatory protein TIM Oncogene TIM	60018	7,5516	320			5054			399			0,00		0,00
P33527	MRP1_HUMAN	Multidrug resistance associated protein 1 ATP binding cassette sub family C member 1 Leukotriene	171450	6,6945	221			5927			3175			0,00		0,00
P30613	KPYR_HUMAN	Pyruvate kinase isozymes R L EC 2 7 1 40 R type L type pyruvate kinase Red cell liver pyruvate	61791	7,6335	278	73		6144	6067		2966			0,99	0,00	0,00
RANDOM9877	RANDOM9877	Random Sequence 9877	36185	5,0228	125	112		7698	20834			4982		2,71	0,00	0,00
P68133	ACTS_HUMAN	Actin alpha skeletal muscle Alpha actin 1 Homo sapiens Human	42023	5,0709	14647	11439		17511	3521		99	292		0,20	0,00	0,00
P63261	ACTG_HUMAN	Actin cytoplasmic 2 Gamma actin Homo sapiens Human	41765	5,1594	34887	29566		81549	90933		9300			1,12	0,00	0,00

MS 2, HeLa

					MW Score			MW ppm			SD ppm					
Accession	Entry	Description	mW (Da)	pI (pH)	NI	4h	7h	NI	4h	7h	NI	4h	7h	NI → 4h	4h → 7h	NI → 7h
P03101.2	VL1_HPV16	HPV16 Major capsid protein L1	59435	7,95		1412	2652		2054	3582		510	593			
Q29718	1B82_HUMAN	HLA class I histocompatibility antigen B 82 alpha chain precursor MHC class I antigen B 82 Homo	40396	5,59		4589	3027		833	3130		377	30			
P21926	CD9_HUMAN	CD9 antigen p24 Leukocyte antigen MIC3 Motility related protein MRP 1 Tetraspanin 29 Tspa	25398	6,90		1718	1435		968	2658		51	2127			
P04062	GLCM_HUMAN	Glucosylceramidase precursor EC 3 2 1 45 Beta glucocerebrosidase Acid beta glucosidase D gluc	59678	7,34		459	499		1493	2273		236	1025			
P30511	HLAF_HUMAN	HLA class I histocompatibility antigen alpha chain F precursor HLA F antigen Leukocyte antigen F	40543	5,24			1085			1673			2089			
P04626	ERBB2_HUMAN	Receptor tyrosine protein kinase erbB 2 precursor EC 2 7 10 1 p185erbB2 C erbB 2 NEU proto on	137821	5,50		102	68		622	1486		333	419			
P25705	ATPA_HUMAN	ATP synthase subunit alpha mitochondrial precursor EC 3 6 3 14 Homo sapiens Human	59713	9,43			625			1156			150			
P07195	LDHB_HUMAN	L lactate dehydrogenase B chain EC 1 1 1 27 LDH B LDH heart subunit LDH H Renal carcinoma a	36615	5,64		2648	1634		646	1133		23	218			
P84085	ARF5_HUMAN	ADP ribosylation factor 5 Homo sapiens Human	20516	6,36			846			951			145			
Q07065	CKAP4_HUMAN	Cytoskeleton associated protein 4 63 kDa membrane protein p63 Homo sapiens Human	65982	5,54			200			872			82			
P16520	GBB3_HUMAN	Guanine nucleotide binding protein G I G S G T subunit beta 3 Transducin beta chain 3 Homo sa	37196	5,27		345	375		402	811		452	343			
Q8NF37	PCAT1_HUMAN	1 acylglycerophosphocholine O acyltransferase 1 EC 2 3 1 23 Lung type acyl coa lysophosphatidylch	59113	5,66			298			777			343			
P36873	PP1G_HUMAN	Serine threonine protein phosphatase PP1 gamma catalytic subunit EC 3 1 3 16 PP 1G Protein phos	36959	6,10		621	344		550	766		167	700			
Q10589	BST2_HUMAN	Bone marrow stromal antigen 2 precursor BST 2 CD317 antigen HM1 24 antigen Homo sapiens Hum	19756	5,29		1318	976		651	692		205	10			
O15144	ARPC2_HUMAN	Actin related protein 2 3 complex subunit 2 ARP2 3 complex 34 kDa subunit p34 ARC Homo sapiens	34311	6,98			523			619			281			
P13646	K1C13_HUMAN	Keratin type I cytoskeletal 13 Cytokeratin 13 CK 13 Keratin 13 K13 Homo sapiens Human	49555	4,71			153			618			608			
P04792	HSPB1_HUMAN	Heat shock protein beta 1 HspB1 Heat shock 27 kDa protein HSP 27 Stress responsive protein 27	22768	5,96		1254	506		419	602		92	137		1,44	
P78386	KRT85_HUMAN	Keratin type II cuticular Hb5 Type II hair keratin Hb5 Keratin 85 K85 K12 12 Homo sapiens	55766	6,20			170			589			361			
Q9BVK6	TMED9_HUMAN	Transmembrane emp24 domain containing protein 9 precursor Glycoprotein 25L2 Homo sapiens Human	25088	6,78		570	1085		438	554		12	10		1,27	
P41219	PERI_HUMAN	Peripherin Homo sapiens Human	53618	5,21			258			512			259			
P84077	ARF1_HUMAN	ADP ribosylation factor 1 Homo sapiens Human	20683	6,37		1905	2813		394	498		482	133		1,26	
P13929	ENOB_HUMAN	Beta enolase EC 4 2 1 11 2 phospho D glycerate hydro lyase Muscle specific enolase MSE Skel	46957	7,66		260	620		394	467		262	8		1,18	
P35900	K1C20_HUMAN	Keratin type I cytoskeletal 20 Cytokeratin 20 CK 20 Keratin 20 K20 Protein IT Homo sapi	48456	5,39			287			467			193			
Q95604	1C17_HUMAN	HLA class I histocompatibility antigen Cw 17 alpha chain precursor MHC class I antigen Cw 17 Ho	41212	6,34			5043			453			315			
P20340	RAB6A_HUMAN	Ras related protein Rab 6A Rab 6 Homo sapiens Human	23577	5,23		620	730		213	427		115	52		2,01	
Q15836	VAMP3_HUMAN	Vesicle associated membrane protein 3 VAMP 3 Synaptobrevin 3 Cellubrevin CEB Homo sapiens	11301	9,41		7666	8851		393	422		89	140		1,07	
P10599	THIO_HUMAN	Thioredoxin Trx ATL derived factor ADF Surface associated sulphydryl protein SASP Homo s	11729	4,62			3411			413			145			
Q9UHA4	MK1I1_HUMAN	Mitogen activated protein kinase kinase 1 interacting protein 1 MEK binding partner 1 Mp1 Homo	13614	7,30			1743			370			42			
P30050	RL12_HUMAN	60S ribosomal protein L12 Homo sapiens Human	17807	9,90			612			345			88			
P61018	RAB4B_HUMAN	Ras related protein Rab 4B Homo sapiens Human	23571	5,70		2101	2295		120	303		71	288		2,52	
Q13636	RAB31_HUMAN	Ras related protein Rab 31 Rab 22B Homo sapiens Human	21554	6,67		567	586		271	298		17	32		1,10	
Q9NSB2	KRT84_HUMAN	Keratin type II cuticular Hb4 Type II hair keratin Hb4 Keratin 84 K84 Homo sapiens Human	64854	7,61		81	128		432	288		185	50		0,67	
P04350	TBB4_HUMAN	Tubulin beta 4 chain Tubulin 5 beta Homo sapiens Human	49553	4,59			542			250			3			
P16188	1A30_HUMAN	HLA class I histocompatibility antigen A 30 alpha chain precursor MHC class I antigen A 30 Homo	40879	5,67		7616	6369		305	235		94	188		0,77	
P20337	RAB3B_HUMAN	Ras related protein Rab 3B Homo sapiens Human	24742	4,65			2092			225			162			
P29777	GNAO2_HUMAN	Guanine nucleotide binding protein G o subunit alpha 2 Homo sapiens Human	40061	5,51			1817			217			48			
P16190	1A33_HUMAN	HLA class I histocompatibility antigen A 33 alpha chain precursor MHC class I antigen A 33 Aw 33	40866	6,23			7483			209			141			

P30447	1A23_HUMAN	HLA class I histocompatibility antigen A 23 alpha chain precursor MHC class I antigen A 23 A 9	40707	5,88			6593			82			7			
Q96E17	RAB3C_HUMAN	Ras related protein Rab 3C Homo sapiens Human	25935	4,90	1753	1815	2195	95	195	561	22	35	20	2,06	2,87	5,92
P13796	PLSL_HUMAN	Plastin 2 L plastin Lymphocyte cytosolic protein 1 LCP 1 LC64P Homo sapiens Human	70244	5,02	190		159	162		764	19		171	0,00		4,71
Q05524	ENO1B_HUMAN	Alpha enolase lung specific EC 4 2 1 11 2 phospho D glycerate hydro lyase Non neural enolase	49446	5,71	497	640	762	625	1309	2923	148	1060	2046	2,09	2,23	4,68
P19087	GNAT2_HUMAN	Guanine nucleotide binding protein G t alpha 2 subunit Transducin alpha 2 chain Homo sapiens	40150	4,93	1110	955	793	246	321	1120	231	18	724	1,30	3,49	4,56
P13637	AT1A3_HUMAN	Sodium potassium transporting ATPase alpha 3 chain EC 3 6 3 9 Sodium pump 3 Na K ATPase 3	111677	5,05	4443	4667	2838	926	1011	3876	256	471	4772	1,09	3,83	4,19
P61020	RAB5B_HUMAN	Ras related protein Rab 5B Homo sapiens Human	23691	8,24	948	600	1215	72	178	292	7	64	26	2,46	1,64	4,04
Q15771	RAB30_HUMAN	Ras related protein Rab 30 Homo sapiens Human	23043	4,71	2730	2551	2455	89	119	352	23	30	360	1,34	2,96	3,96
P11488	GNAT1_HUMAN	Guanine nucleotide binding protein G t alpha 1 subunit Transducin alpha 1 chain Homo sapiens	40015	5,27	1046	929	840	230	382	771	43	336	383	1,66	2,02	3,36
P17066	HSP76_HUMAN	Heat shock 70 kDa protein 6 Heat shock 70 kDa protein B Homo sapiens Human	70984	5,73	810	842	787	821	1563	2729	316	1492	2293	1,90	1,75	3,32
Q96AX2	RAB37_HUMAN	Ras related protein Rab 37 Homo sapiens Human	24799	5,94	2370	1573	1565	215	275	686	141	121	344	1,28	2,50	3,19
P26232	CTNA2_HUMAN	Catenin alpha 2 Alpha catenin related protein Alpha N catenin Homo sapiens Human	105246	5,37	559	210	421	716	1340	2248	243	683	1713	1,87	1,68	3,14
P10316	1A69_HUMAN	HLA class I histocompatibility antigen A 69 alpha chain MHC class I antigen A 69 Aw 69 A 28	40951	6,45	9978	11330	6126	228	1065	688	51	948	895	4,66	0,65	3,01
P20338	RAB4A_HUMAN	Ras related protein Rab 4A Homo sapiens Human	23856	5,72	2560	1793	2286	110	185	323	68	31	175	1,67	1,75	2,92
P35749	MYH11_HUMAN	Myosin 11 Myosin heavy chain 11 Myosin heavy chain smooth muscle isoform SMMHC Homo sapiens	227197	5,25	313	242	297	2220	3365	6358	1100	900	4964	1,52	1,89	2,86
P20339	RAB5A_HUMAN	Ras related protein Rab 5A Homo sapiens Human	23643	8,25	1533	1694	816	194	298	550	59	59	375	1,53	1,84	2,83
Q7Z406	MYH14_HUMAN	Myosin 14 Myosin heavy chain 14 Myosin heavy chain nonmuscle IIc Nonmuscle myosin heavy chain	227861	5,63	173	142	182	2629	3730	7298	1143	1686	1402	1,42	1,96	2,78
O00560	SDCB1_HUMAN	Syntenin 1 Syndecan binding protein 1 Melanoma differentiation associated protein 9 MDA 9 Sca	32423	7,35	2040	3366	2770	1034	1375	2868	153	89	1163	1,33	2,09	2,77
P23634	AT2B4_HUMAN	Plasma membrane calcium transporting ATPase 4 EC 3 6 3 8 PMCA4 Plasma membrane calcium pump iso	137832	6,16	989	872	559	2115	6879	5664	419	6551	3512	3,25	0,82	2,68
Q9HAV0	GBB4_HUMAN	Guanine nucleotide binding protein subunit beta 4 Transducin beta chain 4 Homo sapiens Human	37543	5,53	1997	2164	1623	242	513	629	94	98	168	2,12	1,23	2,60
P51153	RAB13_HUMAN	Ras related protein Rab 13 Cell growth inhibiting gene 4 protein Homo sapiens Human	22759	9,57	926	1725	944	128	261	330	5	97	179	2,04	1,26	2,58
Q9H254	SPTN4_HUMAN	Spectrin beta chain brain 3 Spectrin non erythroid beta chain 3 Beta IV spectrin Homo sapien	288806	5,63	43		64	4337		11113	2189		8995	0,00		2,56
P07197	NFM_HUMAN	Neurofilament medium polypeptide NF M Neurofilament triplet M protein 160 kDa neurofilament pro	102386	4,70	124	99	115	680	335	1735	466	45	927	0,49	5,17	2,55
P62937	PPIA_HUMAN	Peptidyl prolyl cis trans isomerase A EC 5 2 1 8 PPIase A Rotamase A Cyclophilin A Cyclospo	18000	7,85	1727	1195	934	546	666	1382	7	38	206	1,22	2,07	2,53
P68133	ACTS_HUMAN	Actin alpha skeletal muscle Alpha actin 1 Homo sapiens Human	42023	5,07	10198		6883	17274		43016	22872		8681	0,00		2,49
P07196	NFL_HUMAN	Neurofilament light polypeptide NF L Neurofilament triplet L protein 68 kDa neurofilament prote	61479	4,43	284	183	215	348	427	837	118	265	599	1,23	1,96	2,41
P07900	HS90A_HUMAN	Heat shock protein HSP 90 alpha HSP 86 Renal carcinoma antigen NY REN 38 Homo sapiens Human	84606	4,75	613	671	520	1397	1725	3292	881	1038	2690	1,23	1,91	2,36
P13797	PLST_HUMAN	Plastin 3 T plastin Homo sapiens Human	70391	5,39	1859	1402	1264	2067	2314	4780	467	568	3223	1,12	2,07	2,31
Q99808	S29A1_HUMAN	Equilibrative nucleoside transporter 1 Equilibrative nitrobenzylmercaptopurine riboside sensitive n	50186	8,27	558	433	431	545	725	1222	20	50	538	1,33	1,69	2,24
P60033	CD81_HUMAN	CD81 antigen 26 kDa cell surface protein TAPA 1 Target of the antiproliferative antibody 1 Tetr	25792	4,91	1042	1360	2897	535	897	1183	217	48	65	1,68	1,32	2,21
P31946	1433B_HUMAN	14 3 3 protein beta alpha Protein kinase C inhibitor protein 1 KCIP 1 Protein 1054 Homo sapi	28064	4,57	1789	1409	1664	220	277	486	112	118	96	1,26	1,76	2,21
P54652	HSP72_HUMAN	Heat shock related 70 kDa protein 2 Heat shock 70 kDa protein 2 Homo sapiens Human	69977	5,41	3379	3652	2978	617	475	1344	277	165	699	0,77	2,83	2,18
P12036	NFH_HUMAN	Neurofilament heavy polypeptide NF H Neurofilament triplet H protein 200 kDa neurofilament prot	112411	5,76	148	101	100	698	1067	1472	383	546	632	1,53	1,38	2,11
P08670	VIME_HUMAN	Vimentin Homo sapiens Human	53619	4,86	3191	1449	3141	2076	1565	4371	204	224	2466	0,75	2,79	2,10
Q15286	RAB35_HUMAN	Ras related protein Rab 35 Rab 1C GTP binding protein RAY Homo sapiens Human	23010	8,39	3475	3038	2797	432	421	900	400	270	909	0,97	2,14	2,08
P17661	DESM_HUMAN	Desmin Homo sapiens Human	53503	5,03	454	342	369	446	609	916	182	569	684	1,37	1,50	2,05
O60637	TSN3_HUMAN	Tetraspanin 3 Tspan 3 Transmembrane 4 superfamily member 8 Tetraspanin TM4 A Homo sapiens H	27999	5,42	886	544	389	540	800	1085	107	182	302	1,48	1,36	2,01
P68371	TBB2C_HUMAN	Tubulin beta 2C chain Tubulin beta 2 chain Homo sapiens Human	49799	4,60	1255	1660	753	258	202	517	258	103	367	0,78	2,55	2,00
P54707	AT12A_HUMAN	Potassium transporting ATPase alpha chain 2 EC 3 6 3 10 Proton pump Non gastric H K ATPas	115825	6,10	158	181	124	857	2654	1691	704	1933	1432	3,09	0,64	1,97
O75923	DYSF_HUMAN	Dysferlin Dystrophy associated fer 1 like protein Fer 1 like protein 1 Homo sapiens Human	237142	5,30	57	96	71	2205	3183	4330	1599	1380	666	1,44	1,36	1,96
P08779	K1C16_HUMAN	Keratin type I cytoskeletal 16 Cytokeratin 16 CK 16 Keratin 16 K16 Homo sapiens Human	51236	4,79	316		321	241		470	144		439	0,00		1,96
Q13501	SQSTM_HUMAN	p62/Sequestosome 1 Phosphotyrosine independent ligand for the Lck SH2 domain of 62 kDa Ubiquitin bind	47656	4,93	1481	2563	2432	1662	1920	3218	228	286	703	1,16	1,68	1,94
P05787	K2C8_HUMAN	Keratin type II cytoskeletal 8 Cytokeratin 8 CK 8 Keratin 8 K8 Homo sapiens Human	53671	5,34	754	886	953	914	910	1760	156	14	372	1,00	1,93	1,93
P13747	HLAE_HUMAN	HLA class I histocompatibility antigen alpha chain E precursor MHC class I antigen E Homo sapie	40131	5,53	2148	1526	2741	486	597	907	134	326	362	1,23	1,52	1,87
Q8NBS9	TXND5_HUMAN	Thioredoxin domain containing protein 5 precursor Thioredoxin like protein p46 Endoplasmic reticu	47598	5,56	738	372	639	826	428	1524	199	34	1002	0,52	3,56	1,84
Q6IAA8	CK059_HUMAN	UPF0404 protein C11orf59 Homo sapiens Human	17733	4,83	3618	3163	4377	499	620	914	205	63	412	1,24	1,47	1,83
P23229	ITA6_HUMAN	Integrin alpha 6 precursor VLA 6 CD49f antigen Contains Integrin alpha 6 heavy chain Integrin	126539	6,37	680	616	2466	2933	3789	5359	412	1528	1034	1,29	1,41	1,83
P62158	CALM_HUMAN	Calmodulin CaM Homo sapiens Human	16826	3,88	7158	8888	9380	981	1159	1763	357	98	1255	1,18	1,52	1,80
Q9H0U4	RAB1B_HUMAN	Ras related protein Rab 1B Homo sapiens Human	22157	5,39	6435	6365	7356	657	816	1171	161	366	557	1,24	1,44	1,78
P61006	RAB8A_HUMAN	Ras related protein Rab 8A Oncogene c mel Homo sapiens Human	23653	9,43	5028	4323	3525	438	510	769	102	139	606	1,17	1,51	1,76
P01893	HLAH_HUMAN	HLA class I histocompatibility antigen alpha chain H precursor HLA AR HLA 12 4 Homo sapiens	40824	5,87	7183	5531	4534	722	1612	1241	419	436	578	2,23	0,77	1,72
P11142	HSP7C_HUMAN	Heat shock cognate 71 kDa protein Heat shock 70 kDa protein 8 Homo sapiens Human	70854	5,20	5975	5515	6500	3397	3752	5755	424	428	1328	1,10	1,53	1,69
Q16352	AINX_HUMAN	Alpha internexin Alpha Inx 66 kDa neurofilament protein Neurofilament 66 NF 66 Homo sapien	55357	5,17	255	163	350	205	295	348	96	9	21	1,43	1,18	1,69
P35579	MYH9_HUMAN	Myosin 9 Myosin heavy chain 9 Myosin heavy chain nonmuscle IIa Nonmuscle myosin heavy chain II	226390	5,34	1830	1429	1209	7837	7523	13145	408	963	5361	0,96	1,75	1,68
O75369	FLNB_HUMAN	Filamin B FLN B Beta filamin Actin binding like protein Thyroid autoantigen Truncated actin	278018	5,37	345	311	280	6160	5617	10325	2461	2214	3111	0,91	1,84	1,68
P38405	GNAL_HUMAN	Guanine nucleotide binding protein G olf subunit alpha Adenylate cyclase stimulating G alpha prote	44280	6,22	796	860	804	471	623	787	328	390	430	1,32	1,26	1,67
P08727	K1C19_HUMAN	Keratin type I cytoskeletal 19 Cytokeratin 19 CK 19 Keratin 19 K19 Homo sapiens Human	44065	4,86	368	286	350	200	260	328	88	187	107	1,30	1,26	1,65
Q9NY65	TBA8_HUMAN	Tubulin alpha 8 chain Alpha tubulin 8 Homo sapiens Human	50061	4,76	970	540	650	956	505	1571	362	8	499	0,53	3,11	1,64
Q13813	SPTA2_HUMAN	Spectrin alpha chain brain Spectrin non erythroid alpha chain Alpha II spectrin Fodrin alpha	284362	5,05	1821	2039	763	8879	11871	14489	2894	2566	4395	1,34	1,22	1,63
P62258	1433E_HUMAN	14 3 3 protein epsilon 14 3 3E Homo sapiens Human	29155	4,44	4738	4626	3586	767	875	1241	65	156	357	1,14	1,42	1,62
O43854	EDIL3_HUMAN	EGF like repeat and discoidin I like domain containing protein 3 precursor EGF like repeats and dis	53729	6,99	1258	1796	1095	1862	2258	2987	364	580	458	1,21	1,32	1,60
Q13733	AT1A4_HUMAN	Sodium potassium transporting ATPase alpha 4 chain EC 3 6 3 9 Sodium pump 4 Na K ATPase 4	114093	6,20	807	879	384	1675	1245	2683	1252	467	2365	0,74	2,16	1,60
Q14247	SRC8_HUMAN	Src substrate cortactin Amplaxin Oncogene EMS1 Homo sapiens Human	61598	5,08	352	370	387	525	581	841	109	98	175	1,11	1,45	1,60
P13473	LAMP2_HUMAN	Lysosome associated membrane glycoprotein 2 precursor LAMP 2 CD107b antigen Homo sapiens Huma	44932	5,23	2563	2143	1753	2900	3598	4583	398	1119	3477	1,24	1,27	1,58
P68366	TBA1_HUMAN	Tubulin alpha 1 chain Alpha tubulin 1 Testis specific alpha tubulin Tubulin H2 alpha Homo sa	49892	4,75	1843	1135	1267	1039	1174	1634	428	443	550	1,13	1,39	1,57
Q14315	FLNC_HUMAN	Filamin C Gamma filamin Filamin 2 Protein FLNc Actin binding like protein ABP L ABP 280 l	290775	5,60	122	97	104	3847	5075	5983	1701	1861	3165	1,32	1,18	1,56
P07737	PROF1_HUMAN	Profilin 1 Profilin I Homo sapiens Human	15044	8,46	4757	2737	2501	611	778	941	60	141	288	1,27	1,21	1,54
Q01814	AT2B2_HUMAN	Plasma membrane calcium transporting ATPase 2 EC 3 6 3 8 PMCA2 Plasma membrane calcium pump iso	136789	5,55	394	314	190	1699	2073	2616	2062	129	1429	1,22	1,26	1,54
O43504	XIP_HUMAN	Hepatitis B virus X interacting protein HBX interacting protein HBV X interacting protein Homo	9607	4,50	2391	3770	4433	329	388	504	54	69	50	1,18	1,30	1,53
P20648	ATP4A_HUMAN	Potassium transporting ATPase alpha chain 1 EC 3 6 3 10 Proton pump Gastric H K ATPase su	114017	5,45	510	499	294	1133	1007	1724	382	420	794	0,89	1,71	1,52
P62745	RHOB_HUMAN	Rho related GTP binding protein RhoB precursor H6 Homo sapiens Human	22109	4,91	2594	3170	2732	227	251	345	76	19	84	1,10	1,37	1,52
Q14254	FLOT2_HUMAN	Flotillin 2 Epidermal surface antigen ESA Homo sapiens Human	41659	5,04	1048	665	671	699	729	1060	217	190	344	1,04	1,45	1,52
P08238	HS90B_HUMAN	Heat shock protein HSP 90 beta HSP 84 HSP 90 Homo sapiens Human	83212	4,77	1718	2127	1636	2071	2340	3125	398	472	500	1,13	1,34	1,51
Q9BSJ8	FA62A_HUMAN	Protein FAM62A Membrane bound C2 domain containing protein Homo sapiens Human	122780	5,46	417	309	569	1412	1628	2127	92	220	827	1,15	1,31	1,51
P46940	IQGA1_HUMAN	Ras GTPase activating like protein IQGAP1 p195 Homo sapiens Human	189132	6,04	1224	1204	812	8231	9167	12361	1093	1255	6184	1,11	1,35	1,50
P61769	B2MG_HUMAN	Beta 2 microglobulin precursor Contains Beta 2 microglobulin variant pI 5 3 Homo sapiens Human	13705	6,07	17287	19364	13161	1381	1635	2069	109	306	1030	1,18	1,27	1,50
P25106	CXCR7_HUMAN	C X C chemokine receptor type 7 CXC R7 CXCR 7 G protein coupled receptor RDC1 homolog RDC 1	41465	7,37	338		351	310		464	122		245	0,00		1,50
P53396	ACLY_HUMAN	ATP citrate synthase EC 2 3 3 8 ATP citrate pro S lyase Citrate cleavage enzyme Homo sapi	120762	6,95	419	337	279	1237	1408	1852	124	261	590	1,14	1,32	1,50
P27348	1433T_HUMAN	14 3 3 protein theta 14 3 3 protein tau 14 3 3 protein T cell HS1 protein Homo sapiens Huma	27746	4,48	1392	1736	1659	286	304	428	90	72	137	1,06	1,41	1,49
Q02878	RL6_HUMAN	60S ribosomal protein L6 TAX responsive enhancer element binding protein 107 TAXREB107 Neoplasm	32707	11,02	405		494	284		422	108		193	0,00		1,49
P04844	RIB2_HUMAN	Dolichyl diphosphooligosaccharide protein glycosyltransferase 63 kDa subunit precursor EC 2 4 1 11	69241	5,33	2763	1898	1550	1954	1943	2772	165	328	1187	0,99	1,43	1,42
P63104	1433Z_HUMAN	14 3 3 protein zeta delta Protein kinase C inhibitor protein 1 KCIP 1 Homo sapiens Human	27727	4,53	4511	4592	4379	774	884	1090	93	87	353	1,14	1,23	1,41
P00761	TRYP_PIG	Trypsin precursor	24393	6,91	10210	11728	8756	14313	20403	20082	3187	1937	2136	1,43	0,98	1,40
Q04695	K1C17_HUMAN	Keratin type I cytoskeletal 17 Cytokeratin 17 CK 17 Keratin 17 K17 39 1 Homo sapiens H	48076	4,78	1640	944	1521	870	482	1214	266	113	265	0,55	2,52	1,40
P05387	RLA2_HUMAN	60S acidic ribosomal protein P2 Renal carcinoma antigen NY REN 44 Homo sapiens Human	11657	4,18	6817	5700	4936	450	400	624	123	72	197	0,89	1,56	1,39
P07237	PDIA1_HUMAN	Protein disulfide isomerase precursor EC 5 3 4 1 PDI Prolyl 4 hydroxylase subunit beta Cellul	57080	4,56	6419	6616	4687	2942	3315	4042	276	573	841	1,13	1,22	1,37
Q96BM9	ARL8A_HUMAN	ADP ribosylation factor like protein 8A ADP ribosylation factor like protein 10B Novel small G pr	21402	7,83	1832	1313	1101	702	971	965	36	207	523	1,38	0,99	1,37
P35241	RADI_HUMAN	Radixin Homo sapiens Human	68521	5,97	1230	1280	865	1002	1139	1376	115	251	637	1,14	1,21	1,37
Q14108	SCRB2_HUMAN	Lysosome membrane protein 2 Lysosome membrane protein II LIMP II Scavenger receptor class B mem	54255	4,82	1555	1646	1046	1684	2126	2306	784	372	936	1,26	1,08	1,37
P62988	UBIQ_HUMAN	Ubiquitin Homo sapiens Human	8559	7,67	29646	34492	34680	2421	2569	3307	581	430	621	1,06	1,29	1,37
P07602	SAP_HUMAN	Proactivator polypeptide precursor Contains Saposin A Protein A Saposin B Val Saposin B Sphin	58073	4,89	5809	4526	5255	8130	9173	11086	663	2067	2504	1,13	1,21	1,36
Q9Y490	TLN1_HUMAN	Talin 1 Homo sapiens Human	269596	5,69	376	322	229	8097	7990	11028	5311	4994	6639	0,99	1,38	1,36
P61019	RAB2A_HUMAN	Ras related protein Rab 2A Homo sapiens Human	23530	6,08	2314	2898	1104	448	655	608	145	82	195	1,46	0,93	1,36
P61981	1433G_HUMAN	14 3 3 protein gamma Protein kinase C inhibitor protein 1 KCIP 1 Homo sapiens Human	28284	4,61	1421	1688	1466	317	256	429	58	97	121	0,81	1,68	1,35
P10809	CH60_HUMAN	60 kDa heat shock protein mitochondrial precursor Hsp60 60 kDa chaperonin CPN60 Heat shock p	61016	5,55	2432	1273	1737	1940	1087	2607	415	90	505	0,56	2,40	1,34
P08107	HSP71_HUMAN	Heat shock 70 kDa protein 1 HSP70 1 HSP70 1 HSP70 2 Homo sapiens Human	69995	5,32	2150	2296	2104	1355	1411	1819	493	188	176	1,04	1,29	1,34
P15311	EZRI_HUMAN	Ezrin p81 Cytovillin Villin 2 Homo sapiens Human	69369	5,87	3059	3235	2997	3753	4499	4954	574	813	1016	1,20	1,10	1,32
P29992	GNA11_HUMAN	Guanine nucleotide binding protein subunit alpha 11 G alpha 11 Guanine nucleotide binding protein	42096	5,37	976	716	369	607	660	792	62	179	250	1,09	1,20	1,30
P39656	OST48_HUMAN	Dolichyl diphosphooligosaccharide protein glycosyltransferase 48 kDa subunit precursor EC 2 4 1 11	48779	5,30	786	528	620	1056	1113	1375	323	350	378	1,05	1,23	1,30
P30101	PDIA3_HUMAN	Protein disulfide isomerase A3 precursor EC 5 3 4 1 Disulfide isomerase ER 60 ERp60 58 kDa mi	56746	5,93	6725	7157	5092	5024	5153	6517	399	607	725	1,03	1,26	1,30
P21333	FLNA_HUMAN	Filamin A Alpha filamin Filamin 1 Endothelial actin binding protein Actin binding protein 280	280561	5,64	3662	2726	2514	18404	20450	23868	2131	5975	6438	1,11	1,17	1,30
P11166	GTR1_HUMAN	Solute carrier family 2 facilitated glucose transporter member 1 Glucose transporter type 1 eryth	54048	8,86	2360	1815	1731	5879	5958	7490	1166	879	225	1,01	1,26	1,27
O00592	PODXL_HUMAN	Podocalyxin like protein 1 precursor Homo sapiens Human	55561	5,23	3508	3067	2186	3817	4386	4854	626	889	334	1,15	1,11	1,27
P19022	CADH2_HUMAN	Cadherin 2 precursor Neural cadherin N cadherin CD325 antigen CDw325 Homo sapiens Human	99747	4,44	1478	1049	942	4572	4820	5791	1191	508	646	1,05	1,20	1,27
P61225	RAP2B_HUMAN	Ras related protein Rap 2b precursor Homo sapiens Human	20491	4,53	1196	859	729	230	201	290	21	38	5	0,87	1,45	1,26
P50454	SERPH_HUMAN	Serpin H1 precursor Collagen binding protein Colligin 47 kDa heat shock protein Rheumatoid ar	46411	9,04	3903	2534	2099	2177	2153	2740	377	249	397	0,99	1,27	1,26
P61421	VA0D_HUMAN	Vacuolar ATP synthase subunit d EC 3 6 3 14 V ATPase d subunit Vacuolar proton pump subunit d	40303	4,70	1750	1981	901	1055	1393	1325	81	425	360	1,32	0,95	1,26
O14828	SCAM3_HUMAN	Secretory carrier associated membrane protein 3 Secretory carrier membrane protein 3 Homo sapien	38294	7,57	709	605	530	550	544	689	36	67	177	0,99	1,27	1,25
Q06830	PRDX1_HUMAN	Peroxiredoxin 1 EC 1 11 1 15 Thioredoxin peroxidase 2 Thioredoxin dependent peroxide reductase	22096	8,24	2971	3049	2444	884	990	1107	269	57	118	1,12	1,12	1,25
P40227	TCPZ_HUMAN	T complex protein 1 subunit zeta TCP 1 zeta CCT zeta CCT zeta 1 Tcp20 HTR3 Acute morphine	57987	6,22	3963	3487	2431	1929	2213	2413	378	550	958	1,15	1,09	1,25
P11279	LAMP1_HUMAN	Lysosome associated membrane glycoprotein 1 precursor LAMP 1 CD107a antigen Homo sapiens Huma	44744	9,27	3996	4183	3778	5999	6281	7497	1101	290	706	1,05	1,19	1,25
P02533	K1C14_HUMAN	Keratin type I cytoskeletal 14 Cytokeratin 14 CK 14 Keratin 14 K14 Homo sapiens Human	51589	4,90	638	901	617	436	701	544	349	248	314	1,61	0,78	1,25

P16435	NCPR_HUMAN	NADPH cytochrome P450 reductase EC 1 6 2 4 CPR P450R Homo sapiens Human	76641	5,24	438		254	848		1056	75		364	0,00		1,25
P00338	LDHA_HUMAN	L lactate dehydrogenase A chain EC 1 1 1 27 LDH A LDH muscle subunit LDH M Proliferation in	36665	8,37	3468	2579	3984	971	819	1200	49	284	259	0,84	1,47	1,24
O75955	FLOT1_HUMAN	Flotillin 1 Homo sapiens Human	47325	7,30	1018	920	907	918	1085	1131	142	299	134	1,18	1,04	1,23
P61224	RAP1B_HUMAN	Ras related protein Rap 1b precursor GTP binding protein smg p21B Homo sapiens Human	20811	5,46	11413	11395	10679	1790	2236	2204	375	595	421	1,25	0,99	1,23
P21796	VDAC1_HUMAN	Voltage dependent anion selective channel protein 1 VDAC 1 hVDAC1 Outer mitochondrial membrane	30753	8,87	1660	1619	1033	1230	1301	1514	126	144	337	1,06	1,16	1,23
Q10471	GALT2_HUMAN	Polypeptide N acetylgalactosaminyltransferase 2 EC 2 4 1 41 Protein UDP acetylgalactosaminyltrans	64691	8,32	838	722	530	1648	1511	2025	405	171	391	0,92	1,34	1,23
P10620	MGST1_HUMAN	Microsomal glutathione S transferase 1 EC 2 5 1 18 Microsomal GST 1 Microsomal GST I Homo sa	17587	9,64	2867	2829	2812	880	995	1077	67	141	268	1,13	1,08	1,22
P06756	ITAV_HUMAN	Integrin alpha V precursor Vitronectin receptor subunit alpha CD51 antigen Contains Integrin a	115964	5,31	1903	2144	1167	5275	5638	6407	1057	912	2915	1,07	1,14	1,21
P04083	ANXA1_HUMAN	Annexin A1 Annexin I Lipocortin I Calpactin II Chromobindin 9 p35 Phospholipase A2 inhibi	38689	6,64	359	895	419	447	466	542	199	39	136	1,04	1,16	1,21
O43707	ACTN4_HUMAN	Alpha actinin 4 Non muscle alpha actinin 4 F actin cross linking protein Homo sapiens Human	104788	5,12	8023	6676	5304	8054	8430	9732	926	1030	2249	1,05	1,15	1,21
P23284	PPIB_HUMAN	Peptidyl prolyl cis trans isomerase B precursor EC 5 2 1 8 PPIase Rotamase Cyclophilin B S	22728	9,76	5762	5763	5919	2287	2045	2726	351	152	501	0,89	1,33	1,19
P35221	CTNA1_HUMAN	Catenin alpha 1 Cadherin associated protein Alpha E catenin NY REN 13 antigen Homo sapiens	100008	5,88	5442	4655	2596	4976	5530	5932	621	1122	2010	1,11	1,07	1,19
P05388	RLA0_HUMAN	60S acidic ribosomal protein P0 L10E Homo sapiens Human	34251	5,60	2263	1141	1633	886	944	1056	189	303	102	1,06	1,12	1,19
P60174	TPIS_HUMAN	Triosephosphate isomerase EC 5 3 1 1 TIM Triose phosphate isomerase Homo sapiens Human	26652	6,50	1644	477	791	489	422	583	99	62	108	0,86	1,38	1,19
Q14697	GANAB_HUMAN	Neutral alpha glucosidase AB precursor EC 3 2 1 84 Glucosidase II subunit alpha Homo sapiens	106806	5,69	898	836	595	3577	4077	4259	664	439	694	1,14	1,04	1,19
P60660	MYL6_HUMAN	Myosin light polypeptide 6 Smooth muscle and nonmuscle myosin light chain alkali 6 Myosin light c	16919	4,36	4008	4641	2851	652	626	776	122	131	170	0,96	1,24	1,19
Q9H4G4	GAPR1_HUMAN	Golgi associated plant pathogenesis related protein 1 Golgi associated PR 1 protein GAPR 1 Glio	17207	9,76	2598	3002	2586	271	331	322	80	107	57	1,22	0,97	1,19
P63096	GNAI1_HUMAN	Guanine nucleotide binding protein G i alpha 1 subunit Adenylate cyclase inhibiting G alpha prote	40335	5,59	3397	3596	2440	640	675	759	159	88	96	1,05	1,12	1,19
P08729	K2C7_HUMAN	Keratin type II cytoskeletal 7 Cytokeratin 7 CK 7 Keratin 7 K7 Sarcolectin Homo sapiens	51386	5,31	1159	759	968	913	666	1072	124	252	192	0,73	1,61	1,17
Q15582	BGH3_HUMAN	Transforming growth factor beta induced protein ig h3 precursor Beta ig h3 Kerato epithelin RGD	74634	7,52	589	448	293	1028	1121	1202	164	299	273	1,09	1,07	1,17
P20645	MPRD_HUMAN	Cation dependent mannose 6 phosphate receptor precursor CD Man 6 P receptor CD MPR 46 kDa manno	30973	5,47	1483	765	1114	606	577	705	30	67	63	0,95	1,22	1,16
Q15084	PDIA6_HUMAN	Protein disulfide isomerase A6 precursor EC 5 3 4 1 Protein disulfide isomerase P5 Thioredoxin	48091	4,76	3895	3076	2125	2266	2153	2623	142	188	721	0,95	1,22	1,16
P20336	RAB3A_HUMAN	Ras related protein Rab 3A Homo sapiens Human	24968	4,66	2758		2573	127		147	30		61	0,00		1,16
P11021	GRP78_HUMAN	78 kDa glucose regulated protein precursor GRP 78 Heat shock 70 kDa protein 5 Immunoglobulin he	72288	4,87	13528	11065	10402	10512	10145	12140	1532	923	2497	0,97	1,20	1,15
P48509	CD151_HUMAN	CD151 antigen Platelet endothelial tetraspan antigen 3 PETA 3 GP27 Membrane glycoprotein SFA	28276	7,30	764	889	769	1159	1239	1335	499	160	422	1,07	1,08	1,15
Q96AC1	PKHC1_HUMAN	Pleckstrin homology domain containing family C member 1 Kindlin 2 Mitogen inducible gene 2 protei	77810	6,25	1085	1058	464	2089	2523	2400	690	319	1268	1,21	0,95	1,15
P14625	ENPL_HUMAN	Endoplasmin precursor Heat shock protein 90 kDa beta member 1 94 kDa glucose regulated protein	92411	4,56	5588	4239	3942	6610	5784	7575	1027	833	1322	0,87	1,31	1,15
P15328	FOLR1_HUMAN	Folate receptor alpha precursor FR alpha Folate receptor 1 Folate receptor adult Adult folat	29799	7,86	5401	5046	3677	4488	4994	5126	316	282	904	1,11	1,03	1,14
Q08043	ACTN3_HUMAN	Alpha actinin 3 Alpha actinin skeletal muscle isoform 3 F actin cross linking protein Homo sap	103229	5,25	781	913	978	1082	1087	1235	1204	712	763	1,00	1,14	1,14
Q12797	ASPH_HUMAN	Aspartyl asparaginyl beta hydroxylase EC 1 14 11 16 Aspartate beta hydroxylase ASP beta hydroxy	85809	4,73	565	314	368	1485	2089	1695	193	1218	174	1,41	0,81	1,14
Q92526	TCPW_HUMAN	T complex protein 1 subunit zeta 2 TCP 1 zeta 2 CCT zeta 2 TCP 1 zeta like CCT zeta like Te	57728	6,65	110	192	176	550	450	625	467	108	36	0,82	1,39	1,14
P35580	MYH10_HUMAN	Myosin 10 Myosin heavy chain 10 Myosin heavy chain nonmuscle IIb Nonmuscle myosin heavy chain	228796	5,27	260	186	304	3644	2374	4134	2268	820	1097	0,65	1,74	1,13
P14923	PLAK_HUMAN	Junction plakoglobin Desmoplakin 3 Desmoplakin III Homo sapiens Human	81577	5,92	5576	5785	3100	5303	5551	6007	964	366	814	1,05	1,08	1,13
Q96AG4	LRC59_HUMAN	Leucine rich repeat containing protein 59 Homo sapiens Human	34908	10,03	1656	1678	1034	1540	1363	1744	378	205	644	0,89	1,28	1,13
Q13162	PRDX4_HUMAN	Peroxiredoxin 4 EC 1 11 1 15 Prx IV Thioredoxin peroxidase AO372 Thioredoxin dependent peroxi	30520	5,83	845	987	831	465	470	525	216	91	345	1,01	1,12	1,13
P62820	RAB1A_HUMAN	Ras related protein Rab 1A YPT1 related protein Homo sapiens Human	22663	5,85	6036	5396	5476	796	701	897	364	208	579	0,88	1,28	1,13
Q15738	NSDHL_HUMAN	Sterol 4 alpha carboxylate 3 dehydrogenase decarboxylating EC 1 1 1 170 H105e3 protein Homo s	41873	8,08	694	296	251	607	444	683	108	173	87	0,73	1,54	1,13
P35222	CTNB1_HUMAN	Catenin beta 1 Beta catenin Homo sapiens Human	85442	5,45	1343	1594	952	2450	2717	2727	292	275	595	1,11	1,00	1,11
P54709	AT1B3_HUMAN	Sodium potassium transporting ATPase subunit beta 3 Sodium potassium dependent ATPase beta 3 subuni	31492	8,44	2107	1695	1656	1499	1408	1662	250	453	276	0,94	1,18	1,11
P21281	VATB2_HUMAN	Vacuolar ATP synthase subunit B brain isoform EC 3 6 3 14 V ATPase B2 subunit Vacuolar proton	56464	5,45	452	364	538	1212	1248	1335	265	367	263	1,03	1,07	1,10
O15427	MOT4_HUMAN	Monocarboxylate transporter 4 MCT 4 MCT 3 Solute carrier family 16 member 3 Homo sapiens Hu	49436	7,89	1981	2047	1366	3167	4678	3471	1101	780	625	1,48	0,74	1,10
Q9UBI6	GBG12_HUMAN	Guanine nucleotide binding protein G I G S G O gamma 12 subunit precursor Homo sapiens Human	8001	9,48	24291	22943	20148	1089	1258	1193	116	190	167	1,16	0,95	1,10
P51149	RAB7A_HUMAN	Ras related protein Rab 7a Homo sapiens Human	23474	6,57	18248	16074	14014	2392	2373	2615	342	206	362	0,99	1,10	1,09
P02786	TFR1_HUMAN	Transferrin receptor protein 1 TfR1 TR TfR Trfr CD71 antigen T9 p90 Homo sapiens Hu	84818	6,16	5926	5871	3721	8658	9227	9415	624	996	780	1,07	1,02	1,09
P48960	CD97_HUMAN	CD97 antigen precursor Leukocyte antigen CD97 Homo sapiens Human	91809	6,47	1464	1307	646	3073	3320	3317	378	531	494	1,08	1,00	1,08
P26038	MOES_HUMAN	Moesin Membrane organizing extension spike protein Homo sapiens Human	67777	6,01	5291	6179	2959	7549	9799	8095	1036	2105	2832	1,30	0,83	1,07
Q15758	AAAT_HUMAN	Neutral amino acid transporter B 0 ATB 0 Solute carrier family 1 member 5 Sodium dependent ne	56562	5,17	8012	7708	5385	8561	8025	9091	2375	2239	2746	0,94	1,13	1,06
P61106	RAB14_HUMAN	Ras related protein Rab 14 Homo sapiens Human	23881	5,80	10077	8097	7870	1630	1560	1702	188	237	387	0,96	1,09	1,04
P68104	EF1A1_HUMAN	Elongation factor 1 alpha 1 EF 1 alpha 1 Elongation factor 1 A 1 eEF1A 1 Elongation factor Tu	50109	9,34	2699	2704	2554	3995	4041	4162	313	259	466	1,01	1,03	1,04
P08174	DAF_HUMAN	Complement decay accelerating factor precursor CD55 antigen Homo sapiens Human	41373	7,46	2029	2857	1387	3481	3963	3626	788	331	882	1,14	0,92	1,04
P61026	RAB10_HUMAN	Ras related protein Rab 10 Homo sapiens Human	22526	8,57	6420	6451	5734	1022	1249	1061	249	154	265	1,22	0,85	1,04
P23528	COF1_HUMAN	Cofilin 1 Cofilin non muscle isoform 18 kDa phosphoprotein p18 Homo sapiens Human	18490	8,22	9299	8628	6232	1103	963	1141	275	128	146	0,87	1,19	1,03
P07099	HYEP_HUMAN	Epoxide hydrolase 1 EC 3 3 2 9 Microsomal epoxide hydrolase Epoxide hydratase Homo sapiens	52914	6,84	1715	1137	1314	1752	1394	1812	503	210	750	0,80	1,30	1,03
P49368	TCPG_HUMAN	T complex protein 1 subunit gamma TCP 1 gamma CCT gamma hTRiC5 Homo sapiens Human	60495	6,06	2109	1998	1152	2383	2727	2452	451	289	482	1,14	0,90	1,03
P32004	L1CAM_HUMAN	Neural cell adhesion molecule L1 precursor N CAM L1 CD171 antigen Homo sapiens Human	139915	5,79	1732	1519	857	8635	8688	8859	1375	1193	1652	1,01	1,02	1,03
P53985	MOT1_HUMAN	Monocarboxylate transporter 1 MCT 1 Solute carrier family 16 member 1 Homo sapiens Human	53922	8,72	2108	2139	1251	2310	2347	2356	299	596	762	1,02	1,00	1,02
Q03135	CAV1_HUMAN	Caveolin 1 Homo sapiens Human	20458	5,59	5315	3193	3308	893	731	907	129	84	175	0,82	1,24	1,02
P68363	TBAK_HUMAN	Tubulin alpha ubiquitous chain Alpha tubulin ubiquitous Tubulin K alpha 1 Homo sapiens Human	50119	4,76	4035	3039	3458	1872	1317	1894	481	725	1173	0,70	1,44	1,01
P09972	ALDOC_HUMAN	Fructose bisphosphate aldolase C EC 4 1 2 13 Brain type aldolase Homo sapiens Human	39431	6,43	1700	1908	810	648	443	655	546	201	61	0,68	1,48	1,01
P01111	RASN_HUMAN	GTPase NRas precursor Transforming protein N Ras Homo sapiens Human	21215	4,82	2029	3349	1167	727	598	729	161	356	143	0,82	1,22	1,00
Q14126	DSG2_HUMAN	Desmoglein 2 precursor HDGC Homo sapiens Human	122308	4,98	1401	1285	714	6593	5456	6604	1845	784	1657	0,83	1,21	1,00
P01891	1A68_HUMAN	HLA class I histocompatibility antigen A 68 alpha chain precursor MHC class I antigen A 68 Aw 68	40883	6,23	11643	10571	8130	3912	3819	3911	2355	895	433	0,98	1,02	1,00
Q9NZM1	MYOF_HUMAN	Myoferlin Fer 1 like protein 3 Homo sapiens Human	234558	5,76	2181	1915	1101	11929	11516	11904	548	1054	1454	0,97	1,03	1,00
P05556	ITB1_HUMAN	Integrin beta 1 precursor Fibronectin receptor subunit beta Integrin VLA 4 subunit beta CD29 an	88406	5,14	5620	5074	3103	9666	10923	9640	1482	423	1461	1,13	0,88	1,00
Q5JWF2	GNAS1_HUMAN	Guanine nucleotide binding protein G s subunit alpha isoforms XLas Adenylate cyclase stimulating G	110955	4,72	1083		666	3506		3479	522		187	0,00		0,99
P60709	ACTB_HUMAN	Actin cytoplasmic 1 Beta actin Homo sapiens Human	41709	5,14	22983	21044	20769	98283	84050	97172	15243	7868	7689	0,86	1,16	0,99
P27105	STOM_HUMAN	Erythrocyte band 7 integral membrane protein Stomatin Protein 7 2b Homo sapiens Human	31710	7,99	21214	26185	15110	6690	7582	6569	541	687	574	1,13	0,87	0,98
P62873	GBB1_HUMAN	Guanine nucleotide binding protein G I G S G T subunit beta 1 Transducin beta chain 1 Homo sa	37353	5,54	8508	9128	6690	3034	2968	2966	350	275	445	0,98	1,00	0,98
P48643	TCPE_HUMAN	T complex protein 1 subunit epsilon TCP 1 epsilon CCT epsilon Homo sapiens Human	59632	5,30	832	1012	441	1638	1333	1599	461	262	151	0,81	1,20	0,98
P16070	CD44_HUMAN	CD44 antigen precursor Phagocytic glycoprotein I PGP 1 HUTCH I Extracellular matrix receptor	81503	4,98	3607	3652	1955	12367	12129	12054	1070	2393	1866	0,98	0,99	0,97
P61586	RHOA_HUMAN	Transforming protein RhoA precursor H12 Homo sapiens Human	21754	5,73	9717	11080	7500	1298	1185	1265	287	286	334	0,91	1,07	0,97
P62834	RAP1A_HUMAN	Ras related protein Rap 1A precursor GTP binding protein smg p21A Ras related protein Krev 1 C2	20973	6,53	5009	4275	4722	564	529	549	173	297	61	0,94	1,04	0,97
P50990	TCPQ_HUMAN	T complex protein 1 subunit theta TCP 1 theta CCT theta Renal carcinoma antigen NY REN 15 Ho	59582	5,27	2215	2624	1499	2535	2328	2460	342	148	257	0,92	1,06	0,97
P38606	VATA_HUMAN	Vacuolar ATP synthase catalytic subunit A EC 3 6 3 14 V ATPase subunit A Vacuolar proton pump a	68260	5,18	1067	790	517	1727	1578	1675	960	506	849	0,91	1,06	0,97
P62879	GBB2_HUMAN	Guanine nucleotide binding protein G I G S G T subunit beta 2 Transducin beta chain 2 G protei	37307	5,54	7158	8847	5566	4119	4467	3993	474	353	425	1,08	0,89	0,97
P04843	RIB1_HUMAN	Dolichyl diphosphooligosaccharide protein glycosyltransferase 67 kDa subunit precursor EC 2 4 1 11	68526	5,93	2087	1544	889	2430	2377	2343	398	704	217	0,98	0,99	0,96
Q09666	AHNK_HUMAN	Neuroblast differentiation associated protein AHNAK Desmoyokin Fragments Homo sapiens Human	312292	6,26	2124	2157	1081	34176	33559	32841	4575	3516	9618	0,98	0,98	0,96
P12814	ACTN1_HUMAN	Alpha actinin 1 Alpha actinin cytoskeletal isoform Non muscle alpha actinin 1 F actin cross lin	102992	5,09	2793	2333	2240	2703	2754	2592	525	664	1129	1,02	0,94	0,96
P13987	CD59_HUMAN	CD59 glycoprotein precursor Membrane attack complex inhibition factor MACIF MAC inhibitory prot	14167	6,03	11068	10741	10063	3364	3694	3217	380	737	649	1,10	0,87	0,96
P08195	4F2_HUMAN	4F2 cell surface antigen heavy chain 4F2hc Lymphocyte activation antigen 4F2 large subunit 4F2	57908	5,03	27178	29180	22737	31755	30599	30295	4988	2626	5920	0,96	0,99	0,95
P14618	KPYM_HUMAN	Pyruvate kinase isozymes M1 M2 EC 2 7 1 40 Pyruvate kinase muscle isozyme Pyruvate kinase 2 3	57900	7,75	9619	7556	5336	7902	5735	7537	1145	315	1517	0,73	1,31	0,95
P00533	EGFR_HUMAN	Epidermal growth factor receptor precursor EC 2 7 10 1 Receptor tyrosine protein kinase ErbB 1	134190	6,23	960	960	437	4856	5676	4628	564	585	955	1,17	0,82	0,95
P30499	1C01_HUMAN	HLA class I histocompatibility antigen Cw 1 alpha chain precursor MHC class I antigen Cw 1 Homo	40939	5,37	7450	8706	5412	3401	3573	3228	1116	1125	1944	1,05	0,90	0,95
Q99832	TCPH_HUMAN	T complex protein 1 subunit eta TCP 1 eta CCT eta HIV 1 Nef interacting protein Homo sapiens	59328	7,52	1339	1645	503	1878	2315	1779	796	598	1173	1,23	0,77	0,95
P04406	G3P_HUMAN	Glyceraldehyde 3 phosphate dehydrogenase EC 1 2 1 12 GAPDH Homo sapiens Human	36030	8,70	23558	21549	13536	29564	34948	27551	7941	6881	6472	1,18	0,79	0,93
P06733	ENOA_HUMAN	Alpha enolase EC 4 2 1 11 2 phospho D glycerate hydro lyase Non neural enolase NNE Enolase	47139	7,17	1786	1243	1352	1942	2090	1807	712	522	367	1,08	0,86	0,93
Q9NVJ2	ARL8B_HUMAN	ADP ribosylation factor like protein 8B ADP ribosylation factor like protein 10C Novel small G pr	21525	8,60	1136		1119	942		877	177		49	0,00		0,93
Q13740	CD166_HUMAN	CD166 antigen precursor Activated leukocyte cell adhesion molecule ALCAM Homo sapiens Human	65061	5,85	2411	2041	1554	3648	3902	3368	271	328	707	1,07	0,86	0,92
P05023	AT1A1_HUMAN	Sodium potassium transporting ATPase alpha 1 chain precursor EC 3 6 3 9 Sodium pump 1 Na K	112824	5,17	7460	7215	5061	15847	14752	14628	2098	1945	1925	0,93	0,99	0,92
P50991	TCPD_HUMAN	T complex protein 1 subunit delta TCP 1 delta CCT delta Stimulator of TAR RNA binding Homo s	57887	7,78	2669	3150	1282	2815	2547	2580	228	208	434	0,90	1,01	0,92
P27797	CALR_HUMAN	Calreticulin precursor CRP55 Calregulin HACBP ERp60 grp60 Homo sapiens Human	48111	4,09	8138	6329	5444	8064	5656	7307	738	1339	1858	0,70	1,29	0,91
Q6YHK3	CD109_HUMAN	CD109 antigen precursor p180 150 KDa TGF beta 1 binding protein r150 Platelet specific Gov an	161586	5,48	607	518	316	3890	4055	3517	1136	906	243	1,04	0,87	0,90
P27824	CALX_HUMAN	Calnexin precursor Major histocompatibility complex class I antigen binding protein p88 p90 IP9	67525	4,27	3349	3090	2459	9751	7810	8812	766	1284	1392	0,80	1,13	0,90
O00159	MYO1C_HUMAN	Myosin Ic Myosin I beta MMI beta MMIb Homo sapiens Human	117965	9,74	1531	1508	1009	5354	5717	4814	715	1297	647	1,07	0,84	0,90
P07437	TBB5_HUMAN	Tubulin beta chain Tubulin beta 5 chain Homo sapiens Human	49638	4,59	1789	1986	1006	1432	1663	1275	253	577	751	1,16	0,77	0,89
P07355	ANXA2_HUMAN	Annexin A2 Annexin II Lipocortin II Calpactin I heavy chain Chromobindin 8 p36 Protein I	38579	7,78	44769	43890	31234	16038	18056	14272	2642	1379	2648	1,13	0,79	0,89
Q01650	LAT1_HUMAN	Large neutral amino acids transporter small subunit 1 L type amino acid transporter 1 4F2 light c	54974	7,63	8854	7631	6881	13576	12404	11989	784	2852	2587	0,91	0,97	0,88
P29966	MARCS_HUMAN	Myristoylated alanine rich C kinase substrate MARCKS Protein kinase C substrate 80 kDa protein	31535	4,26	4475	7380	4505	2028	2496	1777	758	735	634	1,23	0,71	0,88
P35613	BASI_HUMAN	Basigin precursor Leukocyte activation antigen M6 Collagenase stimulatory factor Extracellular	42174	5,28	12537	10927	4984	6787	6746	5943	797	651	879	0,99	0,88	0,88
P05026	AT1B1_HUMAN	Sodium potassium transporting ATPase subunit beta 1 Sodium potassium dependent ATPase beta 1 subuni	35038	8,65	2350	1597	1352	1738	1778	1512	280	391	278	1,02	0,85	0,87
P17987	TCPA_HUMAN	T complex protein 1 subunit alpha TCP 1 alpha CCT alpha Homo sapiens Human	60305	5,71	1655	1831	502	1774	1780	1529	136	240	213	1,00	0,86	0,86
P08648	ITA5_HUMAN	Integrin alpha 5 precursor Fibronectin receptor subunit alpha Integrin alpha F VLA 5 CD49e an	114464	5,39	2059	1859	924	10026	10073	8628	1233	832	1699	1,00	0,86	0,86
P19012	K1C15_HUMAN	Keratin type I cytoskeletal 15 Cytokeratin 15 CK 15 Keratin 15 K15 Homo sapiens Human	49137	4,51	304	294	362	245	637	211	92	144	51	2,60	0,33	0,86
P04899	GNAI2_HUMAN	Guanine nucleotide binding protein G i alpha 2 subunit Adenylate cyclase inhibiting G alpha prote	40425	5,19	12969	12828	6961	3676	3684	3157	567	463	437	1,00	0,86	0,86
Q15907	RB11B_HUMAN	Ras related protein Rab 11B GTP binding protein YPT3 Homo sapiens Human	24473	5,55	4306	3376	2281	1060	807	904	373	77	282	0,76	1,12	0,85
O60716	CTND1_HUMAN	Catenin delta 1 p120 catenin p120 ctn Cadherin associated Src substrate CAS p120 cas H	108103	5,80	1444	1220	679	4186	3444	3542	1587	245	558	0,82	1,03	0,85
P11233	RALA_HUMAN	Ras related protein Ral A precursor Homo sapiens Human	23551	6,98	2541	2721	1085	774	728	655	27	83	44	0,94	0,90	0,85
P08754	GNAI3_HUMAN	Guanine nucleotide binding protein G k subunit alpha G i alpha 3 Homo sapiens Human	40506	5,36	7948	7972	5500	2789	2762	2333	500	349	310	0,99	0,84	0,84
P26006	ITA3_HUMAN	Integrin alpha 3 precursor Galactoprotein B3 GAPB3 VLA 3 alpha chain FRP 2 CD49c antigen	118622	6,60	1027	806	413	4883	4943	4054	414	905	1554	1,01	0,82	0,83
Q16720	AT2B3_HUMAN	Plasma membrane calcium transporting ATPase 3 EC 3 6 3 8 PMCA3 Plasma membrane calcium pump iso	134112	5,30	629	702	302	1712	1662	1419	1134	1128	1269	0,97	0,85	0,83
P04075	ALDOA_HUMAN	Fructose bisphosphate aldolase A EC 4 1 2 13 Muscle type aldolase Lung cancer antigen NY LU 1	39395	8,06	14659	17323	9685	8818	9086	7298	1176	458	869	1,03	0,80	0,83
P78371	TCPB_HUMAN	T complex protein 1 subunit beta TCP 1 beta CCT beta Homo sapiens Human	57452	5,99	2086	2469	1049	2631	2450	2165	359	394	838	0,93	0,88	0,82

O43175	SERA_HUMAN	D 3 phosphoglycerate dehydrogenase EC 1 1 1 95 3 PGDH Homo sapiens Human	56614	6,28	3165	2957	1826	2457	2363	2009	259	141	518	0,96	0,85	0,82
P80723	BASP_HUMAN	Brain acid soluble protein 1 BASP1 protein Neuronal axonal membrane protein NAP 22 22 kDa neuro	22680	4,42	77045	79310	57688	15942	17985	12988	2807	6674	4066	1,13	0,72	0,81
Q9Y4G6	TLN2_HUMAN	Talin 2 Homo sapiens Human	271382	5,27	65	84	74	10235	23132	7998	6439	34351	1001	2,26	0,35	0,78
P20020	AT2B1_HUMAN	Plasma membrane calcium transporting ATPase 1 EC 3 6 3 8 PMCA1 Plasma membrane calcium pump iso	138667	5,64	1108	1012	500	3860	3686	2942	1293	1143	1310	0,95	0,80	0,76
P09471	GNAO1_HUMAN	Guanine nucleotide binding protein G o subunit alpha 1 Homo sapiens Human	40024	5,19	1641	1407	1232	605	305	442	782	117	51	0,50	1,45	0,73
P84095	RHOG_HUMAN	Rho related GTP binding protein RhoG precursor Homo sapiens Human	21294	8,08	4447	3410	1893	430	362	312	188	31	79	0,84	0,86	0,73
P51148	RAB5C_HUMAN	Ras related protein Rab 5C RAB5L L1880 Homo sapiens Human	23467	8,58	2083	2500	1321	841	692	608	171	102	193	0,82	0,88	0,72
P50993	AT1A2_HUMAN	Sodium potassium transporting ATPase alpha 2 chain precursor EC 3 6 3 9 Sodium pump 2 Na K	112193	5,32	3697	3747	2064	1539	1570	1099	723	915	322	1,02	0,70	0,71
P63000	RAC1_HUMAN	Ras related C3 botulinum toxin substrate 1 precursor p21 Rac1 Ras like protein TC25 Cell migrat	21436	8,57	4583	4597	2905	1383	1697	960	313	209	448	1,23	0,57	0,69
P43121	MUC18_HUMAN	Cell surface glycoprotein MUC18 precursor Melanoma associated antigen MUC18 Melanoma cell adhesio	71562	5,45	1783	2174	787	3459	3404	2244	335	172	473	0,98	0,66	0,65
Q13443	ADAM9_HUMAN	ADAM 9 precursor EC 3 4 24 A disintegrin and metalloproteinase domain 9 Metalloprotease disin	90497	7,31	376	251	219	1562	1148	913	956	313	147	0,74	0,79	0,58
P11717	MPRI_HUMAN	Cation independent mannose 6 phosphate receptor precursor CI Man 6 P receptor CI MPR M6PR Ins	274097	5,52	449	360	229	15211	9059	8553	9219	3669	3301	0,60	0,94	0,56
P34931	HS70L_HUMAN	Heat shock 70 kDa protein 1L Heat shock 70 kDa protein 1 like Heat shock 70 kDa protein 1 Hom H	70331	5,65	2499	2956	2501	1599	967	752	2344	602	380	0,60	0,78	0,47
P68032	ACTC_HUMAN	Actin alpha cardiac muscle 1 Alpha cardiac actin Homo sapiens Human	41991	5,07	14827	12508	11885	6633	23189	2536	767	13881	82	3,50	0,11	0,38
P35609	ACTN2_HUMAN	Alpha actinin 2 Alpha actinin skeletal muscle isoform 2 F actin cross linking protein Homo sap	103788	5,15	1062	665	973	3786	1759	1391	3219	618	1198	0,46	0,79	0,37
Q6QNY1	BL1S2_HUMAN	Biogenesis of lysosome related organelles complex 1 subunit 2 BLOC subunit 2 Centrosome associate	15951	4,58	863	561	652	2313	2343	601	1546	1790	594	1,01	0,26	0,26
Q96DA2	RB39B_HUMAN	Ras related protein Rab 39B Homo sapiens Human	24606	7,85	2591	1748	2411	748	247	186	952	136	133	0,33	0,75	0,25
P54764	EPHA4_HUMAN	Ephrin type A receptor 4 precursor EC 2 7 10 1 Tyrosine protein kinase receptor SEK Receptor pr	109789	6,17	164	231		521	2099		267	1350		4,03	0,00	0,00
P04264	K2C1_HUMAN	Keratin type II cytoskeletal 1 Cytokeratin 1 CK 1 Keratin 1 K1 67 kDa cytokeratin Hair a	65977	8,27	2483	5794		2536	8878		412	542		3,50	0,00	0,00
P35527	K1C9_HUMAN	Keratin type I cytoskeletal 9 Cytokeratin 9 CK 9 Keratin 9 K9 Homo sapiens Human	62091	5,01	920	3425		1254	4262		162	429		3,40	0,00	0,00
P30613	KPYR_HUMAN	Pyruvate kinase isozymes R L EC 2 7 1 40 R type L type pyruvate kinase Red cell liver pyruvate	61791	7,63	471	259		195	654		47	495		3,36	0,00	0,00
P08134	RHOC_HUMAN	Rho related GTP binding protein RhoC precursor H9 Homo sapiens Human	21992	6,19	4145	5082		163	476		56	380		2,92	0,00	0,00
P35908	K22E_HUMAN	Keratin type II cytoskeletal 2 epidermal Cytokeratin 2e K2e CK 2e Keratin 2 Homo sapiens	65825	8,05	330	1429		531	1552		275	213		2,92	0,00	0,00
P31947	1433S_HUMAN	14 3 3 protein sigma Stratifin Epithelial cell marker protein 1 Homo sapiens Human	27756	4,48	665	632		78	215		12	142		2,76	0,00	0,00
Q13509	TBB3_HUMAN	Tubulin beta 3 chain Tubulin beta III Tubulin beta 4 Homo sapiens Human	50400	4,64	1067	1187		309	679		111	867		2,20	0,00	0,00
P29323	EPHB2_HUMAN	Ephrin type B receptor 2 precursor EC 2 7 10 1 Tyrosine protein kinase receptor EPH 3 DRT Rec	117416	6,11	155	120		446	966		72	472		2,17	0,00	0,00
Q12907	LMAN2_HUMAN	Vesicular integral membrane protein VIP36 precursor GP36b glycoprotein Lectin mannose binding 2	40203	6,49	341	279		9652	17786		13188	3081		1,84	0,00	0,00
O60488	ACSL4_HUMAN	Long chain fatty acid CoA ligase 4 EC 6 2 1 3 Long chain acyl CoA synthetase 4 LACS 4 Homo	79136	8,37	248	277		521	872		20	379		1,67	0,00	0,00
P60953	CDC42_HUMAN	Cell division control protein 42 homolog precursor G25K GTP binding protein Homo sapiens Human	21296	5,67	1827	1453		177	274		5	42		1,55	0,00	0,00
P02794	FRIH_HUMAN	Ferritin heavy chain EC 1 16 3 1 Ferritin H subunit Proliferation inducing gene 15 protein H	21212	5,19	1274	1196		341	526		114	162		1,54	0,00	0,00
Q6EMK4	VASN_HUMAN	Vasorin precursor Protein Slit like 2 Homo sapiens Human	71667	7,09	462	610		1189	1775		36	1006		1,49	0,00	0,00
Q8TCT9	HM13_HUMAN	Minor histocompatibility antigen H13 EC 3 4 99 Signal peptide peptidase Presenilin like prote	41461	5,98	282	430		290	428		131	47		1,48	0,00	0,00
P10909	CLUS_HUMAN	Clusterin precursor Complement associated protein SP 40 40 Complement cytolysis inhibitor CLI	52461	5,84	898	656		896	1209		437	94		1,35	0,00	0,00
P14314	GLU2B_HUMAN	Glucosidase 2 subunit beta precursor Glucosidase II subunit beta Protein kinase C substrate 60 1	59387	4,13	808	905		1334	1718		525	359		1,29	0,00	0,00
P04259	K2C6B_HUMAN	Keratin type II cytoskeletal 6B Cytokeratin 6B CK 6B K6b keratin Homo sapiens Human	59962	8,05	82	517		630	802		188	328		1,27	0,00	0,00
P55283	CADH4_HUMAN	Cadherin 4 precursor Retinal cadherin R cadherin R CAD Homo sapiens Human	100217	4,46	165	133		610	770		241	451		1,26	0,00	0,00
Q93084	AT2A3_HUMAN	Sarcoplasmic endoplasmic reticulum calcium ATPase 3 EC 3 6 3 8 Calcium pump 3 SERCA3 SR Ca 2	113904	5,26	173	167		1039	1285		452	716		1,24	0,00	0,00
Q9P2B2	FPRP_HUMAN	Prostaglandin F2 receptor negative regulator precursor Prostaglandin F2 alpha receptor regulatory p	98494	6,15	359	403		2079	2502		693	628		1,20	0,00	0,00
P08962	CD63_HUMAN	CD63 antigen Melanoma associated antigen ME491 Ocular melanoma associated antigen OMA81H Gran	25619	7,72	2648	3572		2017	2414		104	745		1,20	0,00	0,00
P17813	EGLN_HUMAN	Endoglin precursor CD105 antigen Homo sapiens Human	70533	6,14	512	1136		1438	1698		544	469		1,18	0,00	0,00
Q01628	IFM3_HUMAN	Interferon induced transmembrane protein 3 Interferon inducible protein 1 8U Homo sapiens Human	14622	6,59	2995	3523		878	1027		90	192		1,17	0,00	0,00
P02769	ALBU_BOVIN	Serum albumin precursor Allergen Bos d 6 BSA	69248	5,76	280	327		780	884		105	260		1,13	0,00	0,00
O43657	TSN6_HUMAN	Tetraspanin 6 Tspan 6 Transmembrane 4 superfamily member 6 T245 protein Tetraspanin TM4 D A	27545	8,06	1581	1195		428	476		19	15		1,11	0,00	0,00
Q16658	FSCN1_HUMAN	Fascin Singed like protein 55 kDa actin bundling protein p55 Homo sapiens Human	54496	6,88	612	546		1011	1111		307	213		1,10	0,00	0,00
Q5ZPR3	CD276_HUMAN	CD276 antigen precursor Costimulatory molecule B7 homolog 3 B7 H3 4Ig B7 H3 Homo sapiens	57199	4,58	704	601		1263	1365		249	190		1,08	0,00	0,00
O43491	E41L2_HUMAN	Band 4 1 like protein 2 Generally expressed protein 4 1 4 1G Homo sapiens Human	112519	5,18	309	447		1776	1917		546	118		1,08	0,00	0,00
P33527	MRP1_HUMAN	Multidrug resistance associated protein 1 ATP binding cassette sub family C member 1 Leukotriene	171450	6,69	288	246		2595	2778		345	597		1,07	0,00	0,00
P09543	CN37_HUMAN	2 3 cyclic nucleotide 3 phosphodiesterase EC 3 1 4 37 CNP CNPase Homo sapiens Human	47548	9,36	698	825		1106	1165		87	226		1,05	0,00	0,00
P49006	MRP_HUMAN	MARCKS related protein MARCKS like protein 1 Macrophage myristoylated alanine rich C kinase subst	19517	4,45	920	1143		255	268		99	47		1,05	0,00	0,00
Q9NP58	ABCB6_HUMAN	Mitochondrial ATP binding cassette sub family B member 6 Mitochondrial ABC transporter 3 Mt ABC t	93826	8,50	320	428		1570	1646		203	152		1,05	0,00	0,00
Q05682	CALD1_HUMAN	Caldesmon CDM Homo sapiens Human	93194	5,45	347	324		1332	1373		231	316		1,03	0,00	0,00
Q14651	PLSI_HUMAN	Plastin 1 I plastin Intestine specific plastin Homo sapiens Human	70308	5,17	248	105		707	723		244	568		1,02	0,00	0,00
Q8NFJ5	RAI3_HUMAN	Retinoic acid induced protein 3 G protein coupled receptor family C group 5 member A Retinoic aci	40224	8,14	1849	1456		2284	2286		915	361		1,00	0,00	0,00
Q07020	RL18_HUMAN	60S ribosomal protein L18 Homo sapiens Human	21621	12,13	858	1409		416	416		65	17		1,00	0,00	0,00
P04222	1C03_HUMAN	HLA class I histocompatibility antigen Cw 3 alpha chain precursor MHC class I antigen Cw 3 Homo	40835	5,94	7642	8388		332	327		112	262		0,98	0,00	0,00
Q9H5V8	CDCP1_HUMAN	CUB domain containing protein 1 precursor Transmembrane and associated with src kinases Membrane	92815	7,99	549	581		1637	1603		81	86		0,98	0,00	0,00
Q9Y2Q5	MAPIP_HUMAN	Mitogen activated protein binding protein interacting protein Late endosomal lysosomal Mp1 interact	13498	5,11	2242	2381		273	266		57	58		0,98	0,00	0,00
P16615	AT2A2_HUMAN	Sarcoplasmic endoplasmic reticulum calcium ATPase 2 EC 3 6 3 8 Calcium pump 2 SERCA2 SR Ca 2	114682	5,05	678	488		2790	2688		691	582		0,96	0,00	0,00
P42892	ECE1_HUMAN	Endothelin converting enzyme 1 EC 3 4 24 71 ECE 1 Homo sapiens Human	87108	5,51	474	475		2773	2639		2060	698		0,95	0,00	0,00
P07339	CATD_HUMAN	Cathepsin D precursor EC 3 4 23 5 Contains Cathepsin D light chain Cathepsin D heavy chain H	44523	6,09	916	987		1080	1022		140	191		0,95	0,00	0,00
P63092	GNAS2_HUMAN	Guanine nucleotide binding protein G s subunit alpha isoforms short Adenylate cyclase stimulating	45635	5,47	4277	2672		1600	1494		98	89		0,93	0,00	0,00
P32119	PRDX2_HUMAN	Peroxiredoxin 2 EC 1 11 1 15 Thioredoxin peroxidase 1 Thioredoxin dependent peroxide reductase	21878	5,57	860	868		196	178		108	95		0,91	0,00	0,00
P78310	CXAR_HUMAN	Coxsackievirus and adenovirus receptor precursor Coxsackievirus B adenovirus receptor hCAR CVB3	40004	7,43	1103	730		452	408		77	14		0,90	0,00	0,00
P17693	HLAG_HUMAN	HLA class I histocompatibility antigen alpha chain G precursor HLA G antigen Homo sapiens Huma	38199	5,34	1358	816		524	461		430	336		0,88	0,00	0,00
P63261	ACTG_HUMAN	Actin cytoplasmic 2 Gamma actin Homo sapiens Human	41765	5,16	26593	34741		97662	83520		18471	13298		0,86	0,00	0,00
P36578	RL4_HUMAN	60S ribosomal protein L4 L1 Homo sapiens Human	47667	11,50	283	270		772	637		257	229		0,83	0,00	0,00
P37802	TAGL2_HUMAN	Transgelin 2 SM22 alpha homolog Homo sapiens Human	22377	8,45	1288	764		457	344		90	33		0,75	0,00	0,00
P29317	EPHA2_HUMAN	Ephrin type A receptor 2 precursor EC 2 7 10 1 Tyrosine protein kinase receptor ECK Epithelial	108184	5,74	551	426		2350	1752		1145	554		0,75	0,00	0,00
O43852	CALU_HUMAN	Calumenin precursor Crocalbin IEF SSP 9302 Homo sapiens Human	37083	4,28	1888	1764		1272	938		78	227		0,74	0,00	0,00
P54753	EPHB3_HUMAN	Ephrin type B receptor 3 precursor EC 2 7 10 1 Tyrosine protein kinase receptor HEK 2 Homo sap	110259	5,89	167	89		736	539		460	302		0,73	0,00	0,00
P00387	NCB5R_HUMAN	NADH cytochrome b5 reductase EC 1 6 2 2 B5R Diaphorase 1 Cytochrome b5 reductase 3 Contains	34212	7,39	621	458		1058	758		480	117		0,72	0,00	0,00
P61158	ARP3_HUMAN	Actin like protein 3 Actin related protein 3 Homo sapiens Human	47341	5,51	648	542		863	607		361	241		0,70	0,00	0,00
O14983	AT2A1_HUMAN	Sarcoplasmic endoplasmic reticulum calcium ATPase 1 EC 3 6 3 8 Calcium pump 1 SERCA1 SR Ca 2	110181	4,88	196	126		574	404		182	125		0,70	0,00	0,00
P48552	NRIP1_HUMAN	Nuclear receptor interacting protein 1 Nuclear factor RIP140 Receptor interacting protein 140	126863	8,14	85	27		1183	825		943	7		0,70	0,00	0,00
P61604	CH10_HUMAN	10 kDa heat shock protein mitochondrial Hsp10 10 kDa chaperonin CPN10 Early pregnancy factor	10924	9,47	1673	1038		227	150		37	30		0,66	0,00	0,00
O15031	PLXB2_HUMAN	Plexin B2 precursor MM1 Homo sapiens Human	204996	5,80	310	382		6276	4048		3624	1305		0,65	0,00	0,00
O14556	G3PT_HUMAN	Glyceraldehyde 3 phosphate dehydrogenase testis specific EC 1 2 1 12 Spermatogenic cell specific	44472	8,23	91	102		266	169		153	36		0,64	0,00	0,00
O95837	GNA14_HUMAN	Guanine nucleotide binding protein alpha 14 subunit G protein alpha subunit 14 Homo sapiens Hum	41544	5,70	369	242		625	392		619	273		0,63	0,00	0,00
P30455	1A36_HUMAN	HLA class I histocompatibility antigen A 36 alpha chain precursor MHC class I antigen A 36 Aw 36	40908	5,94	6665	7515		826	493		224	417		0,60	0,00	0,00
P46776	RL27A_HUMAN	60S ribosomal protein L27a Homo sapiens Human	16551	11,42	924	501		289	170		56	10		0,59	0,00	0,00
Q04917	1433F_HUMAN	14 3 3 protein eta Protein AS1 Homo sapiens Human	28201	4,56	675	1103		212	104		166	45		0,49	0,00	0,00
Q9UF33	EPHA6_HUMAN	Ephrin type A receptor 6 precursor EC 2 7 10 1 Tyrosine protein kinase receptor EHK 2 EPH homol	116041	6,42	150	140		1356	641		1501	275		0,47	0,00	0,00
O00116	ADAS_HUMAN	Alkyldihydroxyacetonephosphate synthase peroxisomal precursor EC 2 5 1 26 Alkyl DHAP synthase	72865	7,00	482	193		1177	510		659	21		0,43	0,00	0,00
Q9UKX5	ITA11_HUMAN	Integrin alpha 11 precursor Homo sapiens Human	133526	6,28	348	399		11646	2398		12888	232		0,21	0,00	0,00
O95716	RAB3D_HUMAN	Ras related protein Rab 3D Homo sapiens Human	24251	4,57	2600	2257		643	84		377	7		0,13	0,00	0,00
Q9BRK4	LZTS2_HUMAN	Leucine zipper putative tumor suppressor 2 Protein LAPSER1 Homo sapiens Human	72714	6,09	184	114		4365	290		5569	123		0,07	0,00	0,00
P05534	1A24_HUMAN	HLA class I histocompatibility antigen A 24 alpha chain precursor MHC class I antigen A 24 Aw 24	40663	5,88	8523			50			4			0,00		0,00
P30486	1B48_HUMAN	HLA class I histocompatibility antigen B 48 alpha chain precursor MHC class I antigen B 48 Bw 48	40337	5,59	4986			93			44			0,00		0,00
Q8N9F8	ZN454_HUMAN	Zinc finger protein 454 Homo sapiens Human	60029	8,64	20			121			27			0,00		0,00
P50148	GNAQ_HUMAN	Guanine nucleotide binding protein G q subunit alpha Guanine nucleotide binding protein alpha q	41441	5,46	173			157			46			0,00		0,00
P30512	1A29_HUMAN	HLA class I histocompatibility antigen A 29 alpha chain precursor MHC class I antigen A 29 Aw 19	40837	6,56	9893			182			5			0,00		0,00
P30508	1C12_HUMAN	HLA class I histocompatibility antigen Cw 12 alpha chain precursor MHC class I antigen Cw 12 Ho	40860	5,87	9763			197			66			0,00		0,00
P14649	MYL6B_HUMAN	Myosin light polypeptide 6B Smooth muscle and nonmuscle myosin light chain alkali 6B Myosin light	22749	5,42	598			211			49			0,00		0,00
P62888	RL30_HUMAN	60S ribosomal protein L30 Homo sapiens Human	12775	9,95	1788			212			66			0,00		0,00
Q92930	RAB8B_HUMAN	Ras related protein Rab 8B Homo sapiens Human	23569	9,43	4186			229			123			0,00		0,00
P59190	RAB15_HUMAN	Ras related protein Rab 15 Homo sapiens Human	24375	5,39	759			234			16			0,00		0,00
Q13637	RAB32_HUMAN	Ras related protein Rab 32 Homo sapiens Human	24981	6,08	357			248			51			0,00		0,00
P62081	RS7_HUMAN	40S ribosomal protein S7 Homo sapiens Human	22113	10,58	365			255			11			0,00		0,00
P19105	MLRM_HUMAN	Myosin regulatory light chain 2 nonsarcomeric Myosin RLC Homo sapiens Human	19781	4,45	935			270			20			0,00		0,00
P17081	RHOQ_HUMAN	Rho related GTP binding protein RhoQ precursor Ras related GTP binding protein TC10 Homo sapiens	22644	5,90	1360			275			141			0,00		0,00
P62136	PP1A_HUMAN	Serine threonine protein phosphatase PP1 alpha catalytic subunit EC 3 1 3 16 PP 1A Homo sapien	37487	5,89	517			278			128			0,00		0,00
P18615	NELFE_HUMAN	Negative elongation factor E NELF E RNA binding protein RD Homo sapiens Human	43213	9,73	278			284			95			0,00		0,00
Q9H3Z4	DNJC5_HUMAN	DnaJ homolog subfamily C member 5 Cysteine string protein CSP Homo sapiens Human	22134	4,74	718			292			97			0,00		0,00
Q8N7M2	ZN283_HUMAN	Zinc finger protein 283 Homo sapiens Human	70179	8,61	7			292			37			0,00		0,00
P09493	TPM1_HUMAN	Tropomyosin 1 alpha chain Alpha tropomyosin Homo sapiens Human	32688	4,49	812			297			29			0,00		0,00
Q9BUF5	TBB6_HUMAN	Tubulin beta 6 chain Homo sapiens Human	49825	4,58	482			314			67			0,00		0,00
Q9HCL3	ZFP14_HUMAN	Zinc finger protein 14 homolog Zfp 14 Zinc finger protein 531 Homo sapiens Human	63422	8,56	101			353			126			0,00		0,00
RANDOM1568	RANDOM15688	Random Sequence 15688	38031	4,53	393			354			266			0,00		0,00
O00161	SNP23_HUMAN	Synaptosomal associated protein 23 SNAP 23 Vesicle membrane fusion protein SNAP 23 Homo sapien	23339	4,70	810			363			19			0,00		0,00
P30453	1A34_HUMAN	HLA class I histocompatibility antigen A 34 alpha chain precursor MHC class I antigen A 34 Aw 34	41029	5,86	8918			364			539			0,00		0,00
Q6IQ22	RAB12_HUMAN	Ras related protein Rab 12 Homo sapiens Human	27231	8,51	1542			373			365			0,00		0,00
P09211	GSTP1_HUMAN	Glutathione S transferase P EC 2 5 1 18 GST class pi GSTP1 1 Homo sapiens Human	23341	5,28	479			378			238			0,00		0,00

P27482	CALL3_HUMAN	Calmodulin like protein 3 Calmodulin related protein NB 1 CaM like protein CLP Homo sapiens	16879	4,10	349			389			72			0,00		0,00
P51809	SYBL1_HUMAN	Synaptobrevin like protein 1 Tetanus insensitive VAMP Ti VAMP Ti VAMP VAMP7 Homo sapiens Hu	24918	8,73	415			394			64			0,00		0,00
O95297	MPZL1_HUMAN	Myelin protein zero like protein 1 precursor Protein zero related Homo sapiens Human	29064	8,98	669			399			135			0,00		0,00
P63027	VAMP2_HUMAN	Vesicle associated membrane protein 2 VAMP 2 Synaptobrevin 2 Homo sapiens Human	12640	8,70	7376			417			188			0,00		0,00
P13726	TF_HUMAN	Tissue factor precursor TF Coagulation factor III Thromboplastin CD142 antigen Homo sapien	33046	6,87	685			418			185			0,00		0,00
Q8NDQ6	ZN540_HUMAN	Zinc finger protein 540 Homo sapiens Human	77044	9,81	18			435			83			0,00		0,00
Q9HC07	TM165_HUMAN	Transmembrane protein 165 Transmembrane protein TPARL Transmembrane protein PT27 Homo sapiens	34883	6,62	491			440			36			0,00		0,00
P15291	B4GT1_HUMAN	Beta 1 4 galactosyltransferase 1 EC 2 4 1 Beta 1 4 GalTase 1 Beta4Gal T1 b4Gal T1 UDP gal	43892	8,71	491			468			57			0,00		0,00
P21291	CSRP1_HUMAN	Cysteine and glycine rich protein 1 Cysteine rich protein 1 CRP1 CRP Homo sapiens Human	20553	8,55	1952			484			84			0,00		0,00
P62424	RL7A_HUMAN	60S ribosomal protein L7a Surfeit locus protein 3 PLA X polypeptide Homo sapiens Human	29977	11,06	969			495			135			0,00		0,00
O75396	SC22B_HUMAN	Vesicle trafficking protein SEC22b SEC22 vesicle trafficking protein homolog B SEC22 vesicle traf	24724	8,73	2400			529			31			0,00		0,00
Q09160	1A80_HUMAN	HLA class I histocompatibility antigen A 80 alpha chain precursor MHC class I antigen A 80 Aw 80	40766	5,86	5519			546			392			0,00		0,00
P51571	SSRD_HUMAN	Translocon associated protein subunit delta precursor TRAP delta Signal sequence receptor subunit	18986	5,72	2041			575			108			0,00		0,00
P54760	EPHB4_HUMAN	Ephrin type B receptor 4 precursor EC 2 7 10 1 Tyrosine protein kinase receptor HTK Homo sapie	108201	6,47	136			616			250			0,00		0,00
P60763	RAC3_HUMAN	Ras related C3 botulinum toxin substrate 3 precursor p21 Rac3 Homo sapiens Human	21365	8,16	3883			625			578			0,00		0,00
Q96S97	MYADM_HUMAN	Myeloid associated differentiation marker SB135 Homo sapiens Human	35250	8,06	585			693			206			0,00		0,00
P30464	1B15_HUMAN	HLA class I histocompatibility antigen B 15 alpha chain precursor MHC class I antigen B 15 Homo	40362	5,86	4836			732			540			0,00		0,00
Q8TAQ5	ZN420_HUMAN	Zinc finger protein 420 Homo sapiens Human	80194	8,55	67			735			866			0,00		0,00
P07686	HEXB_HUMAN	Beta hexosaminidase beta chain precursor EC 3 2 1 52 N acetyl beta glucosaminidase Beta N acety	63071	6,30	282			872			259			0,00		0,00
P35052	GPC1_HUMAN	Glypican 1 precursor Homo sapiens Human	61611	7,01	458			913			376			0,00		0,00
P55290	CAD13_HUMAN	Cadherin 13 precursor Truncated cadherin T cadherin T cad Heart cadherin H cadherin P105	78238	4,61	202			961			72			0,00		0,00
Q92542	NICA_HUMAN	Nicastrin precursor Homo sapiens Human	78361	5,58	232			1055			154			0,00		0,00
Q14344	GNA13_HUMAN	Guanine nucleotide binding protein alpha 13 subunit G alpha 13 Homo sapiens Human	44021	8,00	283			1302			35			0,00		0,00
P01892	1A02_HUMAN	HLA class I histocompatibility antigen A 2 alpha chain precursor MHC class I antigen A 2 Homo s	40896	6,54	12662			1574			200			0,00		0,00
P30504	1C04_HUMAN	HLA class I histocompatibility antigen Cw 4 alpha chain precursor MHC class I antigen Cw 4 Homo	40969	6,01	10388			1592			481			0,00		0,00
O43795	MYO1B_HUMAN	Myosin Ib Myosin I alpha MMI alpha MMIa MYH 1c Homo sapiens Human	131901	9,68	395			1880			363			0,00		0,00
Q96RT1	LAP2_HUMAN	Protein LAP2 Erbb2 interacting protein Erbin Densin 180 like protein Homo sapiens Human	158199	5,17	185			1937			351			0,00		0,00
P03989	1B27_HUMAN	HLA class I histocompatibility antigen B 27 alpha chain precursor MHC class I antigen B 27 Homo	40403	5,44	4055			2163			985			0,00		0,00
P16144	ITB4_HUMAN	Integrin beta 4 precursor GP150 CD104 antigen Homo sapiens Human	202021	5,67	222			3444			124			0,00		0,00
P13647	K2C5_HUMAN	Keratin type II cytoskeletal 5 Cytokeratin 5 CK 5 Keratin 5 K5 58 kDa cytokeratin Homo	62339	7,79		92			485			41			0,00	
Q9H4G0	E41L1_HUMAN	Band 4 1 like protein 1 Neuronal protein 4 1 4 1N Homo sapiens Human	98442	5,29		261			1414			277			0,00	
Q9UBC5	MYO1A_HUMAN	Myosin Ia Brush border myosin I BBM I BBMI Myosin I heavy chain MIHC Homo sapiens Human	118325	9,57		110			928			128			0,00	
P13645	K1C10_HUMAN	Keratin type I cytoskeletal 10 Cytokeratin 10 CK 10 Keratin 10 K10 Homo sapiens Human	59482	4,95		1385			2259			883			0,00	
Q9BQE3	TBA6_HUMAN	Tubulin alpha 6 chain Alpha tubulin 6 Homo sapiens Human	49863	4,79		2689			1278			1016			0,00	
P10321	1C07_HUMAN	HLA class I histocompatibility antigen Cw 7 alpha chain precursor MHC class I antigen Cw 7 Homo	40622	5,63		8725			439			168			0,00	
O95573	ACSL3_HUMAN	Long chain fatty acid CoA ligase 3 EC 6 2 1 3 Long chain acyl CoA synthetase 3 LACS 3 Homo	80368	8,37		406			801			194			0,00	
P30498	1B78_HUMAN	HLA class I histocompatibility antigen B 78 alpha chain precursor MHC class I antigen B 78 Homo	40453	5,80		4969			2772			633			0,00	
P56199	ITA1_HUMAN	Integrin alpha 1 precursor Laminin and collagen receptor VLA 1 CD49a antigen Homo sapiens H	130764	5,86		227			2367			30			0,00	
O14493	CLD4_HUMAN	Claudin 4 Clostridium perfringens enterotoxin receptor CPE receptor CPE R Williams Beuren syn	22062	8,01		795			203			56			0,00	
Q9UIQ6	LCAP_HUMAN	Leucyl cystinyl aminopeptidase EC 3 4 11 3 Cystinyl aminopeptidase Oxytocinase OTase Insuli	117274	5,39		275			1370			80			0,00	
Q08722	CD47_HUMAN	Leukocyte surface antigen CD47 precursor Integrin associated protein IAP Antigenic surface dete	35190	6,98		356			676			15			0,00	
P30481	1B44_HUMAN	HLA class I histocompatibility antigen B 44 alpha chain precursor MHC class I antigen B 44 Bw 44	40456	5,62		2147			414			426			0,00	
Q9Y6M5	ZNT1_HUMAN	Zinc transporter 1 ZnT 1 Solute carrier family 30 member 1 Homo sapiens Human	55292	6,01		342			1010			189			0,00	
P19013	K2C4_HUMAN	Keratin type II cytoskeletal 4 Cytokeratin 4 CK 4 Keratin 4 K4 Homo sapiens Human	57249	6,22		389			507			384			0,00	
P27701	CD82_HUMAN	CD82 antigen Inducible membrane protein R2 C33 antigen IA4 Metastasis suppressor Kangai 1 S	29605	4,96		461			506			135			0,00	
P61073	CXCR4_HUMAN	C X C chemokine receptor type 4 CXC R4 CXCR 4 Stromal cell derived factor 1 receptor SDF 1 re	39719	8,17		567			726			92			0,00	
P41440	S19A1_HUMAN	Folate transporter 1 Solute carrier family 19 member 1 Placental folate transporter FOLT Redu	64827	9,02		446			692			254			0,00	
Q9UM01	YLA1_HUMAN	Y L amino acid transporter 1 y L type amino acid transporter 1 y LAT 1 Y LAT1 Monocyte amin	55954	5,12		23			312			94			0,00	
Q86YS6	RAB43_HUMAN	Ras related protein Rab 43 Ras related protein Rab 41 Homo sapiens Human	23324	5,30		2275			220			72			0,00	
O60762	DPM1_HUMAN	Dolichol phosphate mannosyltransferase EC 2 4 1 83 Dolichol phosphate mannose synthase Dolichyl	29615	9,86		279			448			17			0,00	
P10301	RRAS_HUMAN	Ras related protein R Ras precursor p23 Homo sapiens Human	23465	6,51		1177			467			246			0,00	
P01116	RASK_HUMAN	GTPase KRas precursor K Ras 2 Ki Ras c K ras c Ki ras Homo sapiens Human	21642	6,37		2194			625			50			0,00	
Q9H7P6	F125B_HUMAN	Protein FAM125B Homo sapiens Human	35596	8,16		290			574			210			0,00	
P54762	EPHB1_HUMAN	Ephrin type B receptor 1 precursor EC 2 7 10 1 Tyrosine protein kinase receptor EPH 2 NET HEK	109814	5,98		113			470			257			0,00	
P10314	1A32_HUMAN	HLA class I histocompatibility antigen A 32 alpha chain precursor MHC class I antigen A 32 Homo	41022	6,01		7574			456			441			0,00	
Q9NUM4	T106B_HUMAN	Transmembrane protein 106B Homo sapiens Human	31107	6,59		616			377			199			0,00	
Q9UBR2	CATZ_HUMAN	Cathepsin Z precursor EC 3 4 22 Cathepsin X Cathepsin P Homo sapiens Human	33846	6,74		445			364			51			0,00	
Q9Y3L5	RAP2C_HUMAN	Ras related protein Rap 2c precursor Homo sapiens Human	20731	4,67		650			156			75			0,00	
Q9H3N1	TXND1_HUMAN	Thioredoxin domain containing protein 1 precursor Transmembrane Trx related protein Thioredoxin r	31770	4,72		1149			354			46			0,00	
P07951	TPM2_HUMAN	Tropomyosin beta chain Tropomyosin 2 Beta tropomyosin Homo sapiens Human	32830	4,46		632			353			55			0,00	
Q9NRW1	RAB6B_HUMAN	Ras related protein Rab 6B Homo sapiens Human	23446	5,22		646			345			258			0,00	
P08758	ANXA5_HUMAN	Annexin A5 Annexin V Lipocortin V Endonexin II Calphobindin I CBP I Placental anticoagula	35914	4,73		443			288			99			0,00	
P59998	ARPC4_HUMAN	Actin related protein 2 3 complex subunit 4 ARP2 3 complex 20 kDa subunit p20 ARC Homo sapiens	19654	8,78		639			283			37			0,00	
P10114	RAP2A_HUMAN	Ras related protein Rap 2a precursor RbBP 30 Homo sapiens Human	20602	4,53		369			136			89			0,00	
Q9Y2B0	MSAP_HUMAN	MIR interacting saposin like protein precursor Transmembrane protein 4 Putative secreted protein	20639	4,62		343			247			209			0,00	
Q9Y281	COF2_HUMAN	Cofilin 2 Cofilin muscle isoform Homo sapiens Human	18724	8,16		849			117			28			0,00	
P18464	1B51_HUMAN	HLA class I histocompatibility antigen B 51 alpha chain precursor MHC class I antigen B 51 Homo	40541	5,94		5300			163			80			0,00	
Q31612	1B73_HUMAN	HLA class I histocompatibility antigen B 73 alpha chain precursor MHC class I antigen B 73 Homo	40409	5,80		5446			118			18			0,00	

Figure S1: Quantitative Mass spectrometry detected enrichment of cellular proteins in endosomal fractions after HPV16 PsV incubation. The quantitative analysis of host-cell proteins of two individual LC-MS experiments is shown. Experiments were performed using HeLa cells. Three independent preparations at different timepoints (non-infected, 4h, 7h post infection) were analysed in five technical replicates in each experiment. Parts per million (ppm) values are calculated at the protein level. The amount of each protein is determined using the 3 "best ionizing" peptides, which are then compared to the total protein in the sample. The protein level values of the technical replicates (MW ppm) are highlighted in yellow; the data for HPV16 major capsid protein L1 are highlighted in red; for p62/sequestosome-1 in green and for Rab5a in gray.

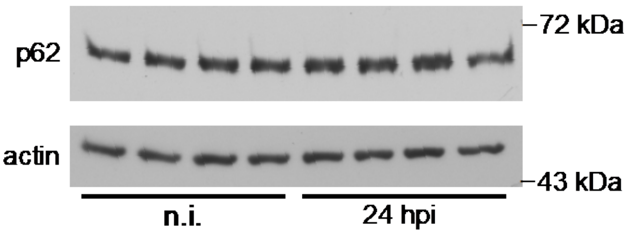

Figure S2. Representative p62 levels after HPV16 PsVs addition. HeLa cells were incubated with HPV16 PsVs for 24 hours (24 hpi) or left untreated (not infected, n.i.). P62 levels were analyzed by Western blotting using p62-specific mAb. β-actin was used as loading control.

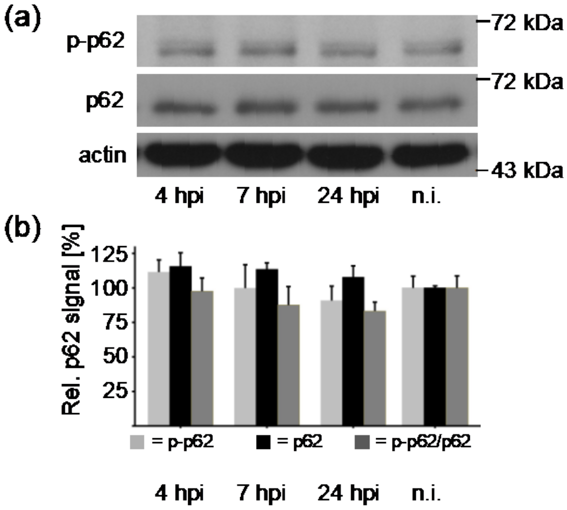

Figure S3. P62 phosphorylation is unaffected by HPV16 PsVs. (**a**) Western blotting showing p62 phosphorylation status of HeLa cells upon HPV16 PsVs addition. Cells were starved for 1 hour and subsequently exposed to PsVs for different time points. All samples were collected at the same time. For signal detection, the membrane was stained using p62-specific mAb (for total p62 protein detection) and phospho-p62-specific pAb (p-p62, for detection of phosphorylated p62 at Ser 349). β-actin was used as a loading control. (**b**) Quantification of the blots shown in (**a**) using ImageJ software. The amount of phosphorylated p62 protein is shown as a ratio of phosphorylated to total p62 form. The values are given as mean ± SEM and the mean for not infected cells (n.i.) was set to 100%**.** Data (n =

4-9 out of three replicates) were analyzed for significant differences using unpaired *t*-test for total p62 ($p = 0.0176$, $p = 0.0017$ and $p = 0.1884$ for n.i. vs. 4 hpi, 7 hpi and 24 hpi, respectively), using unpaired *t*-test for phospho-p62 ($p = 0.4375$ and $p = 0.9878$ for n.i. vs. 4 hpi and 7 hpi, respectively) and Mann-Whitney test ($p = 0.3301$ for n.i. vs. 24 hpi), and using unpaired *t*-test for phospho-p62 per total p62 ($p = 0.8681$, $p = 0.4446$ and $p = 0.2509$ for n.i. vs. 4 hpi, 7 hpi and 24 hpi, respectively).

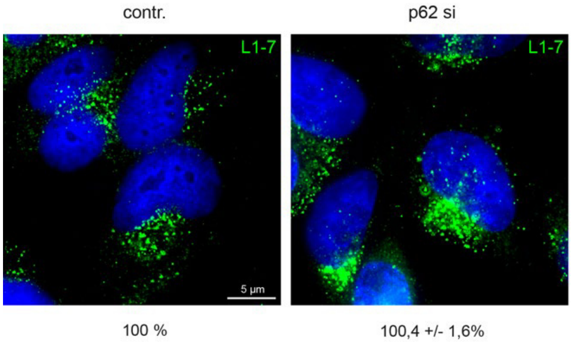

Figure S4. P62 knockdown has no effect on virus internalization. HeLa cells were transfected with control siRNA (contr.) or a p62 siRNA pool (p62 si) for 48 hours and infected with HPV16 PsVs for 7 hours. Cells were stained with L1-specific Ab (33L1-7) and nuclear stain solution (Hoechst 33342). Relative L1-7-positive pixels per DNA pixels were quantified using ImageJ software and the values are given as a mean ± SEM. The mean for control siRNA-treated cells (contr.) was set to 100%.

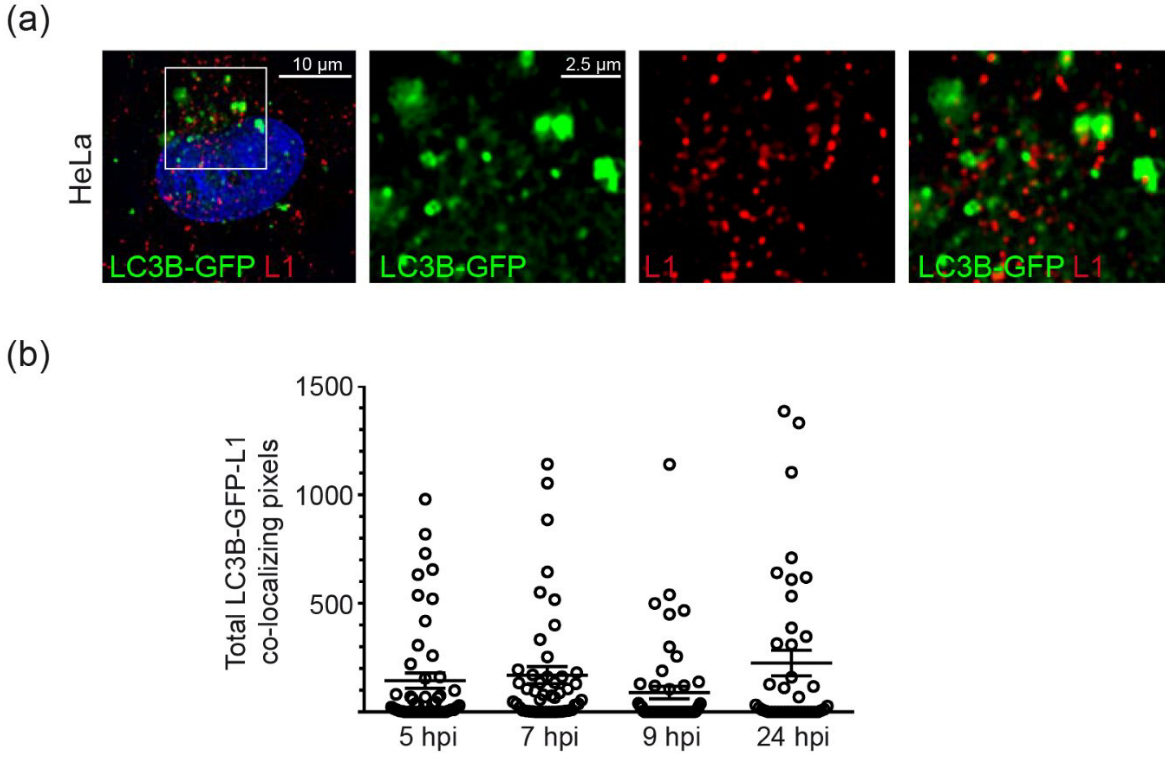

Figure S5. HPV16 major capsid protein L1 co-localizes stably over the time with autophagosome marker LC3B. (**a**, **b**) HeLa cells were transfected with LC3B-GFP expressing plasmid for 24 hours and infected with HPV16 PsVs for five, seven, nine or 24 hours (5h, 7h, 9h, or 24h). Cells were stained for HPV16 major capsid protein L1 by immunofluorescence using K75 antibody. (**a**) Representative pictures of LC3B-GFP and HPV16 L1 co-localization in HeLa (exposed to PsVs for 7 hours). LC3B-GFP is shown in green, L1 in red and nuclear DNA (stained by Hoechst) in blue. The box in the first panel outlines the following higher magnification images. (**b**) LC3B-GFP-L1 co-localization is not dependent on the duration of HPV16 infection in HeLa cells. Graphs show quantification of the total number of pixels representing LC3B-GFP-L1 co-localization after 5h, 7h, 9h, or 24 h of HPV16 PsVs. Data (n = 40 – 52 out of three replicates) are shown as scatter dot plot and the lines represent mean ± SEM of the absolute numbers from three independent experiments. Significant differences were analyzed using ordinary one-way ANOVA ($p = 0.1252$).

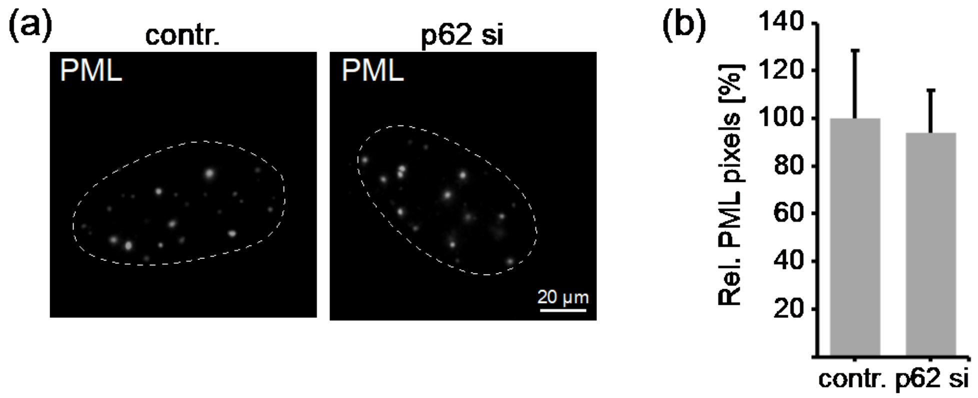

Figure S6. PML after p62 knockdown. (**a**-**b**) HeLa cells were transfected with control siRNA (contr.) or a p62 siRNA pool (p62 si) for 48 hours and infected with HPV16 PsVs for 24 hours. Cells were stained with PML-specific mAb and nuclear stain solution (Hoechst 33342). Representative pictures are shown in (**a**). (**b**) Relative PML-positive pixels per DNA pixels were quantified using ImageJ software and are given as a mean ± SEM. The mean for control siRNA-treated cells (contr.) was set to 100%. Data (n = 57-60 out of three replicates) were analyzed for significant differences using Mann-Whitney test: $p > 0.05$.
